# Addressing Fecal Contamination in Rural Kenyan Households: The Roles of Environmental Interventions and Animal Ownership

**DOI:** 10.1021/acs.est.3c09419

**Published:** 2024-05-17

**Authors:** Jenna M. Swarthout, Maryanne Mureithi, John Mboya, Benjamin F. Arnold, Marlene K. Wolfe, Holly N. Dentz, Audrie Lin, Charles D. Arnold, Gouthami Rao, Christine P. Stewart, Thomas Clasen, John M. Colford, Clair Null, Amy J. Pickering

**Affiliations:** † Department of Civil and Environmental Engineering, Tufts University, Medford, Massachusetts 02155, United States; ‡ Innovations for Poverty Action, Nairobi 00200, Kenya; § Department of Civil and Environmental Engineering, University of California, Berkeley, Berkeley, California 94720, United States; ∥ Francis I. Proctor Foundation, Department of Ophthalmology and Institute for Global Health Sciences, University of California, San Francisco, San Francisco, California 94158, United States; ⊥ Gangarosa Department of Environmental Health, Emory University, Atlanta, Georgia 30322, United States; # Institute for Global Nutrition, University of California, Davis, Davis, California 95616, United States; ∇ Department of Microbiology and Environmental Toxicology, University of California, Santa Cruz, Santa Cruz, California 95064, United States; ○ Department of Environmental Sciences and Engineering, University of North Carolina at Chapel Hill, Chapel Hill, North Carolina 27599, United States; ◆ Mathematica, Washington, District of Columbia 20002, United States; ¶ Chan Zuckerberg Biohub San Francisco, San Francisco, California 94158, United States; ▼ School of Public Health, Division of Epidemiology, University of California, Berkeley, Berkeley, California 94720, United States

**Keywords:** water, sanitation, handwashing, WASH, transmission pathway, Escherichia coli, diarrhea, stunting

## Abstract

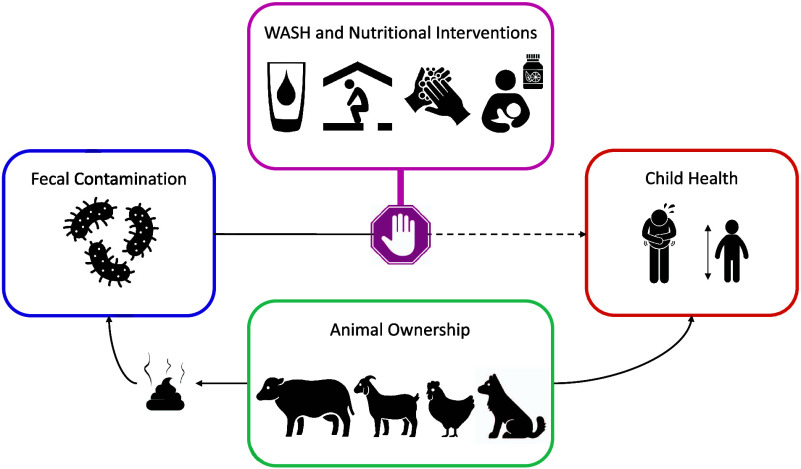

Combined water, sanitation, and handwashing (WSH) interventions could reduce fecal contamination along more transmission pathways than single interventions alone. We measured *Escherichia coli* levels in 3909 drinking water samples, 2691 child hand rinses, and 2422 toy ball rinses collected from households enrolled in a 2-year cluster-randomized controlled trial evaluating single and combined WSH interventions. Water treatment with chlorine reduced *E. coli* in drinking water. A combined WSH intervention improved water quality by the same magnitude but did not affect *E. coli* levels on hands or toys. One potential explanation for the limited impact of the sanitation intervention (upgraded latrines) is failure to address dog and livestock fecal contamination. Small ruminant (goat or sheep) ownership was associated with increased *E. coli* levels in stored water and on child hands. Cattle and poultry ownership was protective against child stunting, and domesticated animal ownership was not associated with child diarrhea. Our findings do not support restricting household animal ownership to prevent child diarrheal disease or stunting but do support calls for WSH infrastructure that can more effectively reduce household fecal contamination.

## Abstract 2

This study reports limited impacts of water, sanitation, and handwashing interventions on reducing fecal contamination along environmental transmission pathways. Animal ownership may increase fecal contamination in households while protecting against child stunting.

## Introduction

Diarrheal disease remains a leading cause of under-5 child mortality in low- and middle-income countries (LMICs).^1^ Chronic diarrhea is also associated with stunting (length-for-age *Z*-scores >2 standard deviations below the median of the World Health Organization (WHO) growth standard for age and gender); stunting affected an estimated 144 million children under 5 years globally in 2020 and is targeted under Goal 2 (Zero Hunger) of the Sustainable Development Goals.^2−4^ The Sustainable Development Goals have encouraged significant investments to increase access to safe drinking water, sanitation, and handwashing (WSH) and reduce the burden of infectious disease by 2030.^4^ WSH interventions are hypothesized to interrupt the transmission of fecal pathogens in the household environment, thereby reducing the risk of diarrhea.^5^ Previous studies have predominately used observational data to study associations between fecal indicator bacteria in the household environment (e.g., in water, on hands, in soil, on surfaces), child health outcomes (e.g., diarrhea, stunting), and the quality of WSH infrastructure.^6−11^ However, the ability to infer causal effects from observational studies is limited, as study participants cannot be assigned to randomized treatment and control groups; the lack of randomization hinders the capability to dismiss potential effects caused by unmeasured confounders.

A large number of randomized controlled trials (RCTs) have assessed the effects of water treatment interventions on fecal indicator bacterial levels in drinking water, but few sanitation and handwashing RCTs have measured environmental indicators of fecal contamination.^12,13^ We identified two RCTs evaluating the effects of handwashing interventions on hand contamination, neither of which reduced bacterial levels in hands.^14,15^ Due to high temporal heterogeneity in hand contamination, random hand rinse samples, as were collected in these two RCTs, may be poor proxy measures for handwashing behavior around critical events for bacterial transmission (e.g., eating).^16,17^ A 2016 systematic review concluded there was no evidence that improved sanitation reduces fecal contamination levels in water, on hands, on sentinel toys, on household surfaces, or in soil, although inherent heterogeneity of settings, interventions and their adoption/coverage, and methods between studies prevented the authors from conducting meta-analyses; only one study included in the review (Clasen et al.) was an RCT that simultaneously measured microbiological indicators across multiple pathways (water, hand rinses, toy rinses, flies).^18,19^ Recent RCTs have also demonstrated mixed effects of WSH interventions on diarrhea and stunting.^20−22^ It is therefore hypothesized (partially based on previous observational and modeling studies) that combining WSH interventions, rather than delivering them in isolation as has been done in most previous trials, could improve microbial contamination and promote health benefits.^23−28^

The lack of observed effects on child health outcomes could partially be explained by insufficient adherence to interventions or community-level coverage of interventions, while an additional explanation could be the potential negative health effects of exposure to animal feces, which has historically been neglected in the design of WSH interventions.^29−31^ Previous research has shown that animal feces contribute to fecal contamination in the domestic environment. A study in rural Bangladesh (Bangladesh WASH Benefits study) found that *Escherichia coli* concentrations were higher in soil, stored water, and food sampled from compounds with animals compared to those without animals.^32^ Host-specific fecal markers from animals (dogs, poultry, ruminants) were detected in multiple household reservoirs (soil, hands, stored water) in rural and urban Bangladesh, rural India, and rural Kenya.^32−37^ In all sample types tested, animal fecal markers were more prevalent than human fecal markers. In rural Bangladesh, increased concentration of an animal-specific fecal marker (BacCow) was associated with increased prevalence of pathogenic *E. coli*.^35^ Allowing animals to freely roam and graze is a common animal husbandry practice in many low-income communities. Nonanimal-owning households might therefore also be at a high risk of exposure to fecal contamination from animal feces. Previous work in Bangladesh has documented ruminant-specific fecal markers on child hands or floor sponge samples in both goat-owning and nonruminant-owning compounds.^34^ However, as most animal ownership studies have focused on household-level ownership, little is known about the impact of community-level ownership on contamination in domestic environments.

While animal ownership can put humans at an elevated risk of exposure to animal fecal contamination, current evidence suggests that rural household livestock ownership likely provides child nutritional benefits.^38−41^ This might partially be due to livestock ownership directly increasing household consumption of animal-sourced foods. Socioeconomic pathways might also be important. For example, large livestock (e.g., cattle) ownership could be a proxy for higher household socioeconomic status. Small livestock (e.g., poultry) ownership could increase women’s empowerment; as women are often the primary caretakers of household poultry, greater participation in livestock markets might increase women’s control over making purchasing decisions for child caretaking.^42^ High-intensity animal exposure (e.g., housing livestock in child sleeping quarters), however, has been linked to poor child health outcomes, including diarrheal disease, environmental enteric dysfunction (EED), and growth faltering.^38,43,44^ There is thus a need to contextualize the role of animals in domestic fecal contamination alongside environmental intervention trials to understand whether interventions may reduce child exposure to human and animal feces, without introducing barriers to nutritional benefits from household animal ownership.

The Kenya WASH Benefits study was a cluster-randomized controlled trial designed to test the effects of water, sanitation, handwashing, and nutritional interventions, alone and in combination, on child diarrhea prevalence, linear growth, parasitic infections, biomarkers of EED, and child development.^22,45−48^ The trial’s primary outcomes have been reported: WSH interventions, whether separately or in combination, did not reduce child diarrhea or improve child growth during the trial.^22^ To assess the extent to which the interventions may have reduced child exposure to fecal contamination—a key intermediate step to health outcomes—we nested environmental sample collection within a subset of enrolled households in selected trial arms. Our aim was to determine if the interventions reduced levels of fecal indicator bacteria in the domestic environment along likely exposure pathways for young children. Further, we leveraged data from the study to contextualize the role of animals in domestic contamination by assessing whether household-level animal ownership was associated with (1) higher *E. coli* prevalence and concentrations in the domestic environment (stored water, child hand rinses, sentinel toy rinses) and fly prevalence and densities near food preparation areas; and (2) under-5 child health outcomes (diarrhea, stunting). We also assessed whether community-level (village-level) animal ownership was associated with environmental contamination and child health outcomes.

## Materials and Methods

### Study Design

The Kenya WASH Benefits trial enrolled pregnant women in Kakamega, Bungoma, and Vihiga counties of rural Western Kenya and followed the children born to these pregnancies (including twins) for 2 years. Households in this region are often organized into compounds, where multiple related families live together in a defined area and share latrines and communal areas for cooking, child play, and domestic animal grazing/housing. A random number generator with reproducible seed was used to assign interventions to household clusters; groups of nine geographically adjacent clusters were block-randomized into a passive control arm, active control arm, or one of six intervention arms (chlorinated drinking water; improved sanitation through pit latrine upgrades with a reinforced slab and drop hole cover, child potties, and scoops for removing human and animal feces from the homes and yards of enrolled children; handwashing with soap; combined WSH interventions; small quantity lipid-based nutrient supplementation; combined WSH and nutritional (WSHN) interventions). Study design details for the trial, including details regarding the passive control arm, active control arm (including monthly visits by community health promoters), six intervention arms, and behavior change messaging through monthly visits for all intervention arms (including infant and young child feeding counseling for the nutrition and WSHN arms) were previously published (Supporting Information S1 and Figure S1).^21,22,45^

### Sample and Data Collection and Processing

**Figure 1:**
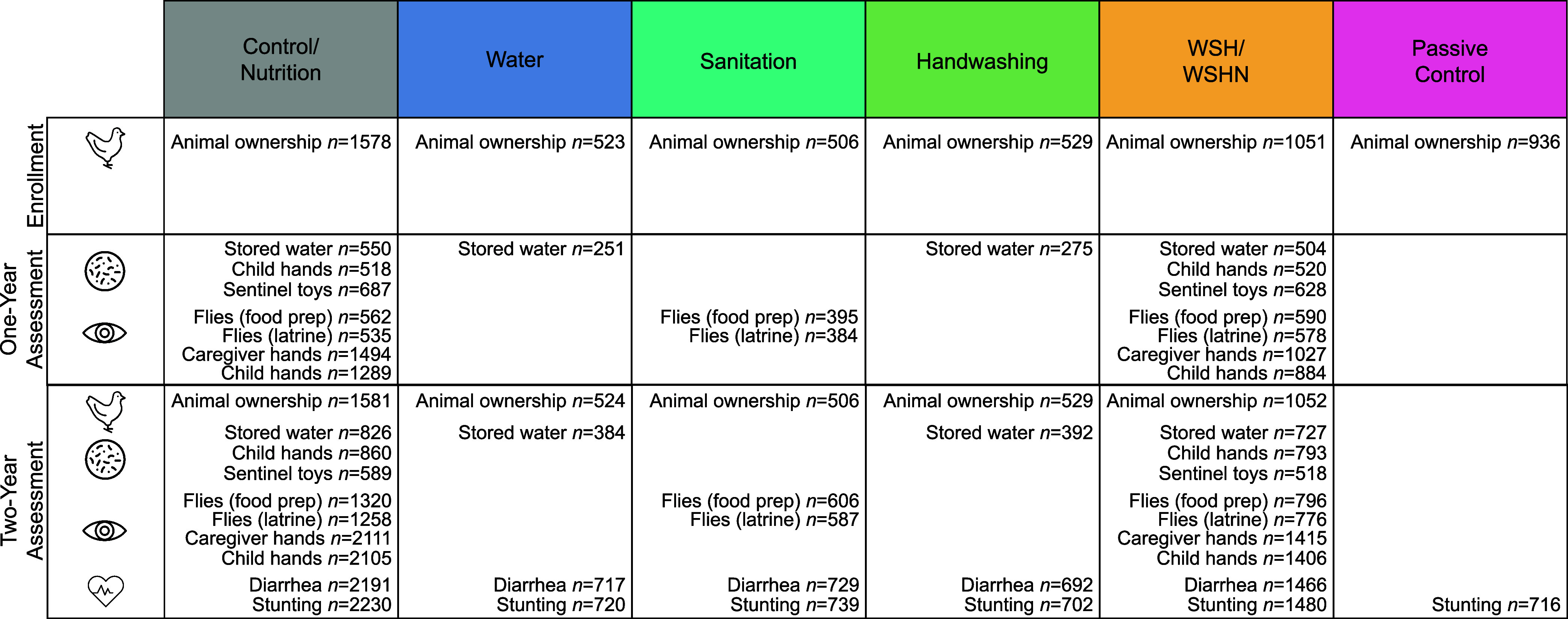
Data collection and environmental sampling profile in the trial, by study arm and year of measurement. Chicken indicates animal ownership data collection; bacterial plate (circle with dots) indicates *E. coli* measurements; eye indicates rapid observations by field staff; heart indicates child health data collection. The majority of measurements were conducted in the control and nutrition (C/N) and combined water, sanitation, and handwashing (WSH/WSHN) study arms.

Approximately 1 year and 2 years after intervention delivery (timed to match the collection of the trial’s primary child health outcomes), we assessed environmental contamination in a subset of approximately 1500 households (approximately 375 children from each of the control, nutrition, combined WSH, and combined WSHN arms) that participated in EED biomarker measurement and comprised the EED cohort of the trial (Figure 1). The intensive environmental contamination assessment was conducted in the EED subgroup because of the added benefit of being able to assess relationships between environmental contamination and EED biomarkers, as well as leveraging the multivisit data collection infrastructure that was already in place for the cohort. The intensive assessment was comprised of stored drinking water samples, child hand and sentinel plastic toy ball (after ∼24 h of play) rinses, fly enumeration near food preparation areas and latrines, and visible hand cleanliness observations for mothers and children. Child hand and sentinel toy rinse samples were collected as proxy indicators of the overall environmental fecal contamination in the household and representatives of likely exposure pathways for children under 2 years old.^49^ We also measured selected indicators of environmental contamination in similar sized subsets of households enrolled in the single water, sanitation, and handwashing arms (Figure 1). Specifically, stored drinking water samples were collected from the water and handwashing arms, and fly densities near food preparation areas and latrines were measured in the sanitation arm. Stored drinking water was sampled in the handwashing arm because previous evidence suggests that caregiver hand and stored water contamination are highly correlated, and stored drinking water fecal bacterial levels are typically less variable than hand rinse fecal bacterial levels.^8,16,17^

All environmental samples (stored water, child hand rinses, sentinel toy rinses) were collected according to previously published protocols, analyzed by membrane filtration, and incubated on MI media to isolate and enumerate *E. coli*.^8,50^ Fly densities (counts) were measured at food preparation areas and latrines using the scudder fly grill method; flies were classified as house, bottle, flesh, or “other” species. Field staff observed, as a binary measure, whether there was visible dirt on mothers’ and children’s hands (palms, fingerpads, underneath fingernails). Additional sample collection and processing details are provided in Supporting Information S1.

Household animal ownership data, including binary ownership and number and type of animals owned, were recorded at the time of enrollment and 2 years after intervention delivery (Figure 1). Data on child health outcomes (diarrhea, linear growth) were also collected 2 years after intervention delivery. Caregiver-reported diarrhea was defined as three or more watery stools in 24 h or a single episode of blood in the stool within the past 7 days. Anthropometric measurements—including length, head circumference, and weight—were taken by trained field workers following standard protocols.^51,52^ Reported child dates of birth were verified, when available, against clinic cards, health booklets, or other birth records (e.g., baptismal card). *Z*-scores were calculated for length-for-age using WHO child growth standards; outliers were excluded following WHO recommendations.^53^

### Statistical Analyses

#### Intervention Effects on Environmental Contamination

Considering that improved nutrition would not be expected to influence environmental contamination, for models evaluating intervention impacts and associations between household animal ownership and environmental contamination, we grouped measurements collected from the WSH and WSHN arms together (WSH/WSHN) as well as measurements collected from the control and nutrition arms together (C/N). We published a prespecified statistical analysis plan for evaluating WSH intervention impacts on fecal contamination in household environments (https://osf.io/eg2rc/). All intervention impact statistical analyses were independently replicated by two different authors (AJP, JMS). Outcomes measured in the single intervention arms (water, sanitation, handwashing) and pooled combined arms (WSH/WSHN) were compared to outcomes in the pooled control (C/N) arms. We estimated unadjusted and adjusted intention-to-treat effects between arms, relying on the unadjusted analysis as our primary analysis. We estimated log reductions, prevalence ratios, and prevalence differences using generalized linear models with robust standard errors, with cluster as the independent unit. We used the modified Poisson regression for binary outcomes.^54,55^ All models included fixed effects for randomization block (to take advantage of the pair-matched design). In adjusted analyses, we used targeted maximum likelihood estimation to adjust for prespecified baseline covariates (e.g., the most recent time it rained for all outcomes and how much children played with balls for toy rinses), including only variables strongly associated with the outcome (a full list of covariates is available in Supporting Information S1).^56^ We conducted subgroup analyses by year of data collection (year 1 versus year 2).

#### Associations between Animal Ownership and Environmental Contamination and Child Health

As post hoc analyses, we estimated unadjusted and adjusted associations between household animal ownership and household environmental contamination and child health outcomes, relying on the analyses adjusted for relevant covariates (illustrated in directed acyclic graphs for each outcome in Figures S2–S7) as our primary analyses. The measures of animal ownership for the primary analyses were the log_10_-transformed number of animals owned overall and by species (cattle, goats, sheep, poultry, dogs); results from analyses using binary animal ownership are presented in the Supporting Information. We evaluated associations between cross-sectional (year 2) animal ownership and environmental contamination outcomes (*E. coli* in water, hand rinses, and toy rinses; flies at food preparation areas) and acute diarrhea, for which recent child exposure to animal feces was the hypothesized exposure pathway. We relied on a cross-sectional rather than prospective approach for environmental contamination outcomes due to high temporal variability in fecal indicator bacteria, which are strongly dependent on environmental conditions.^16,57^ However, we leveraged prospective (at the time of enrollment) animal ownership data to assess associations with linear growth outcomes (stunting; continuous length-for-age *Z*-scores). Although the passive control arm was excluded from year 2 stool collection activities due to budgeting reasons in the parent trial (during which animal ownership and diarrhea data were collected), when assessing associations between baseline animal ownership and child growth, data from the passive control arm were still leveraged to increase statistical power. Sheep ownership data was not collected at enrollment. Covariates, including intervention arm, were prescreened for their association with each outcome (likelihood ratio test *p*-value <0.2) and variation (prevalence ≥5%). Nutritional intervention arms were pooled as above (WSH/WSHN; C/N) for environmental contamination outcomes, and all intervention arms were kept separate for child health outcomes. We used generalized linear models with robust standard errors to account for village-level clustering in the parent trial. We used modified Poisson regressions with log links to estimate prevalence ratios and linear probability models to estimate prevalence differences for binary outcomes; we also used linear probability models to estimate differences in log-transformed bacterial counts for *E. coli* concentration and fly counts for fly density. For examining the association between animal ownership and *E. coli* prevalence and concentration in stored water, we excluded households with detectable free chlorine in their stored drinking water at year 2, as detectable chlorine in water samples could mask the associations between animal ownership and water contamination. A sensitivity analysis, which included households with detectable free chlorine (adjusted for the presence of chlorine in stored water), had similar results to excluding households with free chlorine in their drinking water (Figure S8).

We conducted several sensitivity analyses as robustness checks for the models examining associations between animal ownership and environmental contamination and child health. To account for the larger number of poultry owned by households compared to other species (cattle, goats, sheep, dogs), we calculated Tropical Livestock Unit (TLU) scores, which are commonly used weighted measures of domesticated animals.^58,59^ We adapted the following weighting factors for different animal species across sub-Saharan Africa based on their metabolic weights, as recommended by Njuki et al. in 2011:^59^ 0.5 for cattle, 0.1 for sheep and goats, 0.02 for poultry (chickens, duck, geese, turkey), and 0.1 for dogs. Similar to previous studies, we created five categories of TLU scores to represent varying livestock compositions across households as follows: (1) no animals (0 TLU), (2) a few chickens (0.02–0.08 TLU), (3) chickens or dogs or small ruminants (0.10–0.48), (4) 1–2 cattle or ≥10 poultry (0.50–1.48 TLU), and (5) ≥3 cattle (≥1.5 TLU).^40,41^ When assessing binary animal ownership as an exposure, we also excluded observations with extreme predicted probabilities of animal ownership (within 1% of the minimum and maximum predicted probabilities across all households). SuperLearner was used to estimate the predicted probability of animal ownership for all covariates considered and to discover and prevent positivity violations (lack of variation in animal ownership in a specific covariate stratum).^60^ For the prospective analyses of linear growth outcomes, we conducted three different sensitivity analyses: (1) excluding households that changed animal ownership status between enrollment and 2 years after intervention delivery, (2) reclassifying households that obtained animals during the study as animal-owning households, and (3) conducting subgroup analyses on households that went from nonanimal-owning to animal-owning status and vice versa.

As a secondary analysis, we estimated unadjusted and adjusted associations between all outcomes and community-level animal ownership (proxied by the median number of animals owned by study households living in the same village).

## Results and Discussion

Figure 1 illustrates the number of measurements collected for each outcome by study arm and year of measurement.

### Levels of Environmental Contamination in the Control Group

When combining the 1- and 2-year assessments, 94% of stored drinking water samples were contaminated with *E. coli* (mean: 1.48 log_10_ colony forming units [CFU]/100 mL, standard deviation (SD): 1.43, 1.54), 90% of child hands were contaminated with *E. coli* (mean: 1.74 log_10_ CFU/100 mL, SD: 1.63, 1.85), and 73% of toys were contaminated with *E. coli* (mean: 0.58 log_10_ CFU/toy, SD: 0.51, 0.65) in the control group (C/N) (Table S1). One quarter (26%) of caregivers had visible dirt observed on their palms or fingerpads, and over half (54%) had dirt observed underneath their fingernails (Table S3). Approximately one-third (36%) of children had visible dirt observed on their palms or fingerpads, while two-thirds (67%) had dirt observed underneath their fingernails. Flies were present at 62% of food preparation areas (mean: 3.4 flies, SD: 6.2) and at 67% of latrines (mean: 3.7 flies, SD: 5.5) (Table S3). House flies were more common at food preparation areas (60% prevalence) than latrines (30%); bottle flies were observed at 3% of food preparation areas but were observed at more than half of latrines (55% prevalence); flesh flies were observed at <1% of food preparation areas and 3% of latrines (Table S4).

### Intervention Effects on Environmental Contamination

We previously reported indicators of intervention uptake.^22^ In water intervention arms, the proportion of households with detectable chlorine residual in their stored drinking water ranged from 39 to 43% at year 1 and from 19 to 23% at year 2. Among households in the sanitation intervention arms, the proportion with access to an improved latrine was 89–90% at year 1 and 78–82% at year 2. In handwashing intervention arms, soap and water were present at a handwashing location at 76–78% of households at year 1 and at 19–23% at year 2. Intervention effects on environmental contamination in stored water, on child hands, and on sentinel toys, as well as on flies near latrines and food preparation areas, are reported below.

#### Water Quality

**Figure 2:**
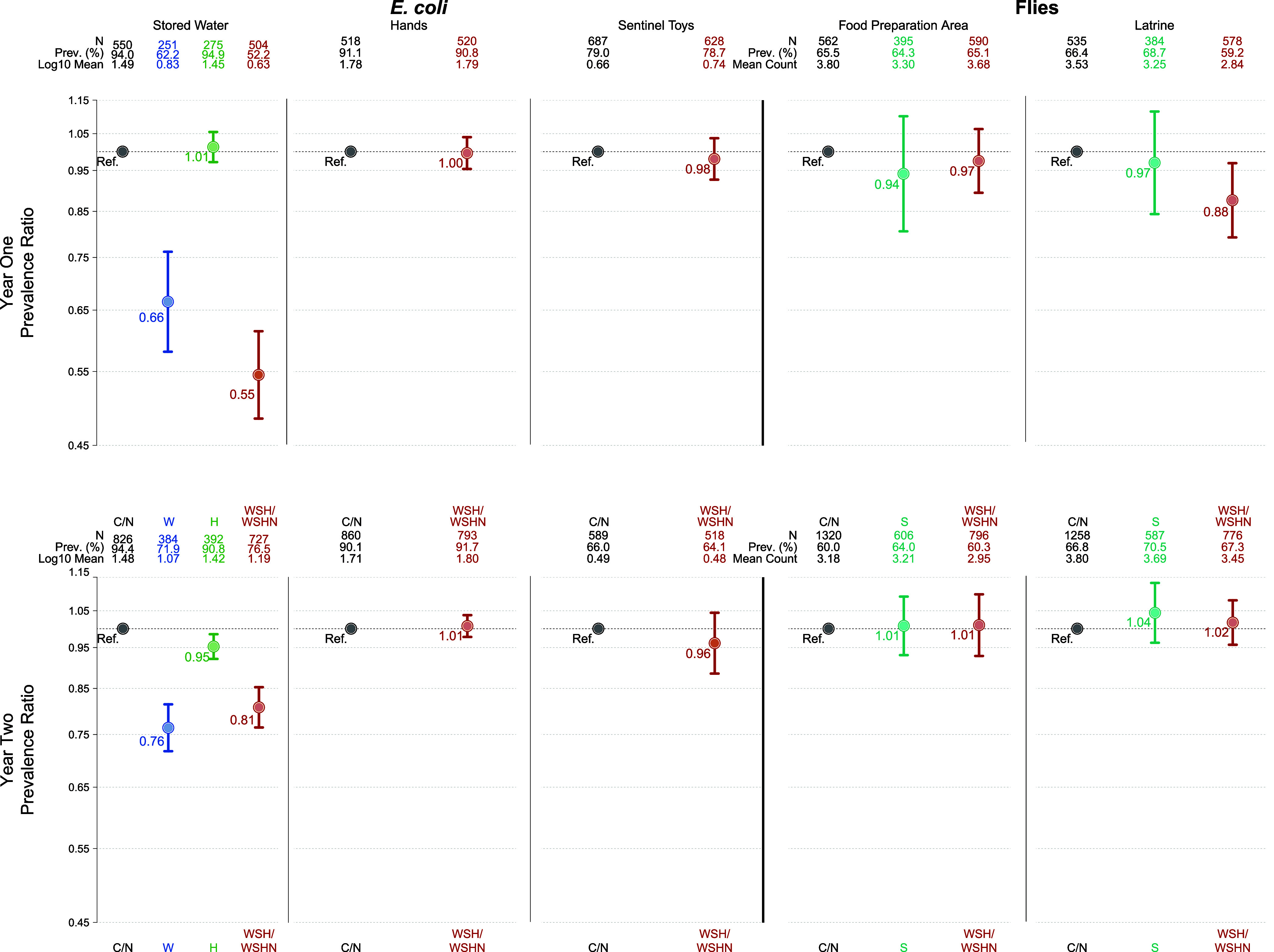
Intervention impacts on environmental contamination. Prevalence ratios of *E. coli* in stored water, on child hands, and on toys by intervention arm compared to the control group (C/N) (left); prevalence ratios of flies measured at food preparation areas and latrines by intervention arm compared to the control group (right).

Water treatment reduced the prevalence of *E. coli* in drinking water by 34% (prevalence ratio [PR]: 0.66, 95% confidence interval [CI] 0.58, 0.76) after 1 year of intervention exposure and by 24% (PR: 0.76, 95% CI: 0.72, 0.81) 2 years after interventions began (Figure 2 and Table S1). The combined WSH intervention showed similar effects on water quality to water treatment alone (45% reduction in *E. coli* at year 1; 19% reduction at year 2). Handwashing with soap slightly reduced the prevalence of *E. coli* contamination in stored drinking water at year 2 (PR: 0.95, 95% CI: 0.92, 0.99) but not at year 1; the handwashing intervention tended to reduce *E. coli* concentrations in stored water, but results were not significant (year 1 log_10_ CFU/100 mL difference: −0.05, year 1 95% CI: −0.22, 0.12; year 2 log_10_ CFU/100 mL difference: −0.07, year 2 95% CI: −0.19, 0.05). Differences in log-transformed *E. coli* concentrations across all intervention groups are reported in Table S1.

#### Child Hand and Toy Contamination

The combined WSH interventions did not affect the presence or levels of fecal indicator bacteria on child hands or toy balls at any time point (Figure 2 and Table S1). The combined WSH interventions reduced the prevalence of visible dirt on caregiver hands (PR averaged over both measurements: 0.86, 95% CI: 0.78, 0.95) and under caregiver fingernails (PR averaged over both measurements: 0.90, 95% CI: 0.85, 0.96) but did not affect visible dirt on child hands or underneath child fingernails (Table S3). These data suggest that handwashing frequency marginally increased in the WSH arms, but these increases were not sufficient to reduce *E. coli* contamination on hands.

#### Fly Prevalence and Densities

The combined WSH intervention reduced the prevalence and density of flies near the latrine at year 1 (PR: 0.88, 95% CI: 0.79, 0.97; −0.6 less flies counted, 95% CI: −1.2, 0.0) but not at year 2 (Figure 2 and Table S3). No interventions affected the prevalence or density of flies in the food preparation area. We also did not detect any differences in the prevalence of fly species between the study arms (Table S4).

### Animal Ownership

Animal ownership was high in our study population, with 90.2% (3777/4187) and 90.6% (3796/4192) of households owning any animal at enrollment and year 2, respectively.

By species, household poultry ownership was most common (enrollment, year 2 (%): 85.3, 84.7), followed by cattle (47.2, 51.4), dog (20.0, 14.9), goat (12.5, 16.4), and sheep (not measured, 5.56) ownership. Among animal-owning households, households owned an average of 9 animals (SD: 9) at enrollment and year 2. By species at enrollment and year 2, animal-owning households owned the greatest number of poultry (mean [SD]: 8 [8]), followed by cattle (2 [2]), goats (2 [2]), sheep (2 [2]), and dogs (2 [1]). Between enrollment and year 2, 15.1% (633/4192) of households changed ownership status for any type of animal. The greatest number of households changed cattle ownership status (30.4%), followed by poultry (23.0%), dogs (20.8%), and goats (19.4%); sheep ownership data was not collected at enrollment.

### Associations between Animal Ownership and Environmental Contamination

#### Water Quality

**Figure 3:**
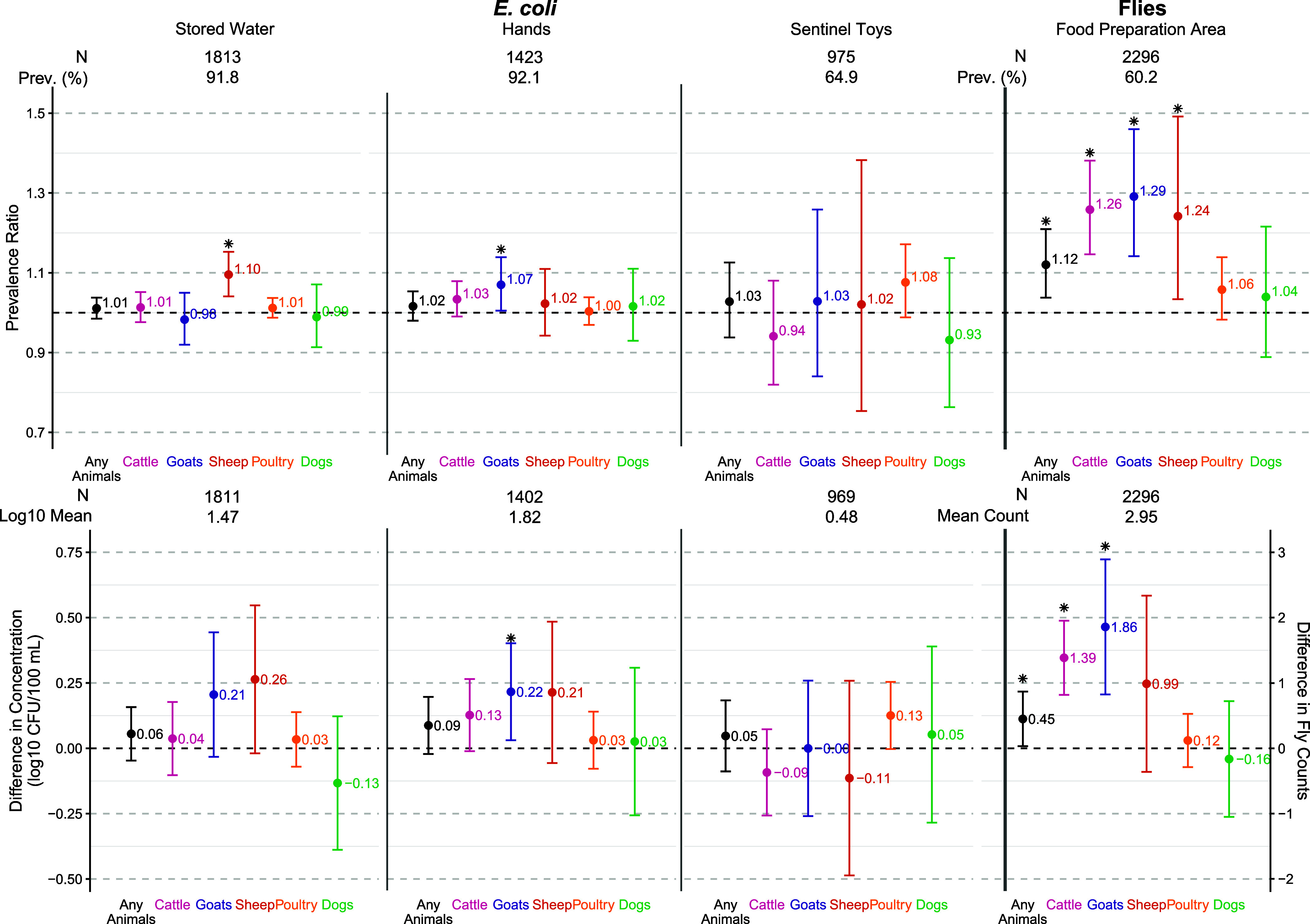
Associations between log_10_ number of animals owned by households and environmental contamination. Prevalence ratios (top) and differences in log_10_ concentrations (bottom) for *E. coli* in stored water, on child hands, and on toys for each additional log_10_ animal owned (left); prevalence ratios (top) and differences in fly counts for flies at food preparation areas for each additional log_10_ animal owned (right). Asterisks indicate significance at a significance level of 0.05.

Associations between animal ownership and *E. coli* prevalence and concentration in stored water were assessed among households without detectable free chlorine in their drinking water, as detectable chlorine in water samples could mask these relationships. For every additional log_10_ number of sheep owned by a household, the prevalence of *E. coli* in stored water was 10% greater (PR: 1.10, 95% CI: 1.04, 1.15) at year 2 (Figure 3). The prevalence of *E. coli* in water was also 6% (PR: 1.06, 95% CI: 1.02, 1.10) higher in sheep-owning households than in households without sheep (Figures S10 and S11). There were no other associations between household animal ownership and drinking water quality. There were even stronger associations between community-level sheep ownership and *E. coli* in water (PR: 1.16, 95% CI: 1.08, 1.26; 0.88 log_10_ CFU/100 mL increase, 95% CI: 0.28–1.47 for every additional log_10_ median number of sheep owned in the same village) (Figure S12). Higher numbers of goats, poultry, and any animal species owned in the community were also associated with higher *E. coli* concentrations in water (Figure S12). Specifically, *E. coli* concentrations in water were 0.52 (goats), 0.88 (poultry), and 0.35 (any animal species) log_10_ CFU/100 mL higher for every additional log_10_ median number of animals owned in the community for each animal category.

#### Child Hand and Toy Contamination

A 1 log_10_ increase in the number of goats owned by a household was associated with 7% (PR: 1.07, 95% CI: 1.01, 1.14) greater *E. coli* prevalence and 0.22 log_10_ CFU/100 mL (difference: 0.22, 95% CI: 0.09, 2.29) higher *E. coli* concentration on child hands (Figure 3). Higher TLU scores were associated with higher *E. coli* concentrations on child hands, though the result was only significant for TLU scores between 0.10 and 0.48 (log_10_ CFU/100 mL difference: 0.24, 95% CI: 0.03, 0.45; Figure S9). Binary household goat ownership was also associated with higher *E. coli* prevalence and concentration on hands (PR: 1.05, 95% CI: 1.01, 1.09; log_10_ CFU/100 mL difference: 0.17, 95% CI: 0.04, 0.30; Figures S10 and S11). Sensitivity analyses revealed potential associations between poultry and any animal ownership and higher *E. coli* concentration on hands, but there were no other associations between household animal ownership and hand contamination (Figures S10–S11). Community-level goat ownership showed slightly stronger positive associations with *E. coli* prevalence and concentration on child hands (PR: 1.16, 95% CI: 1.08, 1.25; log_10_ CFU/100 mL difference: 0.50, 95% CI: 0.04, 0.95) (Figure S12).

Household animal ownership was not associated with *E. coli* prevalence or concentration on sentinel toys (Figures 3 and S9–S11). An increase of 1 log_10_ village-median number of goats owned, however, was associated with a 34% higher prevalence of *E. coli* in toy ball rinses (PR: 1.34, 95% CI: 1.00, 1.80) (Figure S12).

#### Fly Prevalence and Densities

Increased numbers of ruminants (cattle, goats, sheep) and any animal species owned by households were associated with higher fly prevalence and densities (excluding sheep ownership) at food preparation areas (Figure 3). Higher TLU scores trended toward increased fly prevalence and densities, though the result was only significant for scores ≥1.5 (Figure S9). Similarly, binary cattle and goat ownership were associated with 11–18% greater fly prevalence and 0.69–1.28 more flies counted in food preparation areas (Figures S10 and S11). Community-level ownership of goats and any animal species was also associated with greater fly prevalence and densities in household food preparation areas (Figure S12).

### Associations between Animal Ownership and Child Health

**Figure 4:**
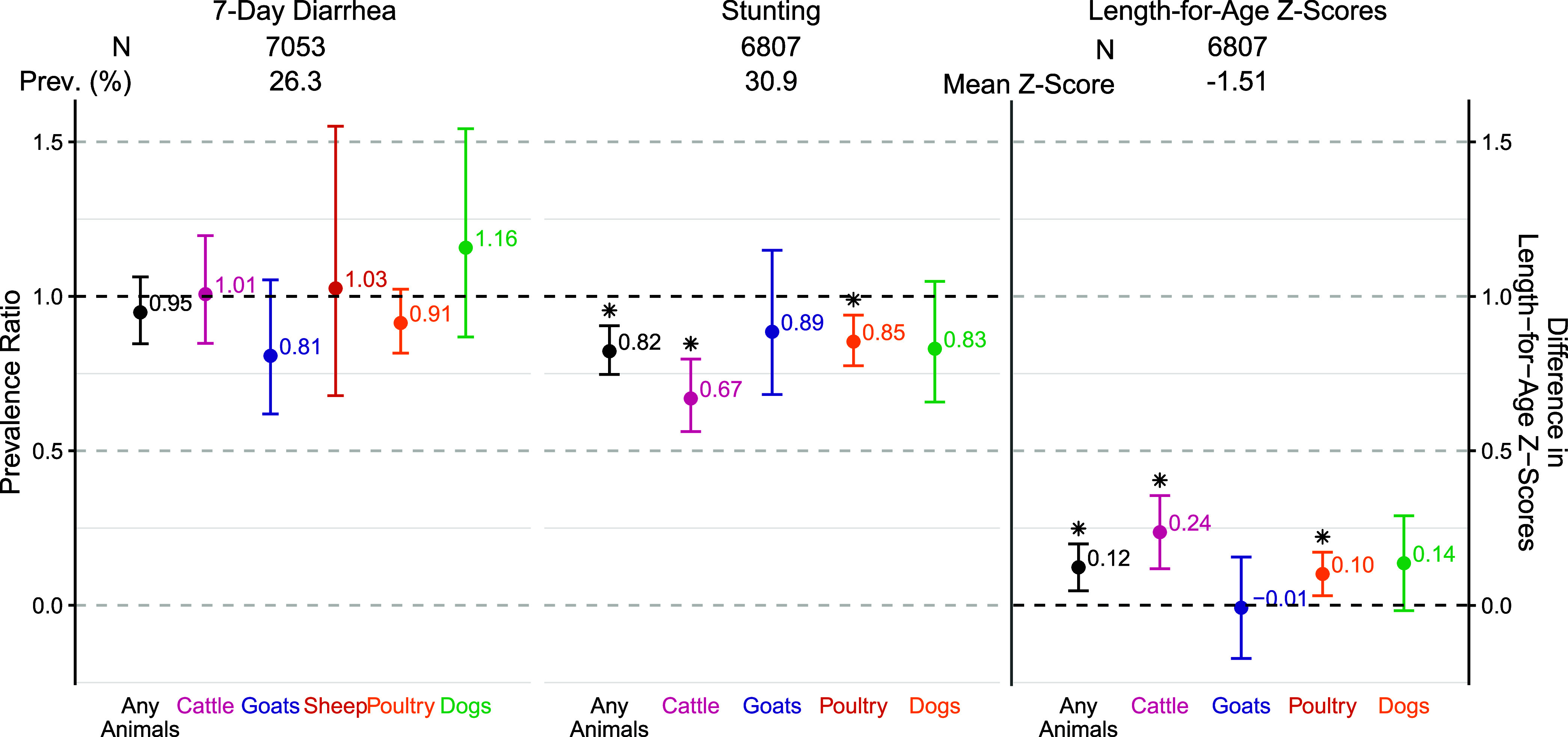
Associations between log_10_ number of animals owned by households and child health. Prevalence ratios for diarrhea and stunting for each additional log_10_ animal owned (left); differences in length-for-age *Z*-scores for each additional log_10_ animal owned (right). Asterisks indicate significance at a significance level of 0.05.

Diarrhea data were collected from 5795 children at year 2, and length-for-age data were collected from 6587 children. Overall, neither household nor community-level animal ownership of any species was associated with the prevalence of diarrhea in young children (Figures 4, S13–S15, and S18). However, a sensitivity analysis revealed that binary poultry ownership among households with extreme predicted probabilities excluded could be associated with a lower prevalence of diarrhea (Figure S15). Greater numbers of cattle, poultry, or any animal species owned by a household were associated with 0.10–0.24 higher length-for-age *Z*-scores and consequently 15–33% lower prevalence of stunting (PR: 0.67–0.85), among under-5 children (Figure 4). TLU scores ≥0.50 were also associated with higher length-for-age *Z*-scores and lower prevalence of stunting (Figure S13). The prevalence of stunting was 24% (PR: 0.76, 95% CI: 0.69, 0.85) lower in cattle-owning households compared to that in noncattle-owning households (Figures S14 and S15). Conversely, the prevalence of stunting was 20% (PR: 1.20, 95% CI: 1.03, 1.42) higher in households that changed from owning any animals to not owning animals between enrollment and year 2 (compared to staying the same status or changing to owning animals) (Figure S17). Community-level ownership of any animal species overall was associated with decreased stunting (PR: 0.83, 95% CI: 0.70–0.98) in young children (Figure S18).

### Discussion

We found limited impacts of WSH interventions, alone and in combination, on *E. coli* contamination in rural Kenyan households. Specifically, water treatment with chlorine reduced and handwashing marginally reduced the prevalence and concentration of *E. coli* in stored water, while none of the interventions impacted *E. coli* prevalence or concentrations on child hands or toy balls. Our findings confirm that drinking water chlorination is an effective method to reduce chlorine-susceptible contaminants in low-income settings.^13^ If adoption of chlorine had been higher in the parent trial than the roughly 40% observed at year 1 and 20% at year 2, the reductions in stored water *E. coli* contamination likely would have been larger.^61,62^ The effectiveness of the water intervention in improving water quality complements observed reductions in roundworm (*Ascaris lumbricoides*) infection prevalence among children receiving the water intervention in the trial.^46^*Ascaris* infection prevalence was reduced by 18–22% in the intervention arms that included a water treatment component (water, WSH, and WSHN arms) but not in other intervention arms. Further, water chlorination may be an important strategy for protecting household members from zoonotic enteropathogens, as sheep ownership was associated with increased *E. coli* contamination in drinking water. Similarly, previous work in the same study area found that 89% (40/45) of household stored water and 27% (12/45) of source water samples were contaminated with the ruminant-specific fecal marker, BacR.^37^ While most households (>68%) enrolled in the WASH Benefits Kenya trial accessed protected springs or wells as their primary drinking water source, roughly a quarter of households relied on unprotected water sources (e.g., unprotected springs, dug wells, surface water) that are prone to contamination by animal feces.^29,46^ Our findings are consistent with previous work in rural India that found that higher village sheep populations increased the odds of detecting higher *Cryptosporidium* spp. concentrations in ponds.^63^ Importantly, many child diarrheal disease episodes are attributable to chlorine-resistant organisms, including those carried by ruminant hosts (e.g., *Cryptosporidium* spp.).^64^ Chlorine might therefore need to be incorporated along with other strategies (e.g., filtration or source and distribution system improvements) to comprehensively improve water quality in low-income settings.^65^

A previous study in Tanzania found that despite heterogeneity in random hand rinse measurements, the level of fecal indicator bacteria on caregiver hands was the strongest predictor of the level of fecal indicator bacteria in stored drinking water.^8,16,17^ Our finding that the handwashing intervention marginally reduced *E. coli* concentration in water supports a link between hand contamination and stored drinking water quality; however, the magnitude of the improvement in water quality was small. Further trials that achieve high rates of handwashing with soap could be useful to better quantify the effect of increased frequency of handwashing with soap on drinking water quality.

The lack of effect on fecal indicator bacteria measured on hands and toys has several possible explanations, including inconsistent compliance with the targeted hygiene and sanitation behaviors, failure of these specific types of WSH interventions to reduce human fecal contamination on child hands and toys, or animal fecal contamination in the household environment.^29,32,33^ In addition, *E. coli* may be a poor proxy for fecal contamination, despite its frequent use as a fecal indicator bacteria.^66^ Our findings are consistent with the parallel WASH Benefits trial in Bangladesh, which also reported no reduction in child hand or toy *E. coli* contamination in the WSH intervention arms despite having higher uptake of the interventions.^67^ Notably, our post hoc analyses revealed that child hand contamination was higher in households owning greater numbers of goats. Similarly, ruminant fecal markers were prevalent in child hand rinse samples in urban and rural Bangladesh.^33,34^ Hand contamination can be an especially important risk factor for the transmission of zoonotic pathogens to young children, as hand- and object-mouthing is frequent in children from low-income settings.^49^

Flies are recognized vectors for human pathogens, and fly control programs have been found effective in reducing diarrheal illness.^68−71^ We observed marginal fly reductions at latrines from the combined WSH intervention and no fly reductions at food preparation areas. A trial evaluating community-led total sanitation in Mali detected a reduction in fly presence at latrines, while other trials in rural India and The Gambia found no reduction.^19,72,73^ Fly reductions in this trial may have been limited due to insufficient adherence to the sanitation intervention. Although household access to a latrine with an enumerator-observed slab or ventilation pipe remained higher than 78% among households in sanitation arms, the reported safe disposal of child feces into a latrine and observed placements of drop hole covers over latrines declined over the duration of the parent trial.^22^ Notably, the intervention was delivered at the compound rather than the community level (neighboring compounds to the study compound without a pregnant woman did not receive upgraded pit latrines with covers). The house fly (*Musca* domestica), which was the most prevalent species in our study site, has a flight range of up to 7 km and could travel daily between households.^74^ Additionally, the scoop provided in the sanitation intervention may have been insufficient for removing animal feces from household living areas. We found that animal ownership, particularly ruminant ownership, was associated with higher fly prevalence and densities near food preparation areas. The WASH Benefits trial in Bangladesh found that a higher concentration of *E. coli* on flies caught in the food preparation area was associated with greater *E. coli* contamination of food, though the presence of animal feces was not associated with *E. coli* levels on flies.^32^ Notably, *E. coli* was only detected in 50% of the sampled flies. Fly presence and densities are therefore imperfect proxies for fecal contamination transferred by flies, and future work may want to assess whether WSH interventions can reduce the prevalence and concentration of fecal contaminants on flies, even when total fly populations are not impacted. In this study, we found that village-level ownership of ruminants, particularly sheep and goats, was associated with fly presence near household food preparation areas, as well as *E. coli* contamination in household drinking water, on child hands, and on sentinel toys; future interventions for interrupting community-wide enteropathogen transmission could therefore be more effective than intervening at the individual household level.

Although animal ownership was associated with increased fecal contamination—albeit with relatively small differences in *E. coli* concentrations—in our study households, it did not appear to be a risk factor for under-5 child diarrhea or growth faltering. On the contrary, household cattle and poultry ownership were associated with higher length-for-age *Z*-scores and lower prevalence of stunting; the prevalence of stunting was also higher in households that owned any animals at enrollment but not at year 2, compared to households that did not change ownership status or obtained animals during the study. Livestock husbandry is often promoted as a means to improve livelihoods and incur nutritional benefits—through increased consumption of animal-sourced food—for low-income households.^75,76^ Our work is consistent with a growing body of evidence that owning domesticated animals may reduce stunting among young children by increasing the consumption of nutrient-rich animal-sourced foods, household income, and women’s empowerment.^38,77,78^ The true relationship between animal exposure and linear growth is likely dependent on specific animal husbandry practices within the household.^79^ In rural Ethiopia, for example, poultry ownership was associated with increased length-for-age *Z*-scores, but corralling poultry inside the home overnight was negatively associated with child growth.^38^ As food animal production is predicted to increase in LMICs and animal husbandry is often promoted to improve livelihoods and nutrition in low-income households, key behaviors affecting human exposure to animal fecal contamination under varying contexts (e.g., rural versus urban; domestic versus community) should be addressed in future research.^29,75,76^

### Limitations

There are some limitations in this study. First, fecal indicator bacteria can be a poor proxy for enteric pathogens and thus may exhibit limited associations with health outcomes in individual studies.^80^ Future evaluations should consider measuring specific human pathogens in water and in the environment to better understand how WSH interventions affect transmission pathways. Further, environmental sampling of *E. coli* cannot confirm whether *E. coli* originated from fecal sources, as some strains of *E. coli* can survive long time periods and potentially reproduce in natural (i.e., extraintestinal) environments.^66^ Second, we have been careful to interpret the intervention effects on environmental contamination alongside the relatively limited adherence to the interventions in the parent trial. Higher uptake of the interventions could have led to larger effects, but our reported results may be relevant for large-scale WSH programs that are unable to implement behavior change programs that are as intensive as those in efficacy studies. Third, while we adjusted for relevant covariates in the association between animal ownership and other outcomes, including intervention arm, there is potential for residual confounding in these analyses. Further, animal ownership is likely correlated with some covariates, such as household asset ownership as measures of socioeconomic status. We discovered and prevented positivity violations by excluding households with extreme predictive probabilities of animal ownership as sensitivity analyses for models with binary animal ownership as the exposures.

### Implications

Provision and promotion of chlorine for water treatment improved drinking water quality, but adoption of the water treatment intervention was much lower than expected by the end of the study. This emphasizes the difficulty of achieving sustained and consistent usage of household water treatment products with monthly, or less frequent, behavior promotion visits.^81,82^ We found no evidence that combining water, sanitation, and handwashing interventions led to larger reductions in fecal contamination in the household environment than single interventions, a finding consistent with no additive benefit on health outcomes measured in this trial or in other studies.^21,22,83−86^ Our results indicate that the intensive WSH interventions, as implemented in this study, did not reduce levels of fecal indicator bacteria on child hands or toys, while they slightly reduced fly presence near latrines and marginally improved visible hand cleanliness of caregivers. The failure of the interventions to reduce fecal contamination along important exposure pathways in the household suggests that WSH programs that aim to improve child health may need to consider interventions that cost more but also more comprehensively reduce fecal contamination in the household setting; our findings provide additional support for more effective WSH interventions.^29,30^

Improved animal feces management is one such consideration that has been called for in transformative WSH solutions.^31^ There are, however, two growing and opposing bodies of literature on the risks and benefits of animal husbandry on health in LMICs.^79^ Our findings demonstrated that domesticated animals were associated with *E. coli* prevalence and concentrations in the household environment but not with under-5 child diarrhea or increased stunting in rural Kenya. Our study therefore does not support restricting household animal ownership to prevent child diarrheal disease or stunting and cautions that future interventions may want to avoid promoting practices that increase the cost or burden of household animal rearing, which might jeopardize child growth benefits.

## Data Availability

This study was conducted using publicly available, deidentified data. All replication scripts and data are available on Open Science Framework at the following link: https://osf.io/eg2rc/.

## Supporting Information Available

Additional method details; additional results; directed acyclic graphs; WASH Benefits Kenya interventions;*E. coli* and total/fecal coliforms measured at 1- and 2-year assessments, combined and separately, interventions vs control;fly counts and dirt on hands/fingernails measured at 1- and 2-year assessments, combined and separately, interventions vs control; sensitivity and secondary (community-level) analyses for associations between animal ownership and environmental contamination and child health (PDF)

## Abbreviations

CFU: colony forming unit
CI: confidence interval
C/N: control/nutrition
EED: environmental enteric dysfunction
LMIC: low- and middle-income country
PR: prevalence ratio
RCT: randomized controlled trial
SD: standard deviation
WHO: World Health Organization
WSH: water, sanitation, and handwashing
WSHN: water, sanitation, handwashing, and nutrition

## References

[ref1] Wang H.; Naghavi M.; Allen C.; Barber R. M.; Bhutta Z. A.; Carter A.; Casey D. C.; Charlson F. J.; Chen A. Z.; Coates M. M.; Coggeshall M.; Dandona L.; Dicker D. J.; Erskine H. E.; Ferrari A. J.; Fitzmaurice C.; Foreman K.; Forouzanfar M. H.; Fraser M. S.; Fullman N.; Gething P. W.; Goldberg E. M.; Graetz N.; Haagsma J. A.; Hay S. I.; Huynh C.; Johnson C. O.; Kassebaum N. J.; Kinfu Y.; Kulikoff X. R.; Kutz M.; Kyu H. H.; Larson H. J.; Leung J.; Liang X.; Lim S. S.; Lind M.; Lozano R.; Marquez N.; Mensah G. A.; Mikesell J.; Mokdad A. H.; Mooney M. D.; Nguyen G.; Nsoesie E.; Pigott D. M.; Pinho C.; Roth G. A.; Salomon J. A.; Sandar L.; Silpakit N.; Sligar A.; Sorensen R. J. D.; Stanaway J.; Steiner C.; Teeple S.; Thomas B. A.; Troeger C.; VanderZanden A.; Vollset S. E.; Wanga V.; Whiteford H. A.; Wolock T.; Zoeckler L.; Abate K. H.; Abbafati C.; Abbas K. M.; Abd-Allah F.; Abera S. F.; Abreu D. M. X.; Abu-Raddad L. J.; Abyu G. Y.; Achoki T.; Adelekan A. L.; Ademi Z.; Adou A. K.; Adsuar J. C.; Afanvi K. A.; Afshin A.; Agardh E. E.; Agarwal A.; Agrawal A.; Kiadaliri A. A.; Ajala O. N.; Akanda A. S.; Akinyemi R. O.; Akinyemiju T. F.; Akseer N.; Lami F. H. A.; Alabed S.; Al-Aly Z.; Alam K.; Alam N. K. M.; Alasfoor D.; Aldhahri S. F.; Aldridge R. W.; Alegretti M. A.; Aleman A. V.; Alemu Z. A.; Alexander L. T.; Alhabib S.; Ali R.; Alkerwi A.; Alla F.; Allebeck P.; Al-Raddadi R.; Alsharif U.; Altirkawi K. A.; Martin E. A.; Alvis-Guzman N.; Amare A. T.; Amegah A. K.; Ameh E. A.; Amini H.; Ammar W.; Amrock S. M.; Andersen H. H.; Anderson B. O.; Anderson G. M.; Antonio C. A. T.; Aregay A. F.; Ärnlöv J.; Arsenijevic V. S. A.; Artaman A.; Asayesh H.; Asghar R. J.; Atique S.; Avokpaho E. F. G. A.; Awasthi A.; Azzopardi P.; Bacha U.; Badawi A.; Bahit M. C.; Balakrishnan K.; Banerjee A.; Barac A.; Barker-Collo S. L.; Bärnighausen T.; Barregard L.; Barrero L. H.; Basu A.; Basu S.; Bayou Y. T.; Bazargan-Hejazi S.; Beardsley J.; Bedi N.; Beghi E.; Belay H. A.; Bell B.; Bell M. L.; Bello A. K.; Bennett D. A.; Bensenor I. M.; Berhane A.; Bernabé E.; Betsu B. D.; Beyene A. S.; Bhala N.; Bhalla A.; Biadgilign S.; Bikbov B.; Abdulhak A. A. B.; Biroscak B. J.; Biryukov S.; Bjertness E.; Blore J. D.; Blosser C. D.; Bohensky M. A.; Borschmann R.; Bose D.; Bourne R. R. A.; Brainin M.; Brayne C. E. G.; Brazinova A.; Breitborde N. J. K.; Brenner H.; Brewer J. D.; Brown A.; Brown J.; Brugha T. S.; Buckle G. C.; Butt Z. A.; Calabria B.; Campos-Nonato I. R.; Campuzano J. C.; Carapetis J. R.; Cárdenas R.; Carpenter D. O.; Carrero J. J.; Castañeda-Orjuela C. A.; Rivas J. C.; Catalá-López F.; Cavalleri F.; Cercy K.; Cerda J.; Chen W.; Chew A.; Chiang P. P.-C.; Chibalabala M.; Chibueze C. E.; Chimed-Ochir O.; Chisumpa V. H.; Choi J.-Y. J.; Chowdhury R.; Christensen H.; Christopher D. J.; Ciobanu L. G.; Cirillo M.; Cohen A. J.; Colistro V.; Colomar M.; Colquhoun S. M.; Cooper C.; Cooper L. T.; Cortinovis M.; Cowie B. C.; Crump J. A.; Damsere-Derry J.; Danawi H.; Dandona R.; Daoud F.; Darby S. C.; Dargan P. I.; das Neves J.; Davey G.; Davis A. C.; Davitoiu D. V.; de Castro E. F.; de Jager P.; Leo D. D.; Degenhardt L.; Dellavalle R. P.; Deribe K.; Deribew A.; Dharmaratne S. D.; Dhillon P. K.; Diaz-Torné C.; Ding E. L.; dos Santos K. P. B.; Dossou E.; Driscoll T. R.; Duan L.; Dubey M.; Duncan B. B.; Ellenbogen R. G.; Ellingsen C. L.; Elyazar I.; Endries A. Y.; Ermakov S. P.; Eshrati B.; Esteghamati A.; Estep K.; Faghmous I. D. A.; Fahimi S.; Faraon E. J. A.; Farid T. A.; Farinha C. S. e S.; Faro A.; Farvid M. S.; Farzadfar F.; Feigin V. L.; Fereshtehnejad S.-M.; Fernandes J. G.; Fernandes J. C.; Fischer F.; Fitchett J. R. A.; Flaxman A.; Foigt N.; Fowkes F. G. R.; Franca E. B.; Franklin R. C.; Friedman J.; Frostad J.; Fürst T.; Futran N. D.; Gall S. L.; Gambashidze K.; Gamkrelidze A.; Ganguly P.; Gankpé F. G.; Gebre T.; Gebrehiwot T. T.; Gebremedhin A. T.; Gebru A. A.; Geleijnse J. M.; Gessner B. D.; Ghoshal A. G.; Gibney K. B.; Gillum R. F.; Gilmour S.; Giref A. Z.; Giroud M.; Gishu M. D.; Giussani G.; Glaser E.; Godwin W. W.; Gomez-Dantes H.; Gona P.; Goodridge A.; Gopalani S. V.; Gosselin R. A.; Gotay C. C.; Goto A.; Gouda H. N.; Greaves F.; Gugnani H. C.; Gupta R.; Gupta R.; Gupta V.; Gutiérrez R. A.; Hafezi-Nejad N.; Haile D.; Hailu A. D.; Hailu G. B.; Halasa Y. A.; Hamadeh R. R.; Hamidi S.; Hancock J.; Handal A. J.; Hankey G. J.; Hao Y.; Harb H. L.; Harikrishnan S.; Haro J. M.; Havmoeller R.; Heckbert S. R.; Heredia-Pi I. B.; Heydarpour P.; Hilderink H. B. M.; Hoek H. W.; Hogg R. S.; Horino M.; Horita N.; Hosgood H. D.; Hotez P. J.; Hoy D. G.; Hsairi M.; Htet A. S.; Htike M. M. T.; Hu G.; Huang C.; Huang H.; Huiart L.; Husseini A.; Huybrechts I.; Huynh G.; Iburg K. M.; Innos K.; Inoue M.; Iyer V. J.; Jacobs T. A.; Jacobsen K. H.; Jahanmehr N.; Jakovljevic M. B.; James P.; Javanbakht M.; Jayaraman S. P.; Jayatilleke A. U.; Jeemon P.; Jensen P. N.; Jha V.; Jiang G.; Jiang Y.; Jibat T.; Jimenez-Corona A.; Jonas J. B.; Joshi T. K.; Kabir Z.; Kamal R.; Kan H.; Kant S.; Karch A.; Karema C. K.; Karimkhani C.; Karletsos D.; Karthikeyan G.; Kasaeian A.; Katibeh M.; Kaul A.; Kawakami N.; Kayibanda J. F.; Keiyoro P. N.; Kemmer L.; Kemp A. H.; Kengne A. P.; Keren A.; Kereselidze M.; Kesavachandran C. N.; Khader Y. S.; Khalil I. A.; Khan A. R.; Khan E. A.; Khang Y.-H.; Khera S.; Khoja T. A. M.; Kieling C.; Kim D.; Kim Y. J.; Kissela B. M.; Kissoon N.; Knibbs L. D.; Knudsen A. K.; Kokubo Y.; Kolte D.; Kopec J. A.; Kosen S.; Koul P. A.; Koyanagi A.; Krog N. H.; Defo B. K.; Bicer B. K.; Kudom A. A.; Kuipers E. J.; Kulkarni V. S.; Kumar G. A.; Kwan G. F.; Lal A.; Lal D. K.; Lalloo R.; Lallukka T.; Lam H.; Lam J. O.; Langan S. M.; Lansingh V. C.; Larsson A.; Laryea D. O.; Latif A. A.; Lawrynowicz A. E. B.; Leigh J.; Levi M.; Li Y.; Lindsay M. P.; Lipshultz S. E.; Liu P. Y.; Liu S.; Liu Y.; Lo L.-T.; Logroscino G.; Lotufo P. A.; Lucas R. M.; Lunevicius R.; Lyons R. A.; Ma S.; Machado V. M. P.; Mackay M. T.; MacLachlan J. H.; Razek H. M. A. E.; Magdy M.; Razek A. E.; Majdan M.; Majeed A.; Malekzadeh R.; Manamo W. A. A.; Mandisarisa J.; Mangalam S.; Mapoma C. C.; Marcenes W.; Margolis D. J.; Martin G. R.; Martinez-Raga J.; Marzan M. B.; Masiye F.; Mason-Jones A. J.; Massano J.; Matzopoulos R.; Mayosi B. M.; McGarvey S. T.; McGrath J. J.; McKee M.; McMahon B. J.; Meaney P. A.; Mehari A.; Mehndiratta M. M.; Mejia-Rodriguez F.; Mekonnen A. B.; Melaku Y. A.; Memiah P.; Memish Z. A.; Mendoza W.; Meretoja A.; Meretoja T. J.; Mhimbira F. A.; Micha R.; Millear A.; Miller T. R.; Mirarefin M.; Misganaw A.; Mock C. N.; Mohammad K. A.; Mohammadi A.; Mohammed S.; Mohan V.; Mola G. L. D.; Monasta L.; Hernandez J. C. M.; Montero P.; Montico M.; Montine T. J.; Moradi-Lakeh M.; Morawska L.; Morgan K.; Mori R.; Mozaffarian D.; Mueller U. O.; Murthy G. V. S.; Murthy S.; Musa K. I.; Nachega J. B.; Nagel G.; Naidoo K. S.; Naik N.; Naldi L.; Nangia V.; Nash D.; Nejjari C.; Neupane S.; Newton C. R.; Newton J. N.; Ng M.; Ngalesoni F. N.; de Dieu Ngirabega J.; Nguyen Q. L.; Nisar M. I.; Pete P. M. N.; Nomura M.; Norheim O. F.; Norman P. E.; Norrving B.; Nyakarahuka L.; Ogbo F. A.; Ohkubo T.; Ojelabi F. A.; Olivares P. R.; Olusanya B. O.; Olusanya J. O.; Opio J. N.; Oren E.; Ortiz A.; Osman M.; Ota E.; Ozdemir R.; PA M.; Pain A.; Pandian J. D.; Pant P. R.; Papachristou C.; Park E.-K.; Park J.-H.; Parry C. D.; Parsaeian M.; Caicedo A. J. P.; Patten S. B.; Patton G. C.; Paul V. K.; Pearce N.; Pedro J. M.; Stokic L. P.; Pereira D. M.; Perico N.; Pesudovs K.; Petzold M.; Phillips M. R.; Piel F. B.; Pillay J. D.; Plass D.; Platts-Mills J. A.; Polinder S.; Pope C. A.; Popova S.; Poulton R. G.; Pourmalek F.; Prabhakaran D.; Qorbani M.; Quame-Amaglo J.; Quistberg D. A.; Rafay A.; Rahimi K.; Rahimi-Movaghar V.; Rahman M.; Rahman M. H. U.; Rahman S. U.; Rai R. K.; Rajavi Z.; Rajsic S.; Raju M.; Rakovac I.; Rana S. M.; Ranabhat C. L.; Rangaswamy T.; Rao P.; Rao S. R.; Refaat A. H.; Rehm J.; Reitsma M. B.; Remuzzi G.; Resnikoff S.; Ribeiro A. L.; Ricci S.; Blancas M. J. R.; Roberts B.; Roca A.; Rojas-Rueda D.; Ronfani L.; Roshandel G.; Rothenbacher D.; Roy A.; Roy N. K.; Ruhago G. M.; Sagar R.; Saha S.; Sahathevan R.; Saleh M. M.; Sanabria J. R.; Sanchez-Niño M. D.; Sanchez-Riera L.; Santos I. S.; Sarmiento-Suarez R.; Sartorius B.; Satpathy M.; Savic M.; Sawhney M.; Schaub M. P.; Schmidt M. I.; Schneider I. J. C.; Schöttker B.; Schutte A. E.; Schwebel D. C.; Seedat S.; Sepanlou S. G.; Servan-Mori E. E.; Shackelford K. A.; Shaddick G.; Shaheen A.; Shahraz S.; Shaikh M. A.; Shakh-Nazarova M.; Sharma R.; She J.; Sheikhbahaei S.; Shen J.; Shen Z.; Shepard D. S.; Sheth K. N.; Shetty B. P.; Shi P.; Shibuya K.; Shin M.-J.; Shiri R.; Shiue I.; Shrime M. G.; Sigfusdottir I. D.; Silberberg D. H.; Silva D. A. S.; Silveira D. G. A.; Silverberg J. I.; Simard E. P.; Singh A.; Singh G. M.; Singh J. A.; Singh O. P.; Singh P. K.; Singh V.; Soneji S.; Søreide K.; Soriano J. B.; Sposato L. A.; Sreeramareddy C. T.; Stathopoulou V.; Stein D. J.; Stein M. B.; Stranges S.; Stroumpoulis K.; Sunguya B. F.; Sur P.; Swaminathan S.; Sykes B. L.; Szoeke C. E. I.; Tabarés-Seisdedos R.; Tabb K. M.; Takahashi K.; Takala J. S.; Talongwa R. T.; Tandon N.; Tavakkoli M.; Taye B.; Taylor H. R.; Ao B. J. T.; Tedla B. A.; Tefera W. M.; Have M. T.; Terkawi A. S.; Tesfay F. H.; Tessema G. A.; Thomson A. J.; Thorne-Lyman A. L.; Thrift A. G.; Thurston G. D.; Tillmann T.; Tirschwell D. L.; Tonelli M.; Topor-Madry R.; Topouzis F.; Towbin J. A.; Traebert J.; Tran B. X.; Truelsen T.; Trujillo U.; Tura A. K.; Tuzcu E. M.; Uchendu U. S.; Ukwaja K. N.; Undurraga E. A.; Uthman O. A.; Dingenen R. V.; van Donkelaar A.; Vasankari T.; Vasconcelos A. M. N.; Venketasubramanian N.; Vidavalur R.; Vijayakumar L.; Villalpando S.; Violante F. S.; Vlassov V. V.; Wagner J. A.; Wagner G. R.; Wallin M. T.; Wang L.; Watkins D. A.; Weichenthal S.; Weiderpass E.; Weintraub R. G.; Werdecker A.; Westerman R.; White R. A.; Wijeratne T.; Wilkinson J. D.; Williams H. C.; Wiysonge C. S.; Woldeyohannes S. M.; Wolfe C. D. A.; Won S.; Wong J. Q.; Woolf A. D.; Xavier D.; Xiao Q.; Xu G.; Yakob B.; Yalew A. Z.; Yan L. L.; Yano Y.; Yaseri M.; Ye P.; Yebyo H. G.; Yip P.; Yirsaw B. D.; Yonemoto N.; Yonga G.; Younis M. Z.; Yu S.; Zaidi Z.; Zaki M. E. S.; Zannad F.; Zavala D. E.; Zeeb H.; Zeleke B. M.; Zhang H.; Zodpey S.; Zonies D.; Zuhlke L. J.; Vos T.; Lopez A. D.; Murray C. J. L. Global, Regional, and National Life Expectancy, All-Cause Mortality, and Cause-Specific Mortality for 249 Causes of Death, 1980–2015: A Systematic Analysis for the Global Burden of Disease Study 2015. Lancet 2016, 388 (10053), 1459–1544. 10.1016/S0140-6736(16)31012-1. 27733281 PMC5388903

[ref2] Richard S. A.; Black R. E.; Gilman R. H.; Guerrant R. L.; Kang G.; Lanata C. F.; Mølbak K.; Rasmussen Z. A.; Sack R. B.; Valentiner-Branth P.; Checkley W. Diarrhea in Early Childhood: Short-Term Association With Weight and Long-Term Association With Length. Am. J. Epidemiol. 2013, 178 (7), 1129–1138. 10.1093/aje/kwt094. 23966558 PMC3783094

[ref3] The UNICEF/WHO/WB Joint Child Malnutrition Estimates (JME) Group Released New Data, 2022. https://www.who.int/news/item/31-03-2020-unicef-who-wb-jme-group-new-data. accessed May 27, 2022.

[ref4] Take Action for the Sustainable Development Goals—United Nations Sustainable Development, 2022. https://www.un.org/sustainabledevelopment/sustainable-development-goals/. accessed May 27, 2022.

[ref5] Esrey S. A.; Feachem R. G.; Hughes J. M. Interventions for the Control of Diarrhoeal Diseases among Young Children: Improving Water Supplies and Excreta Disposal Facilities. Bull. W. H. O. 1985, 63 (4), 757–772. 3878742 PMC2536385

[ref6] Navab-Daneshmand T.; Friedrich M. N. D.; Gächter M.; Montealegre M. C.; Mlambo L. S.; Nhiwatiwa T.; Mosler H.-J.; Julian T. R. *Escherichia Coli* Contamination across Multiple Environmental Compartments (Soil, Hands, Drinking Water, and Handwashing Water) in Urban Harare: Correlations and Risk Factors. Am. J. Trop. Med. Hyg. 2018, 98 (3), 803–813. 10.4269/ajtmh.17-0521. 29363444 PMC5930891

[ref7] Exum N. G.; Olórtegui M. P.; Yori P. P.; Davis M. F.; Heaney C. D.; Kosek M.; Schwab K. J. Floors and Toilets: Association of Floors and Sanitation Practices with Fecal Contamination in Peruvian Amazon Peri-Urban Households. Environ. Sci. Technol. 2016, 50 (14), 7373–7381. 10.1021/acs.est.6b01283. 27338564 PMC6400218

[ref8] Pickering A. J.; Davis J.; Walters S. P.; Horak H. M.; Keymer D. P.; Mushi D.; Strickfaden R.; Chynoweth J. S.; Liu J.; Blum A.; Rogers K.; Boehm A. B. Hands, Water, and Health: Fecal Contamination in Tanzanian Communities with Improved, Non-Networked Water Supplies. Environ. Sci. Technol. 2010, 44 (9), 3267–3272. 10.1021/es903524m. 20222746

[ref9] Pickering A. J.; Julian T. R.; Marks S. J.; Mattioli M. C.; Boehm A. B.; Schwab K. J.; Davis J. Fecal Contamination and Diarrheal Pathogens on Surfaces and in Soils among Tanzanian Households with and without Improved Sanitation. Environ. Sci. Technol. 2012, 46 (11), 5736–5743. 10.1021/es300022c. 22545817

[ref10] Julian T. R. Environmental Transmission of Diarrheal Pathogens in Low and Middle Income Countries. Environ. Sci.: Processes Impacts 2016, 18 (8), 944–955. 10.1039/C6EM00222F. 27384220

[ref11] Clasen T. F.; Bostoen K.; Schmidt W.-P.; Boisson S.; Fung I. C.-H.; Jenkins M. W.; Scott B.; Sugden S.; Cairncross S. Interventions to Improve Disposal of Human Excreta for Preventing Diarrhoea. Cochrane Database Syst. Rev. 2010, No (6), CD007180 10.1002/14651858.CD007180.pub2. PMC6532559 20556776

[ref12] Clasen T. F.; Alexander K. T.; Sinclair D.; Boisson S.; Peletz R.; Chang H. H.; Majorin F.; Cairncross S. Interventions to Improve Water Quality for Preventing Diarrhoea. Cochrane Database Syst. Rev. 2015, 2015 (10), CD004794 10.1002/14651858.CD004794.pub3. 26488938 PMC4625648

[ref13] Arnold B. F.; Colford J. M. Treating Water with Chlorine at Point-of-Use to Improve Water Quality and Reduce Child Diarrhea in Developing Countries: A Systematic Review and Meta-Analysis. Am. J. Trop. Med. Hyg. 2007, 76 (2), 354–364. 10.4269/ajtmh.2007.76.354. 17297049

[ref14] Greene L. E.; Freeman M. C.; Akoko D.; Saboori S.; Moe C.; Rheingans R. Impact of a School-Based Hygiene Promotion and Sanitation Intervention on Pupil Hand Contamination in Western Kenya: A Cluster Randomized Trial. Am. J. Trop. Med. Hyg. 2012, 87 (3), 385–393. 10.4269/ajtmh.2012.11-0633. 22802437 PMC3435337

[ref15] Luby S. P.; Agboatwalla M.; Billhimer W.; Hoekstra R. M. Field Trial of a Low Cost Method to Evaluate Hand Cleanliness. Trop. Med. Int. Health 2007, 12 (6), 765–771. 10.1111/j.1365-3156.2007.01847.x. 17550474

[ref16] Pickering A. J.; Julian T. R.; Mamuya S.; Boehm A. B.; Davis J. Bacterial Hand Contamination among Tanzanian Mothers Varies Temporally and Following Household Activities. Trop. Med. Int. Health 2011, 16 (2), 233–239. 10.1111/j.1365-3156.2010.02677.x. 21091858

[ref17] Ram P. K.; Jahid I.; Halder A. K.; Nygren B.; Islam M. S.; Granger S. P.; Molyneaux J. W.; Luby S. P. Variability in Hand Contamination Based on Serial Measurements: Implications for Assessment of Hand-Cleansing Behavior and Disease Risk. Am. J. Trop. Med. Hyg. 2011, 84 (4), 510–516. 10.4269/ajtmh.2011.10-0299. 21460002 PMC3062441

[ref18] Sclar G. D.; Penakalapati G.; Amato H. K.; Garn J. V.; Alexander K.; Freeman M. C.; Boisson S.; Medlicott K. O.; Clasen T. Assessing the Impact of Sanitation on Indicators of Fecal Exposure along Principal Transmission Pathways: A Systematic Review. Int. J. Hyg. Environ. Health 2016, 219 (8), 709–723. 10.1016/j.ijheh.2016.09.021. 27720133

[ref19] Clasen T.; Boisson S.; Routray P.; Torondel B.; Bell M.; Cumming O.; Ensink J.; Freeman M.; Jenkins M.; Odagiri M.; Ray S.; Sinha A.; Suar M.; Schmidt W.-P. Effectiveness of a Rural Sanitation Programme on Diarrhoea, Soil-Transmitted Helminth Infection, and Child Malnutrition in Odisha, India: A Cluster-Randomised Trial. Lancet Global Health 2014, 2 (11), e645–653. 10.1016/S2214-109X(14)70307-9. 25442689

[ref20] Humphrey J. H.; Mbuya M. N. N.; Ntozini R.; Moulton L. H.; Stoltzfus R. J.; Tavengwa N. V.; Mutasa K.; Majo F.; Mutasa B.; Mangwadu G.; Chasokela C. M.; Chigumira A.; Chasekwa B.; Smith L. E.; Tielsch J. M.; Jones A. D.; Manges A. R.; Maluccio J. A.; Prendergast A. J.; Humphrey J. H.; Jones A. D.; Manges A.; Mangwadu G.; Maluccio J. A.; Mbuya M. N. N.; Moulton L. H.; Ntozini R.; Prendergast A. J.; Stoltzfus R. J.; Tielsch J. M.; Chasokela C.; Chigumira A.; Heylar W.; Hwena P.; Kembo G.; Majo F. D.; Mutasa B.; Mutasa K.; Rambanepasi P.; Sauramba V.; Tavengwa N. V.; Van Der Keilen F.; Zambezi C.; Chidhanguro D.; Chigodora D.; Chipanga J. F.; Gerema G.; Magara T.; Mandava M.; Mavhudzi T.; Mazhanga C.; Muzaradope G.; Mwapaura M. T.; Phiri S.; Tengende A.; Banda C.; Chasekwa B.; Chidamba L.; Chidawanyika T.; Chikwindi E.; Chingaona L. K.; Chiorera C. K.; Dandadzi A.; Govha M.; Gumbo H.; Gwanzura K. T.; Kasaru S.; Makasi R.; Matsika A. M.; Maunze D.; Mazarura E.; Mpofu E.; Mushonga J.; Mushore T. E.; Muzira T.; Nembaware N.; Nkiwane S.; Nyamwino P.; Rukobo S. D.; Runodamoto T.; Seremwe S.; Simango P.; Tome J.; Tsenesa B.; Amadu U.; Bangira B.; Chiveza D.; Hove P.; Jombe H. A.; Kujenga D.; Madhuyu L.; Makoni P. M.; Maramba N.; Maregere B.; Marumani E.; Masakadze E.; Mazula P.; Munyanyi C.; Musanhu G.; Mushanawani R. C.; Mutsando S.; Nazare F.; Nyarambi M.; Nzuda W.; Sigauke T.; Solomon M.; Tavengwa T.; Biri F.; Chafanza M.; Chaitezvi C.; Chauke T.; Chidzomba C.; Dadirai T.; Fundira C.; Gambiza A. C.; Godzongere T.; Kuona M.; Mafuratidze T.; Mapurisa I.; Mashedze T.; Moyo N.; Musariri C.; Mushambadope M.; Mutsonziwa T. R.; Muzondo A.; Mwareka R.; Nyamupfukudza J.; Saidi B.; Sakuhwehwe T.; Sikalima G.; Tembe J.; Chekera T. E.; Chihombe O.; Chikombingo M.; Chirinda T.; Chivizhe A.; Hove R.; Kufa R.; Machikopa T. F.; Mandaza W.; Mandongwe L.; Manhiyo F.; Manyaga E.; Mapuranga P.; Matimba F. S.; Matonhodze P.; Mhuri S.; Mike J.; Ncube B.; Nderecha W. T. S.; Noah M.; Nyamadzawo C.; Penda J.; Saidi A.; Shonhayi S.; Simon C.; Tichagwa M.; Chamakono R.; Chauke A.; Gatsi A. F.; Hwena B.; Jawi H.; Kaisa B.; Kamutanho S.; Kaswa T.; Kayeruza P.; Lunga J.; Magogo N.; Manyeruke D.; Mazani P.; Mhuriyengwe F.; Mlambo F.; Moyo S.; Mpofu T.; Mugava M.; Mukungwa Y.; Muroyiwa F.; Mushonga E.; Nyekete S.; Rinashe T.; Sibanda K.; Chemhuru M.; Chikunya J.; Chikwavaire V. F.; Chikwiriro C.; Chimusoro A.; Chinyama J.; Gwinji G.; Hoko-Sibanda N.; Kandawasvika R.; Madzimure T.; Maponga B.; Mapuranga A.; Marembo J.; Matsunge L.; Maunga S.; Muchekeza M.; Muti M.; Nyamana M.; Azhuda E.; Bhoroma U.; Biriyadi A.; Chafota E.; Chakwizira A.; Chamhamiwa A.; Champion T.; Chazuza S.; Chikwira B.; Chingozho C.; Chitabwa A.; Dhurumba A.; Furidzirai A.; Gandanga A.; Gukuta C.; Macheche B.; Marihwi B.; Masike B.; Mutangandura E.; Mutodza B.; Mutsindikwa A.; Mwale A.; Ndhlovu R.; Nduna N.; Nyamandi C.; Ruvata E.; Sithole B.; Urayai R.; Vengesa B.; Zorounye M.; Bamule M.; Bande M.; Chahuruva K.; Chidumba L.; Chigove Z.; Chiguri K.; Chikuni S.; Chikwanda R.; Chimbi T.; Chingozho M.; Chinhamo O.; Chinokuramba R.; Chinyoka C.; Chipenzi X.; Chipute R.; Chiribhani G.; Chitsinga M.; Chiwanga C.; Chiza A.; Chombe F.; Denhere M.; Dhamba E.; Dhamba M.; Dube J.; Dzimbanhete F.; Dzingai G.; Fusira S.; Gonese M.; Gota J.; Gumure K.; Gwaidza P.; Gwangwava M.; Gwara W.; Gwauya M.; Gwiba M.; Hamauswa J.; Hlasera S.; Hlukani E.; Hotera J.; Jakwa L.; Jangara G.; Janyure M.; Jari C.; Juru D.; Kapuma T.; Konzai P.; Mabhodha M.; Maburutse S.; Macheka C.; Machigaya T.; Machingauta F.; Machokoto E.; Madhumba E.; Madziise L.; Madziva C.; Madzivire M.; Mafukise M.; Maganga M.; Maganga S.; Mageja E.; Mahanya M.; Mahaso E.; Mahleka S.; Makanhiwa P.; Makarudze M.; Makeche C.; Makopa N.; Makumbe R.; Mandire M.; Mandiyanike E.; Mangena E.; Mangiro F.; Mangwadu A.; Mangwengwe T.; Manhidza J.; Manhovo F.; Manono I.; Mapako S.; Mapfumo E.; Mapfumo T.; Mapuka J.; Masama D.; Masenge G.; Mashasha M.; Mashivire V.; Matunhu M.; Mavhoro P.; Mawuka G.; Mazango I.; Mazhata N.; Mazuva D.; Mazuva M.; Mbinda F.; Mborera J.; Mfiri U.; Mhandu F.; Mhike C.; Mhike T.; Mhuka A.; Midzi J.; Moyo S.; Mpundu M.; Msekiwa N.; Msindo D.; Mtisi C.; Muchemwa G.; Mujere N.; Mukaro E.; Muketiwa K.; Mungoi S.; Munzava E.; Muoki R.; Mupura H.; Murerwa E.; Murisi C.; Muroyiwa L.; Muruvi M.; Musemwa N.; Mushure C.; Mutero J.; Mutero P.; Mutumbu P.; Mutya C.; Muzanango L.; Muzembi M.; Muzungunye D.; Mwazha V.; Ncube T.; Ndava T.; Ndlovu N.; Nehowa P.; Ngara D.; Nguruve L.; Nhigo P.; Nkiwane S.; Nyanyai L.; Nzombe J.; Office E.; Paul B.; Pavari S.; Ranganai S.; Ratisai S.; Rugara M.; Rusere P.; Sakala J.; Sango P.; Shava S.; Shekede M.; Shizha C.; Sibanda T.; Tapambwa N.; Tembo J.; Tinago N.; Tinago V.; Toindepi T.; Tovigepi J.; Tuhwe M.; Tumbo K.; Zaranyika T.; Zaru T.; Zimidzi K.; Zindo M.; Zindonda M.; Zinhumwe N.; Zishiri L.; Ziyambi E.; Zvinowanda J.; Bepete E.; Chiwira C.; Chuma N.; Fari A.; Gavi S.; Gunha V.; Hakunandava F.; Huku C.; Hungwe G.; Maduke G.; Manyewe E.; Mapfumo T.; Marufu I.; Mashiri C.; Mazenge S.; Mbinda E.; Mhuri A.; Muguti C.; Munemo L.; Musindo L.; Ngada L.; Nyembe D.; Taruvinga R.; Tobaiwa E.; Banda S.; Chaipa J.; Chakaza P.; Chandigere M.; Changunduma A.; Chibi C.; Chidyagwai O.; Chidza E.; Chigatse N.; Chikoto L.; Chingware V.; Chinhamo J.; Chinhoro M.; Chiripamberi A.; Chitavati E.; Chitiga R.; Chivanga N.; Chivese T.; Chizema F.; Dera S.; Dhliwayo A.; Dhononga P.; Dimingo E.; Dziyani M.; Fambi T.; Gambagamba L.; Gandiyari S.; Gomo C.; Gore S.; Gundani J.; Gundani R.; Gwarima L.; Gwaringa C.; Gwenya S.; Hamilton R.; Hlabano A.; Hofisi E.; Hofisi F.; Hungwe S.; Hwacha S.; Hwara A.; Jogwe R.; Kanikani A.; Kuchicha L.; Kutsira M.; Kuziyamisa K.; Kuziyamisa M.; Kwangware B.; Lozani P.; Mabuto J.; Mabuto V.; Mabvurwa L.; Machacha R.; Machaya C.; Madembo R.; Madya S.; Madzingira S.; Mafa L.; Mafuta F.; Mafuta J.; Mahara A.; Mahonye S.; Maisva A.; Makara A.; Makover M.; Mambongo E.; Mambure M.; Mandizvidza E.; Mangena G.; Manjengwa E.; Manomano J.; Mapfumo M.; Mapfurire A.; Maphosa L.; Mapundo J.; Mare D.; Marecha F.; Marecha S.; Mashiri C.; Masiya M.; Masuku T.; Masvimbo P.; Matambo S.; Matarise G.; Matinanga L.; Matizanadzo J.; Maunganidze M.; Mawere B.; Mawire C.; Mazvanya Y.; Mbasera M.; Mbono M.; Mhakayakora C.; Mhlanga N.; Mhosva B.; Moyo N.; Moyo O.; Moyo R.; Mpakami C.; Mpedzisi R.; Mpofu E.; Mpofu E.; Mtetwa M.; Muchakachi J.; Mudadada T.; Mudzingwa K.; Mugwira M.; Mukarati T.; Munana A.; Munazo J.; Munyeki O.; Mupfeka P.; Murangandi G.; Muranganwa M.; Murenjekwa J.; Muringo N.; Mushaninga T.; Mutaja F.; Mutanha D.; Mutemeri P.; Mutero B.; Muteya E.; Muvembi S.; Muzenda T.; Mwenjota A.; Ncube S.; Ndabambi T.; Ndava N.; Ndlovu E.; Nene E.; Ngazimbi E.; Ngwalati A.; Nyama T.; Nzembe A.; Pabwaungana E.; Phiri S.; Pukuta R.; Rambanapasi M.; Rera T.; Samanga V.; Shirichena S.; Shoko C.; Shonhe M.; Shuro C.; Sibanda J.; Sibangani E.; Sibangani N.; Sibindi N.; Sitotombe M.; Siwawa P.; Tagwirei M.; Taruvinga P.; Tavagwisa A.; Tete E.; Tete Y.; Thandiwe E.; Tibugari A.; Timothy S.; Tongogara R.; Tshuma L.; Tsikira M.; Tumba C.; Watinaye R.; Zhiradzango E.; Zimunya E.; Zinengwa L.; Ziupfu M.; Ziyambe J.; Church J. A.; Desai A.; Fundira D.; Gough E.; Kambarami R. A.; Matare C. R.; Malaba T. R.; Mupfudze T.; Ngure F.; Smith L. E.; Curtis V.; Dickin K. L.; Habicht J.-P.; Masimirembwa C.; Morgan P.; Pelto G. H.; Sheffner-Rogers C.; Thelingwani R.; Turner P.; Zungu L.; Makadzange T.; Mujuru H. A.; Nyachowe C.; Chakadai R.; Chanyau G.; Makamure M. G.; Chiwariro H.; Mtetwa T.; Chikunya J.; Maguwu L.; Nyadundu S.; Moyo T.; Chayima B.; Mvindi L.; Rwenhamo P.; Muzvarwandoga S.; Chimukangara R.; Njovo H.; Makoni T. Independent and Combined Effects of Improved Water, Sanitation, and Hygiene, and Improved Complementary Feeding, on Child Stunting and Anaemia in Rural Zimbabwe: A Cluster-Randomised Trial. Lancet Global Health 2019, 7 (1), e132–e147. 10.1016/S2214-109X(18)30374-7. 30554749 PMC6293965

[ref21] Luby S. P.; Rahman M.; Arnold B. F.; Unicomb L.; Ashraf S.; Winch P. J.; Stewart C. P.; Begum F.; Hussain F.; Benjamin-Chung J.; Leontsini E.; Naser A. M.; Parvez S. M.; Hubbard A. E.; Lin A.; Nizame F. A.; Jannat K.; Ercumen A.; Ram P. K.; Das K. K.; Abedin J.; Clasen T. F.; Dewey K. G.; Fernald L. C.; Null C.; Ahmed T.; Colford J. M. Jr. Effects of Water Quality, Sanitation, Handwashing, and Nutritional Interventions on Diarrhoea and Child Growth in Rural Bangladesh: A Cluster Randomised Controlled Trial. Lancet Global Health 2018, 6 (3), e302–e315. 10.1016/S2214-109X(17)30490-4. 29396217 PMC5809718

[ref22] Null C.; Stewart C. P.; Pickering A. J.; Dentz H. N.; Arnold B. F.; Arnold C. D.; Benjamin-Chung J.; Clasen T.; Dewey K. G.; Fernald L. C. H.; Hubbard A. E.; Kariger P.; Lin A.; Luby S. P.; Mertens A.; Njenga S. M.; Nyambane G.; Ram P. K.; Colford J. M. Jr. Effects of Water Quality, Sanitation, Handwashing, and Nutritional Interventions on Diarrhoea and Child Growth in Rural Kenya: A Cluster-Randomised Controlled Trial. Lancet Global Health 2018, 6 (3), e316–e329. 10.1016/S2214-109X(18)30005-6. 29396219 PMC5809717

[ref23] Esrey S. A.; Habicht J.-P.; Casella G. The Complementary Effect of Latrines and Increased Water Usage on the Growth of Infants in Rural Lesotho. Am. J. Epidemiol. 1992, 135 (6), 659–666. 10.1093/oxfordjournals.aje.a116345. 1580242

[ref24] Esrey S. A. Water, Waste, and Well-Being: A Multicountry Study. Am. J. Epidemiol. 1996, 143 (6), 608–623. 10.1093/oxfordjournals.aje.a008791. 8610678

[ref25] Pickering A. J.; Davis J. Freshwater Availability and Water Fetching Distance Affect Child Health in Sub-Saharan Africa. Environ. Sci. Technol. 2012, 46 (4), 2391–2397. 10.1021/es203177v. 22242546

[ref26] VanDerslice J.; Briscoe J. Environmental Interventions in Developing Countries: Interactions and Their Implications. Am. J. Epidemiol. 1995, 141 (2), 135–144. 10.1093/oxfordjournals.aje.a117401. 7817969

[ref27] Briscoe J. Intervention Studies And The Definition Of Dominant Transmission Routes. Am. J. Epidemiol. 1984, 120 (3), 449–456. 10.1093/oxfordjournals.aje.a113909. 6475919

[ref28] Eisenberg J. N. S.; Scott J. C.; Porco T. Integrating Disease Control Strategies: Balancing Water Sanitation and Hygiene Interventions to Reduce Diarrheal Disease Burden. Am. J. Public Health 2007, 97 (5), 846–852. 10.2105/AJPH.2006.086207. 17267712 PMC1854876

[ref29] Penakalapati G.; Swarthout J.; Delahoy M. J.; McAliley L.; Wodnik B.; Levy K.; Freeman M. C. Exposure to Animal Feces and Human Health: A Systematic Review and Proposed Research Priorities. Environ. Sci. Technol. 2017, 51 (20), 11537–11552. 10.1021/acs.est.7b02811. 28926696 PMC5647569

[ref30] Pickering A. J.; Null C.; Winch P. J.; Mangwadu G.; Arnold B. F.; Prendergast A. J.; Njenga S. M.; Rahman M.; Ntozini R.; Benjamin-Chung J.; Stewart C. P.; Huda T. M. N.; Moulton L. H.; Colford J. M. J.; Luby S. P.; Humphrey J. H. The WASH Benefits and SHINE Trials: Interpretation of WASH Intervention Effects on Linear Growth and Diarrhoea. Lancet Global Health 2019, 7 (8), e1139–e1146. 10.1016/S2214-109X(19)30268-2. 31303300

[ref31] Prendergast A. J.; Gharpure R.; Mor S.; Viney M.; Dube K.; Lello J.; Berger C.; Siwila J.; Joyeux M.; Hodobo T.; Hurt L.; Brown T.; Hoto P.; Tavengwa N.; Mutasa K.; Craddock S.; Chasekwa B.; Robertson R. C.; Evans C.; Chidhanguro D.; Mutasa B.; Majo F.; Smith L. E.; Hirai M.; Ntozini R.; Humphrey J. H.; Berendes D. Putting the “A” into WaSH: A Call for Integrated Management of Water, Animals, Sanitation, and Hygiene. Lancet Planet. Health 2019, 3 (8), e336–e337. 10.1016/S2542-5196(19)30129-9. 31439312 PMC11287451

[ref32] Ercumen A.; Pickering A. J.; Kwong L. H.; Arnold B. F.; Parvez S. M.; Alam M.; Sen D.; Islam S.; Kullmann C.; Chase C.; Ahmed R.; Unicomb L.; Luby S. P.; Colford J. M. Animal Feces Contribute to Domestic Fecal Contamination: Evidence from *E. coli* Measured in Water, Hands, Food, Flies, and Soil in Bangladesh. Environ. Sci. Technol. 2017, 51 (15), 8725–8734. 10.1021/acs.est.7b01710. 28686435 PMC5541329

[ref33] Boehm A. B.; Wang D.; Ercumen A.; Shea M.; Harris A. R.; Shanks O. C.; Kelty C.; Ahmed A.; Mahmud Z. H.; Arnold B. F.; Chase C.; Kullmann C.; Colford J. M.; Luby S. P.; Pickering A. J. Occurrence of Host-Associated Fecal Markers on Child Hands, Household Soil, and Drinking Water in Rural Bangladeshi Households. Environ. Sci. Technol. Lett. 2016, 3 (11), 393–398. 10.1021/acs.estlett.6b00382. 32607385 PMC7326215

[ref34] Harris A. R.; Pickering A. J.; Harris M.; Doza S.; Islam M. S.; Unicomb L.; Luby S.; Davis J.; Boehm A. B. Ruminants Contribute Fecal Contamination to the Urban Household Environment in Dhaka, Bangladesh. Environ. Sci. Technol. 2016, 50 (9), 4642–4649. 10.1021/acs.est.5b06282. 27045990

[ref35] Fuhrmeister E. R.; Ercumen A.; Pickering A. J.; Jeanis K. M.; Ahmed M.; Brown S.; Arnold B. F.; Hubbard A. E.; Alam M.; Sen D.; Islam S.; Kabir M. H.; Kwong L. H.; Islam M.; Unicomb L.; Rahman M.; Boehm A. B.; Luby S. P.; Colford J. M.; Nelson K. L. Predictors of Enteric Pathogens in the Domestic Environment from Human and Animal Sources in Rural Bangladesh. Environ. Sci. Technol. 2019, 53 (17), 10023–10033. 10.1021/acs.est.8b07192. 31356066 PMC6727619

[ref36] Odagiri M.; Schriewer A.; Daniels M. E.; Wuertz S.; Smith W. A.; Clasen T.; Schmidt W.-P.; Jin Y.; Torondel B.; Misra P. R.; Panigrahi P.; Jenkins M. W. Human Fecal and Pathogen Exposure Pathways in Rural Indian Villages and the Effect of Increased Latrine Coverage. Water Res. 2016, 100, 232–244. 10.1016/j.watres.2016.05.015. 27192358 PMC4907306

[ref37] Hamzah L.; Boehm A. B.; Davis J.; Pickering A. J.; Wolfe M.; Mureithi M.; Harris A. Ruminant Fecal Contamination of Drinking Water Introduced Post-Collection in Rural Kenyan Households. Int. J. Environ. Res. Public Health 2020, 17 (2), 608 10.3390/ijerph17020608. 31963600 PMC7027003

[ref38] Headey D.; Hirvonen K. Is Exposure to Poultry Harmful to Child Nutrition? An Observational Analysis for Rural Ethiopia. PLoS One 2016, 11 (8), e0160590 10.1371/journal.pone.0160590. 27529178 PMC4986937

[ref39] Rawlins R.; Pimkina S.; Barrett C. B.; Pedersen S.; Wydick B. Got Milk? The Impact of Heifer International’s Livestock Donation Programs in Rwanda on Nutritional Outcomes. Food Policy 2014, 44, 202–213. 10.1016/j.foodpol.2013.12.003.

[ref40] Mosites E. M.; Rabinowitz P. M.; Thumbi S. M.; Montgomery J. M.; Palmer G. H.; May S.; Rowhani-Rahbar A.; Neuhouser M. L.; Walson J. L. The Relationship between Livestock Ownership and Child Stunting in Three Countries in Eastern Africa Using National Survey Data. PLoS One 2015, 10 (9), e0136686 10.1371/journal.pone.0136686. 26361393 PMC4567267

[ref41] Hossain M. B. MSc.; Khan J. R. MSc. Association between Household Livestock Ownership and Childhood Stunting in Bangladesh – A Spatial Analysis. J. Trop. Pediatr. 2020, 66 (3), 248–256. 10.1093/tropej/fmz061. 32452522

[ref42] Bardosh K. L.; Hussein J. W.; Sadik E. A.; Hassen J. Y.; Ketema M.; Ibrahim A. M.; McKune S. L.; Havelaar A. H. Chicken Eggs, Childhood Stunting and Environmental Hygiene: An Ethnographic Study from the Campylobacter Genomics and Environmental Enteric Dysfunction (CAGED) Project in Ethiopia. One Health Outlook 2020, 2 (1), 5 10.1186/s42522-020-00012-9. 33829128 PMC7993501

[ref43] Zambrano L. D.; Levy K.; Menezes N. P.; Freeman M. C. Human Diarrhea Infections Associated with Domestic Animal Husbandry: A Systematic Review and Meta-Analysis. Trans. R. Soc. Trop. Med. Hyg. 2014, 108 (6), 313–325. 10.1093/trstmh/tru056. 24812065 PMC4023907

[ref44] George C. M.; Oldja L.; Biswas S. K.; Perin J.; Lee G. O.; Ahmed S.; Haque R.; Sack R. B.; Parvin T.; Azmi I. J.; Bhuyian S. I.; Talukder K. A.; Faruque A. G. Fecal Markers of Environmental Enteropathy Are Associated with Animal Exposure and Caregiver Hygiene in Bangladesh. Am. J. Trop. Med. Hyg. 2015, 93 (2), 269–275. 10.4269/ajtmh.14-0694. 26055734 PMC4530746

[ref45] Arnold B. F.; Null C.; Luby S. P.; Unicomb L.; Stewart C. P.; Dewey K. G.; Ahmed T.; Ashraf S.; Christensen G.; Clasen T.; Dentz H. N.; Fernald L. C. H.; Haque R.; Hubbard A. E.; Kariger P.; Leontsini E.; Lin A.; Njenga S. M.; Pickering A. J.; Ram P. K.; Tofail F.; Winch P. J.; Colford J. M. Cluster-Randomised Controlled Trials of Individual and Combined Water, Sanitation, Hygiene and Nutritional Interventions in Rural Bangladesh and Kenya: The WASH Benefits Study Design and Rationale. BMJ Open 2013, 3 (8), e003476 10.1136/bmjopen-2013-003476. PMC3758977 23996605

[ref46] Pickering A. J.; Njenga S. M.; Steinbaum L.; Swarthout J.; Lin A.; Arnold B. F.; Stewart C. P.; Dentz H. N.; Mureithi M.; Chieng B.; Wolfe M.; Mahoney R.; Kihara J.; Byrd K.; Rao G.; Meerkerk T.; Cheruiyot P.; Papaiakovou M.; Pilotte N.; Williams S. A.; Colford J. M. Jr.; Null C. Effects of Single and Integrated Water, Sanitation, Handwashing, and Nutrition Interventions on Child Soil-Transmitted Helminth and Giardia Infections: A Cluster-Randomized Controlled Trial in Rural Kenya. PLoS Med. 2019, 16 (6), e1002841 10.1371/journal.pmed.1002841. 31242190 PMC6594579

[ref47] Lin A.; Ali S.; Arnold B. F.; Rahman M. Z.; Alauddin M.; Grembi J.; Mertens A. N.; Famida S. L.; Akther S.; Hossen M. S.; Mutsuddi P.; Shoab A. K.; Hussain Z.; Rahman M.; Unicomb L.; Ashraf S.; Naser A. M.; Parvez S. M.; Ercumen A.; Benjamin-Chung J.; Haque R.; Ahmed T.; Hossain M. I.; Choudhury N.; Jannat K.; Alauddin S. T.; Minchala S. G.; Cekovic R.; Hubbard A. E.; Stewart C. P.; Dewey K. G.; Colford J. M. Jr.; Luby S. P. Effects of Water, Sanitation, Handwashing, and Nutritional Interventions on Environmental Enteric Dysfunction in Young Children: A Cluster-Randomized, Controlled Trial in Rural Bangladesh. Clin. Infect. Dis. 2019, 70 (5), 738–747. 10.1093/cid/ciz291. 30963177

[ref48] Stewart C. P.; Kariger P.; Fernald L.; Pickering A. J.; Arnold C. D.; Arnold B. F.; Hubbard A. E.; Dentz H. N.; Lin A.; Meerkerk T. J.; Milner E.; Swarthout J.; Colford J. M. Jr; Null C. Effects of Water Quality, Sanitation, Handwashing, and Nutritional Interventions on Child Development in Rural Kenya (WASH Benefits Kenya): A Cluster-Randomised Controlled Trial. Lancet Child Adolesc. Health 2018, 2 (4), 269–280. 10.1016/S2352-4642(18)30025-7. 29616236 PMC5859215

[ref49] Kwong L. H.; Ercumen A.; Pickering A. J.; Unicomb L.; Davis J.; Luby S. P. Hand- and Object-Mouthing of Rural Bangladeshi Children 3–18 Months Old. Int. J. Environ. Res. Public Health 2016, 13 (6), 563 10.3390/ijerph13060563. 27271651 PMC4924020

[ref50] Vujcic J.; Ram P. K.; Hussain F.; Unicomb L.; Gope P. S.; Abedin J.; Mahmud Z. H.; Islam M. S.; Luby S. P. Toys and Toilets: Cross-Sectional Study Using Children’s Toys to Evaluate Environmental Faecal Contamination in Rural Bangladeshi Households with Different Sanitation Facilities and Practices. Trop. Med. Int. Health 2014, 19 (5), 528–536. 10.1111/tmi.12292. 24645919

[ref51] Cogill B. Anthropometric Indicators Measurement Guide; Food and Nutrition Technical Assistance Project, 2001.

[ref52] de Onis M.; Garza C.; Victora C. G.; Onyango A. W.; Frongillo E. A.; Martines J. The Who Multicentre Growth Reference Study: Planning, Study Design, and Methodology. Food Nutr. Bull. 2004, 25 (1_suppl_1), S15–S26. 10.1177/15648265040251S104. 15069916

[ref53] The WHO Child Growth Standards, 2022. https://www.who.int/tools/child-growth-standards/standards. accessed May 27, 2022–05–27.

[ref54] Yelland L. N.; Salter A. B.; Ryan P. Performance of the Modified Poisson Regression Approach for Estimating Relative Risks from Clustered Prospective Data. Am. J. Epidemiol. 2011, 174 (8), 984–992. 10.1093/aje/kwr183. 21841157

[ref55] Zou G. A Modified Poisson Regression Approach to Prospective Studies with Binary Data. Am. J. Epidemiol. 2004, 159 (7), 702–706. 10.1093/aje/kwh090. 15033648

[ref56] Balzer L. B.; van der Laan M. J.; Petersen M. L. Adaptive Pre-Specification in Randomized Trials With and Without Pair-Matching. Stat. Med. 2016, 35 (25), 4528–4545. 10.1002/sim.7023. 27436797 PMC5084457

[ref57] Montealegre M. C.; Roy S.; Boni F.; Hossain M. I.; Navab-Daneshmand T.; Caduff L.; Faruque A. S. G.; Islam M. A.; Julian T. R. Risk Factors for Detection, Survival, and Growth of Antibiotic-Resistant and Pathogenic *Escherichia Coli* in Household Soils in Rural Bangladesh. Appl. Environ. Microbiol. 2018, 84 (24), e01978-18 10.1128/AEM.01978-18. 30315075 PMC6275341

[ref58] Jahnke H. E. Livestock Production Systems and Livestock Development in Tropical Africa; Kieler Wissenschaftsverlag Vauk: Kiel, Germany, 1982.

[ref59] Njuki J.; Poole E. J.; Johnson J.; Baltenweck I.; Pali P. N.; Lokman Z.; Mburu S. Gender, Livestock and Livelihood Indicators. 2011.

[ref60] van der Laan M. J.; Polley E. C.; Hubbard A. E. Super Learner. Stat. Appl. Genet. Mol. Biol. 2007, 6, 1–6. 10.2202/1544-6115.1309. 17910531

[ref61] Enger K. S.; Nelson K. L.; Rose J. B.; Eisenberg J. N. S. The Joint Effects of Efficacy and Compliance: A Study of Household Water Treatment Effectiveness against Childhood Diarrhea. Water Res. 2013, 47 (3), 1181–1190. 10.1016/j.watres.2012.11.034. 23290123

[ref62] Brown J.; Clasen T. High Adherence Is Necessary to Realize Health Gains from Water Quality Interventions. PLoS One 2012, 7 (5), e36735 10.1371/journal.pone.0036735. 22586491 PMC3346738

[ref63] Daniels M. E.; Smith W. A.; Schmidt W.-P.; Clasen T.; Jenkins M. W. Modeling Cryptosporidium and Giardia in Ground and Surface Water Sources in Rural India: Associations with Latrines, Livestock, Damaged Wells, and Rainfall Patterns. Environ. Sci. Technol. 2016, 50 (14), 7498–7507. 10.1021/acs.est.5b05797. 27310009 PMC5058636

[ref64] Kotloff K. L.; Nasrin D.; Blackwelder W. C.; Wu Y.; Farag T.; Panchalingham S.; Sow S. O.; Sur D.; Zaidi A. K. M.; Faruque A. S. G.; Saha D.; Alonso P. L.; Tamboura B.; Sanogo D.; Onwuchekwa U.; Manna B.; Ramamurthy T.; Kanungo S.; Ahmed S.; Qureshi S.; Quadri F.; Hossain A.; Das S. K.; Antonio M.; Hossain M. J.; Mandomando I.; Acácio S.; Biswas K.; Tennant S. M.; Verweij J. J.; Sommerfelt H.; Nataro J. P.; Robins-Browne R. M.; Levine M. M. The Incidence, Aetiology, and Adverse Clinical Consequences of Less Severe Diarrhoeal Episodes among Infants and Children Residing in Low-Income and Middle-Income Countries: A 12-Month Case-Control Study as a Follow-on to the Global Enteric Multicenter Study (GEMS). Lancet Global Health 2019, 7 (5), e568–e584. 10.1016/S2214-109X(19)30076-2. 31000128 PMC6484777

[ref65] Wolf J.; Hubbard S.; Brauer M.; Ambelu A.; Arnold B. F.; Bain R.; Bauza V.; Brown J.; Caruso B. A.; Clasen T.; Colford J. M.; Freeman M. C.; Gordon B.; Johnston R. B.; Mertens A.; Prüss-Ustün A.; Ross I.; Stanaway J.; Zhao J. T.; Cumming O.; Boisson S. Effectiveness of Interventions to Improve Drinking Water, Sanitation, and Handwashing with Soap on Risk of Diarrhoeal Disease in Children in Low-Income and Middle-Income Settings: A Systematic Review and Meta-Analysis. Lancet 2022, 400 (10345), 48–59. 10.1016/S0140-6736(22)00937-0. 35780792 PMC9251635

[ref66] Jang J.; Hur H.-G.; Sadowsky M.; Byappanahalli M.; Yan T.; Ishii S. Environmental *Escherichia Coli*: Ecology and Public Health Implications—a Review. J. Appl. Microbiol. 2017, 123 (3), 570–581. 10.1111/jam.13468. 28383815

[ref67] Ercumen A.; Mertens A.; Arnold B. F.; Benjamin-Chung J.; Hubbard A. E.; Ahmed M. A.; Kabir M. H.; Khalil M. M. R.; Kumar A.; Rahman M. S.; Parvez S. M.; Unicomb L.; Rahman M.; Ram P. K.; Clasen T.; Luby S. P.; Colford J. M. Effects of Single and Combined Water, Sanitation and Handwashing Interventions on Fecal Contamination in the Domestic Environment: A Cluster-Randomized Controlled Trial in Rural Bangladesh. Environ. Sci. Technol. 2018, 52 (21), 12078–12088. 10.1021/acs.est.8b05153. 30256102 PMC6222549

[ref68] Förster M.; Sievert K.; Messler S.; Klimpel S.; Pfeffer K. Comprehensive Study on the Occurrence and Distribution of Pathogenic Microorganisms Carried by Synanthropic Flies Caught at Different Rural Locations in Germany. J. Med. Entomol. 2009, 46 (5), 1164–1166. 10.1603/033.046.0526. 19769050

[ref69] Szalanski A. L.; Owens C. B.; McKay T.; Steelman C. D. Detection of Campylobacter and *Escherichia Coli* O157:H7 from Filth Flies by Polymerase Chain Reaction. Med. Vet. Entomol. 2004, 18 (3), 241–246. 10.1111/j.0269-283X.2004.00502.x. 15347391

[ref70] Chavasse D. C.; Shier R. P.; Murphy O. A.; Huttly S. R.; Cousens S. N.; Akhtar T. Impact of Fly Control on Childhood Diarrhoea in Pakistan: Community-Randomised Trial. Lancet 1999, 353 (9146), 22–25. 10.1016/S0140-6736(98)03366-2. 10023946

[ref71] Doza S.; Rahman M. J.; Islam M. A.; Kwong L. H.; Unicomb L.; Ercumen A.; Pickering A. J.; Parvez S. M.; Naser A. M.; Ashraf S.; Das K. K.; Luby S. P. Prevalence and Association of *Escherichia Coli* and Diarrheagenic *Escherichia Coli* in Stored Foods for Young Children and Flies Caught in the Same Households in Rural Bangladesh. Am. J. Trop. Med. Hyg. 2018, 98 (4), 1031–1038. 10.4269/ajtmh.17-0408. 29436348 PMC5928814

[ref72] Pickering A. J.; Djebbari H.; Lopez C.; Coulibaly M.; Alzua M. L. Effect of a Community-Led Sanitation Intervention on Child Diarrhoea and Child Growth in Rural Mali: A Cluster-Randomised Controlled Trial. Lancet Global Health 2015, 3 (11), e701–e711. 10.1016/S2214-109X(15)00144-8. 26475017

[ref73] Emerson P. M.; Lindsay S. W.; Alexander N.; Bah M.; Dibba S. M.; Faal H. B.; Lowe K. O.; McAdam K. P. W. J.; Ratcliffe A. A.; Walraven G. E. L.; Bailey R. L. Role of Flies and Provision of Latrines in Trachoma Control: Cluster-Randomised Controlled Trial. Lancet 2004, 363 (9415), 1093–1098. 10.1016/S0140-6736(04)15891-1. 15064026

[ref74] Nazni W. A.; Luke H.; Rozita W. M. W.; Abdullah A. G.; Sa’diyah I.; Azahari A. H.; Zamree I.; Tan S. B.; Lee H. L.; Sofian-Azirun M. Determination of the Flight Range and Dispersal of the House Fly, Musca Domestica (L.) Using Mark Release Recapture Technique. Trop. Biomed. 2005, 22 (1), 53–61. 16880754

[ref75] Enahoro D.; Lannerstad M.; Pfeifer C.; Dominguez-Salas P. Contributions of Livestock-Derived Foods to Nutrient Supply under Changing Demand in Low- and Middle-Income Countries. Global Food Secur. 2018, 19, 1–10. 10.1016/j.gfs.2018.08.002.

[ref76] Dupuis S.; Hennink M.; Wendt A. S.; Waid J. L.; Kalam M. A.; Gabrysch S.; Sinharoy S. S. Women’s Empowerment through Homestead Food Production in Rural Bangladesh. BMC Public Health 2022, 22 (1), 134 10.1186/s12889-022-12524-2. 35045859 PMC8772198

[ref77] Randremanana R. V.; Razafindratsimandresy R.; Andriatahina T.; Randriamanantena A.; Ravelomanana L.; Randrianirina F.; Richard V. Etiologies, Risk Factors and Impact of Severe Diarrhea in the Under-Fives in Moramanga and Antananarivo, Madagascar. PLoS One 2016, 11 (7), e0158862 10.1371/journal.pone.0158862. 27411101 PMC4943590

[ref78] Kaur M.; Graham J. P.; Eisenberg J. N. S. Livestock Ownership Among Rural Households and Child Morbidity and Mortality: An Analysis of Demographic Health Survey Data from 30 Sub-Saharan African Countries (2005–2015). Am. J. Trop. Med. Hyg. 2017, 96 (3), 741–748. 10.4269/ajtmh.16-0664. 28044044 PMC5361555

[ref79] Chen D.; Mechlowitz K.; Li X.; Schaefer N.; Havelaar A. H.; McKune S. L. Benefits and Risks of Smallholder Livestock Production on Child Nutrition in Low- and Middle-Income Countries. Front. Nutr. 2021, 8, 751686 10.3389/fnut.2021.751686. 34778344 PMC8579112

[ref80] Goddard F. G. B.; Pickering A. J.; Ercumen A.; Brown J.; Chang H. H.; Clasen T. Faecal Contamination of the Environment and Child Health: A Systematic Review and Individual Participant Data Meta-Analysis. Lancet Planet. Health 2020, 4 (9), e405–e415. 10.1016/S2542-5196(20)30195-9. 32918886 PMC7653404

[ref81] Luby S. P.; Mendoza C.; Keswick B. H.; Chiller T. M.; Hoekstra R. M. Difficulties in Bringing Point-of-Use Water Treatment to Scale in Rural Guatemala. Am. J. Trop. Med. Hyg. 2008, 78 (3), 382–387. 10.4269/ajtmh.2008.78.382. 18337330

[ref82] Arnold B.; Arana B.; Mäusezahl D.; Hubbard A.; Colford J. M. Evaluation of a Pre-Existing, 3-Year Household Water Treatment and Handwashing Intervention in Rural Guatemala. Int. J. Epidemiol. 2009, 38 (6), 1651–1661. 10.1093/ije/dyp241. 19574492 PMC2786251

[ref83] Fewtrell L.; Kaufmann R. B.; Kay D.; Enanoria W.; Haller L.; Colford J. M. Water, Sanitation, and Hygiene Interventions to Reduce Diarrhoea in Less Developed Countries: A Systematic Review and Meta-Analysis. Lancet Infect. Dis. 2005, 5 (1), 42–52. 10.1016/S1473-3099(04)01253-8. 15620560

[ref84] Lin A.; Ercumen A.; Benjamin-Chung J.; Arnold B. F.; Das S.; Haque R.; Ashraf S.; Parvez S. M.; Unicomb L.; Rahman M.; Hubbard A. E.; Stewart C. P.; Colford J. M.; Luby S. P. Effects of Water, Sanitation, Handwashing, and Nutritional Interventions on Child Enteric Protozoan Infections in Rural Bangladesh: A Cluster-Randomized Controlled Trial. Clin. Infect. Dis. 2018, 67 (10), 1515–1522. 10.1093/cid/ciy320. 29669039 PMC6206106

[ref85] Luby S. P.; Agboatwalla M.; Painter J.; Altaf A.; Billhimer W.; Keswick B.; Hoekstra R. M. Combining Drinking Water Treatment and Hand Washing for Diarrhoea Prevention, a Cluster Randomised Controlled Trial. Trop. Med. Int. Health 2006, 11 (4), 479–489. 10.1111/j.1365-3156.2006.01592.x. 16553931

[ref86] Briceño B.; Coville A.; Gertler P.; Martinez S. Are There Synergies from Combining Hygiene and Sanitation Promotion Campaigns: Evidence from a Large-Scale Cluster-Randomized Trial in Rural Tanzania. PLoS One 2017, 12 (11), e0186228 10.1371/journal.pone.0186228. 29091726 PMC5665426

