# Supplementary material for: Addressing Fecal Contamination in Rural Kenyan Households: The Roles of Environmental Interventions and Animal Ownership
Source: Environ Sci Technol. 2024 May 17;58(22):9500–14. doi: 10.1021/acs.est.3c09419 (PMC11155254; doi:10.1021/acs.est.3c09419)
Supplement: Supplementary file 1 — es3c09419_si_001.pdf [file es3c09419_si_001.pdf]

Supporting Information

Addressing fecal contamination in rural Kenyan households: the roles of environmental interventions and animal ownership

Jenna M. Swarthout, Maryanne Mureithi, John Mboya, Benjamin F. Arnold, Marlene Wolfe, Holly N. Dentz, Audrie Lin, Charles D. Arnold, Gouthami Rao, Christine P. Stewart, Thomas Clasen, John M. Colford, Jr., Clair Null, Amy J. Pickering

Table of Contents

Supporting Information S1: Additional methods details S3

Supporting Information S2: Additional results S10

Figure S1: WASH Benefits Kenya interventions. S11

Figure S2: Directed acyclic graph for E. coli contamination in stored water S12

Figure S3: Directed acyclic graph for E. coli contamination on child hands S12

Figure S4: Directed acyclic graph for E. coli contamination on sentinel toys S13

Figure S5: Directed acyclic graph for fly counts S13

Figure S6: Directed acyclic graph for diarrhea S14

Figure S7: Directed acyclic graph for length-for-age Z-scores S14

Figure S8: Associations between $\log_{10}$ number of animals owned by households and stored water contamination among households with and without detectable free chlorine. S15

Table S1: E. coli measured at one- and two-year assessments, combined and separately, interventions vs. control. S16

Table S2: Total coliforms (water and hand samples) or fecal coliforms (toy samples) measured at one- and two-year assessments, combined and separately, interventions vs. control. S17

Table S3: Fly counts and dirt on hands/fingernails measured at one- and two-year assessments, combined and separately, interventions vs. control. S18

Table S4: Fly counts and dirt on hands/fingernails measured at one- and two-year assessments, combined and separately, interventions vs. control. S20

Table S5: E. coli and fly prevalence and concentration measured at one- and two-year assessments, combined and separately, interventions vs. control, subgroup analysis by season. S21

Figure S9: Associations between household TLU scores and environmental contamination. S24

Figure S10: Associations between binary household animal ownership and environmental contamination. S25

Figure S11: Associations between binary household animal ownership (households with extreme predicted probabilities excluded) and environmental contamination. S26

Figure S12: Associations between $\log_{10}$ median number of animals owned by households in the same village and environmental contamination. S27

Figure S13: Associations between household TLU scores and child health. S28

Figure S14: Associations between binary household animal ownership and child health. S29

Figure S15: Associations between binary household animal ownership (households with extreme predicted probabilities excluded) and child health. S30

Figure S16: Associations between binary household animal ownership and stunting outcomes, accounting for households that changed animal ownership status between enrollment and year two. S31

Figure S17: Associations between binary household animal ownership and stunting outcomes, accounting for subgroups of households that changed animal ownership status between enrollment and year two. S32

Figure S18: Associations between $\log_{10}$ median number of animals owned by households in the same village and child health.. .... S33

Supporting Information S1: Additional methods details

Study Design

The intervention arms included water treatment (W), improved sanitation (S), handwashing with soap (H), combined WSH, nutritional interventions, and combined WSH and nutritional interventions (WSHN). The WSH interventions were designed to improve the environmental conditions of the children in the first two years of life to reduce early-life exposure to fecal pathogens. The water treatment intervention included installation of chlorine dispensers at community water locations plus provision of bottled chlorine to enrolled households. The sanitation intervention included pit latrine upgrades with a reinforced slab and drop hole cover, as well as child potties and scoops to remove human or animal feces from households and compounds. The handwashing intervention included dual dispenser tippy-tap devices operated with independent pedals, each providing one container for soapy water (soap delivered by community promoters) and one container for rinse water, installed near the latrine and kitchen area.[1] The nutrition intervention included lipid-based nutrient supplements (LNS) and age-appropriate recommendations on maternal nutrition and infant feeding practices. Local promoters visited study compounds at least every other month to deliver relevant behavior change messages for the treatment group assigned.

Clusters were defined as one or two, and in rare cases three, adjoining villages with at least six eligible pregnant women. Nine clusters were used to form a geographic block; clusters were randomized into the eight study arms (six intervention arms, active

[1] Amin, N., et al. 2014. Microbiological evaluation of the efficacy of soapy water to clean hands: A randomized, non-inferiority field trial. *Am J Trop Med Hyg*, 91(2): 415-423.

control, passive control) within each block (geographically pair-matched randomization increases the efficiency of effect estimates as study outcomes are expected to be correlated with geographical characteristics). The EED subset of the trial for which the one- and two-year environmental assessments were conducted consisted of 54 clusters per arm in four study arms (control, nutrition, WSH, WSHN), with seven households per cluster. This resulted in a total sample size of 1,500 households for the environmental assessment (375 households per arm), where 750 households (WSH and WSHN arms) received the combined WSH intervention, and 750 households (control and nutrition arms) served as comparisons. We assumed an intra-class correlation coefficient of 0.2 based on a previous large-scale evaluation in rural Bangladesh and a design effect of 2.2.[2] With this sample size of 750 households per comparison group, we expected the following minimum detectable effect sizes in intervention (WSH and WSHN pooled) vs. comparison (control and nutrition pooled) groups: (1) 0.2 $\log_{10}$ reduction in *E. coli* concentration in stored water, child hand rinse, and toy rinse samples and (2) 7.6 flies difference from 11.5 flies in control in the latrine and food preparation areas.

Sample and Data Collection and Processing

We collected samples of stored drinking water, child hand rinses, and sentinel toy object rinses, as well as enumerated and speciated flies caught in the food preparation and latrine areas of the compound. We also recorded the presence of visible dirt on children's and caregivers' hands, including fingernails, fingerpads, and palms. We

[2] Huda, T. M. N., et al. 2012. Interim evaluation of a large scale sanitation, hygiene and water improvement programme on childhood diarrhea and respiratory disease in rural Bangladesh. *Soc Sci Med*, 75(4): 604-611.

collected stored drinking water samples from a subset of households in the water treatment single arm (those geographically matched with EED enrolled clusters) and from a subset of households in the handwashing single arm (those geographically matched with EED enrolled clusters). These subsets were selected based on household enrollment in the same randomization block as the EED cohort. We calculated fly densities at latrines and food preparation areas in a subset of households in the sanitation single arm enrolled into the same blocks as the EED cohort.

To sample drinking water, field staff asked the respondent to provide a glass of water as if giving it to the respondent's under-five children and recorded whether the water had been obtained directly from the source or from a storage container. Field staff asked the respondent to pour the water from the glass into a sterile Whirlpak bag; if the respondent reported treating the water with chlorine, a Whirlpak bag with sodium thiosulfate tablets was used to neutralize the residual chlorine. Stored water field blanks were collected by pouring a bottle of distilled water (prepared in the lab and carried to the field in a cooler) into a Whirlpak collection bag in the field. To collect child hand rinse samples, field staff asked the respondent to place the child's left hand into a sterile Whirlpak bag pre-filled with 250 mL of distilled water. The hand was massaged from the outside of the bag for 15 seconds, followed by 15 seconds of shaking. The procedure was repeated with the right hand in the same bag, and the rinse water was preserved in the Whirlpak bag. Hand rinse field blanks were collected by opening and closing an unused hand rinse Whirlpak bag in the field. Field staff provided a previously sterilized plastic toy ball to the under-five child and encouraged the respondent to prompt the child to play with the ball. Field staff returned to the households 24 hours later to collect

a rinse sample from the ball by placing the ball into a sterile Whirlpak bag pre-filled with 300 mL of distilled water and massaging it from the outside of the bag for 15 seconds, followed by 15 seconds of shaking. Toy rinse field blanks were collected by opening and closing an unused toy rinse Whirlpak bag in the field. Field staff identified a suitable location in the food preparation area (away from smoke) and latrine area (under a roof or protected from rain). At each location, they placed one scudder fly grill on the ground and took two 90 second measurements within 30 seconds of each other to record the number of flies landing on the grill and categorize the captured flies as house flies (*Musca domestica*, *Fannia canicularis*, *stomoxys*), bottle flies (*Lucilia*, *chrysomya*, *calliphora*), flesh flies (*Sarcophaga*), or other flies using a visual identification chart.

All water, hand rinse, and toy rinse samples were preserved on ice in coolers and transported to the field laboratory to be processed on the same day, typically within eight hours of collection. 100 mL was filtered per stored water sample and stored water field blank sample, 50 mL filtered per hand rinse sample and hand rinse field blank sample, and 100 mL filtered per toy rinse sample and toy rinse field blank sample. Lab technicians targeted processing 5% duplicates for each sample type by filtering the same volume from the same collection bag. Lab blanks were also processed daily by each lab technician by filtering 50 mL of distilled water. Stored water and hand rinse samples were incubated at 35 °C, while toy samples were incubated at 44.5 °C. Previous work demonstrated fecal coliforms incubated at 44.5°C on toy balls to be an indicator of environmental contamination associated with sanitation conditions.[3] *E. coli*

[3] Vujcic, J., et al. 2014. Toys and toilets: cross-sectional study using children's toys to evaluate environmental faecal contamination in rural Bangladeshi households with different sanitation facilities and practices. *Trop Med Int Health*, 19(5): 528-536.

was the primary indicator of microbial contamination (reported in main text); total and fecal coliform counts were secondary indicators (reported in SI only).

Statistical Analyses

Post-incubation on MI media, stored water, hand rinse, and toy rinse samples classified as “too numerous to count” were assigned a value of 500 colonies per plate. Colony forming units (CFU) of *E. coli* were $\log_{10}$-transformed for analysis of discrete variables. Samples below the detection limit were substituted with half the lower detection limit (0.5 colonies per plate) prior to log transformation.

Our primary environmental outcomes for all analyses included *E. coli* prevalence and concentration in water, hand rinse, and toy rinse samples, and the presence and number of flies at food preparation areas and latrines (intervention impact analyses only). Secondary outcomes for the intervention impact analyses included fecal coliform prevalence and concentration in water, hand rinse, and toy rinse samples and the percentage of children and caregivers with visible dirt on hands. We report subgroup analyses by year of data collection (year one versus year two) in the main text and by season (wet versus dry season) in the SI only. Wet season included the months of April, May, August, September, October, and November; dry season included January, February, March, June, July, and December.

We pre-screened covariates to assess whether they were associated with each outcome prior to including them in adjusted statistical models.[4] We used the likelihood

[4] Pocock, S. J., et al. 2002. Subgroup analysis, covariate adjustment and baseline comparisons in clinical trial reporting: current practice and problems. *Stat Med*, 21(19): 2917-2930.

ratio test to assess the association between each outcome and each covariate and included covariates with a *p*-value < 0.2 in the adjusted analysis. We also excluded covariates that had little variation in the study population (prevalence < 5%).

The following baseline covariates were tested for inclusion in the adjusted models:

- Month of measurement, to account for seasonal variation
- Child age (days)
- Child sex
- Maternal age (years)
- Maternal education level (no education/incomplete primary, complete primary, incomplete secondary, complete secondary)
- Number of children <18 years in the household
- Number of individuals living in the compound
- Distance (in minutes) to the household's primary drinking water source
- Housing materials (floor, walls, roof)
- Household assets assessed individually
    - Electricity, radio, television, mobile phone, clock, bicycle, motorcycle, stove, gas cooker, car
    - Number of cows, goats, dogs, and chickens (intervention impacts analyses only)
- Intervention arm (animal ownership analyses only)

Additionally, we considered the following covariates for all environmental contamination outcomes (*E. coli* in stored water, child hand rinses, sentinel toy rinses and fly counts):

- Staff member ID of staff member who collected measurement

- Most recent time it rained (now, today, yesterday, day before yesterday, last week, or before last week, as reported by respondent)

We also tested the following covariates that would be expected to affect fecal indicator bacteria levels for these specific types of samples. While these covariates were measured at the time of the environmental assessments (rather than at baseline), we did not expect them to be impacted by the study interventions.

Sentinel Toys

- Location of toy ball at time of sample collection (indoors, outdoors)
- How much children played with ball (several times (4+), few times (2-3), once, never)
- Whether children from other compounds played with ball
- Where children played with ball (indoors, outdoors)
- Whether respondent cleaned ball prior to sample collection
- Condition of ball at time of sample collection (intact/bulging, deflated/burst/torn)

Fly Counts

- Location of food preparation area (indoors vs. outdoors)

The following covariates were also considered for child health outcomes (diarrhea, stunting, length-for-age *Z*-scores):

- Maternal height (cm)

- Food insecurity of household measured using the Household Hunger Scale (HHS)[5] (little-to-no hunger, moderate-to-severe hunger)

Supporting Information S2: Additional results

Blanks and Duplicates

E. coli was detected in 0.1% (1/832) of lab blanks, and the log-transformed *E. coli* concentration in the positive blank was 3.0 CFU/100 mL. Across sample types and sampling rounds, *E. coli* was detected in 2.1% (22/1035) of field blanks, and the log-transformed geometric mean *E. coli* concentration among positive blanks was 1.0 CFU/100 mL. Agreement between *E. coli* presence in duplicates was 91% (kappa statistic = 0.71). The Pearson correlation coefficient between *E. coli* concentrations in duplicates was 0.97.

[5] FANTA. 2011. Household hunger scale (HHS): Indicator definition and measurement guide. https://www.fantaproject.org/monitoring-and-evaluation/household-hunger-scale-hhs

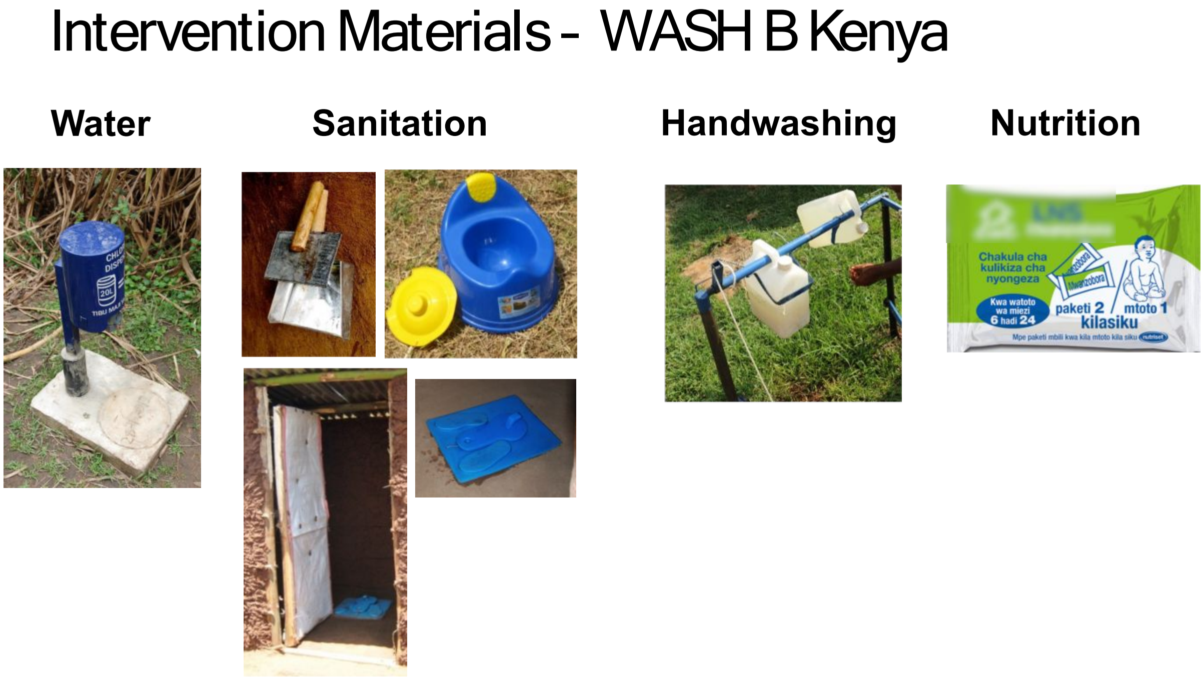

Figure S1: WASH Benefits Kenya interventions. From left to right, the photos represent the (1) water (chlorine dispensers placed at community water sources), (2) sanitation (sani-scoopers, child potties, and pit latrines with plastic slabs and drop hole covers), (3) handwashing (handwashing stations with water and soapy water in 5-liter jerry cans), and (4) nutrition (infant and young child feeding counseling and lipid-based nutrient supplements) interventions.

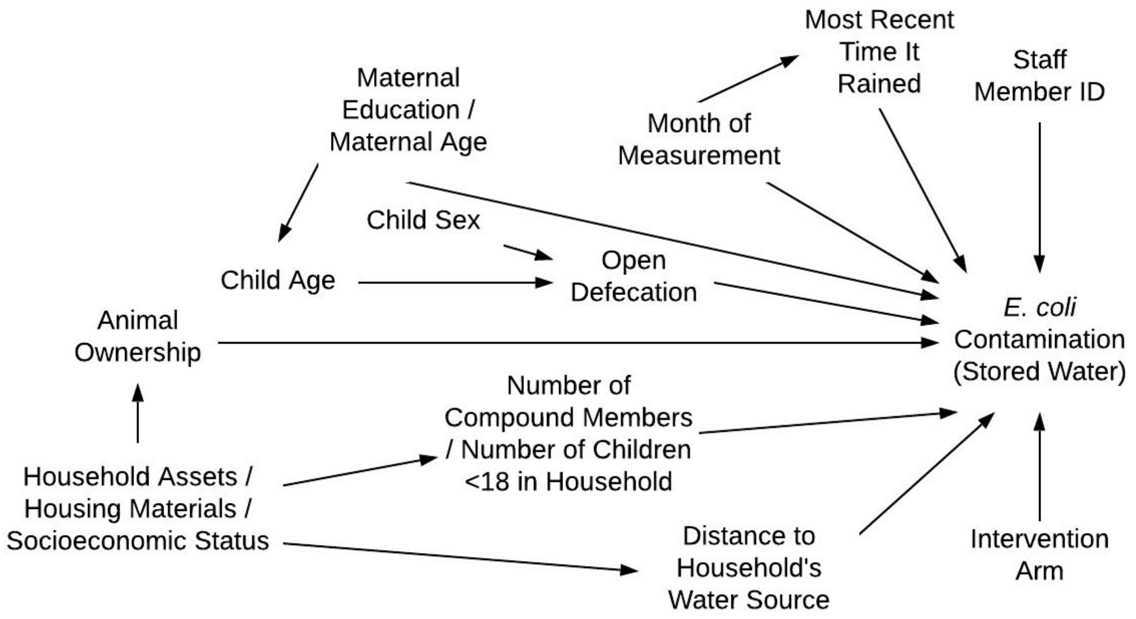

Figure S2: Directed acyclic graph for E. coli contamination in stored water

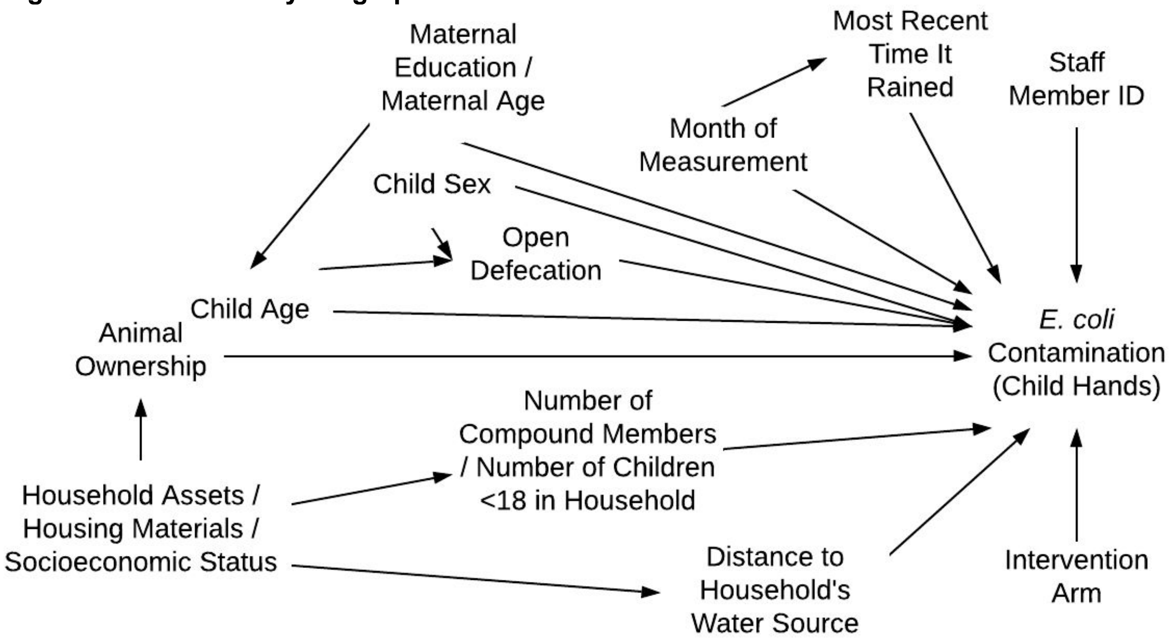

Figure S3: Directed acyclic graph for E. coli contamination on child hands

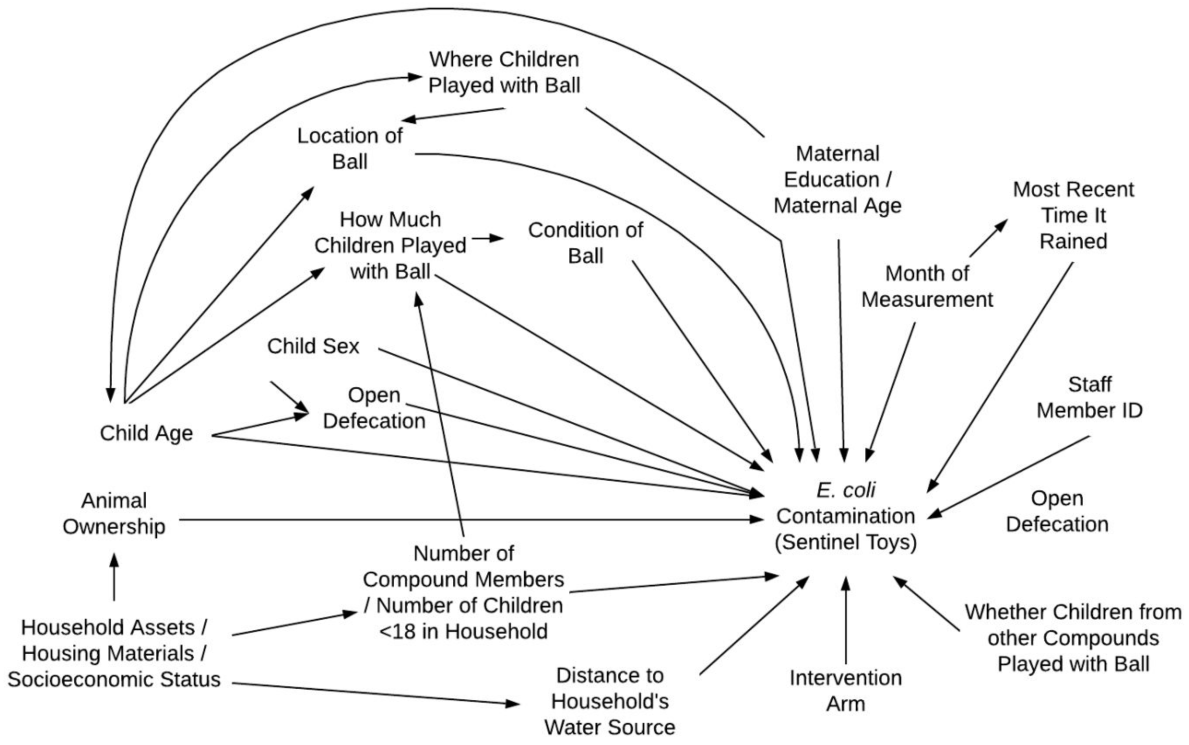

Figure S4: Directed acyclic graph for E. coli contamination on sentinel toys

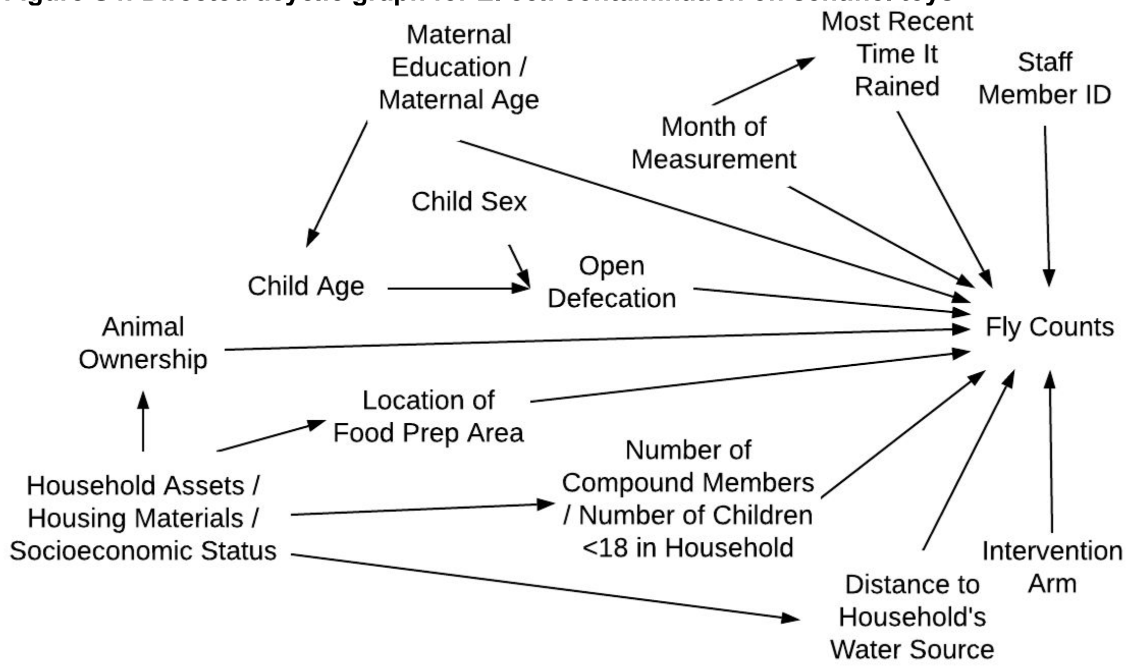

Figure S5: Directed acyclic graph for fly counts

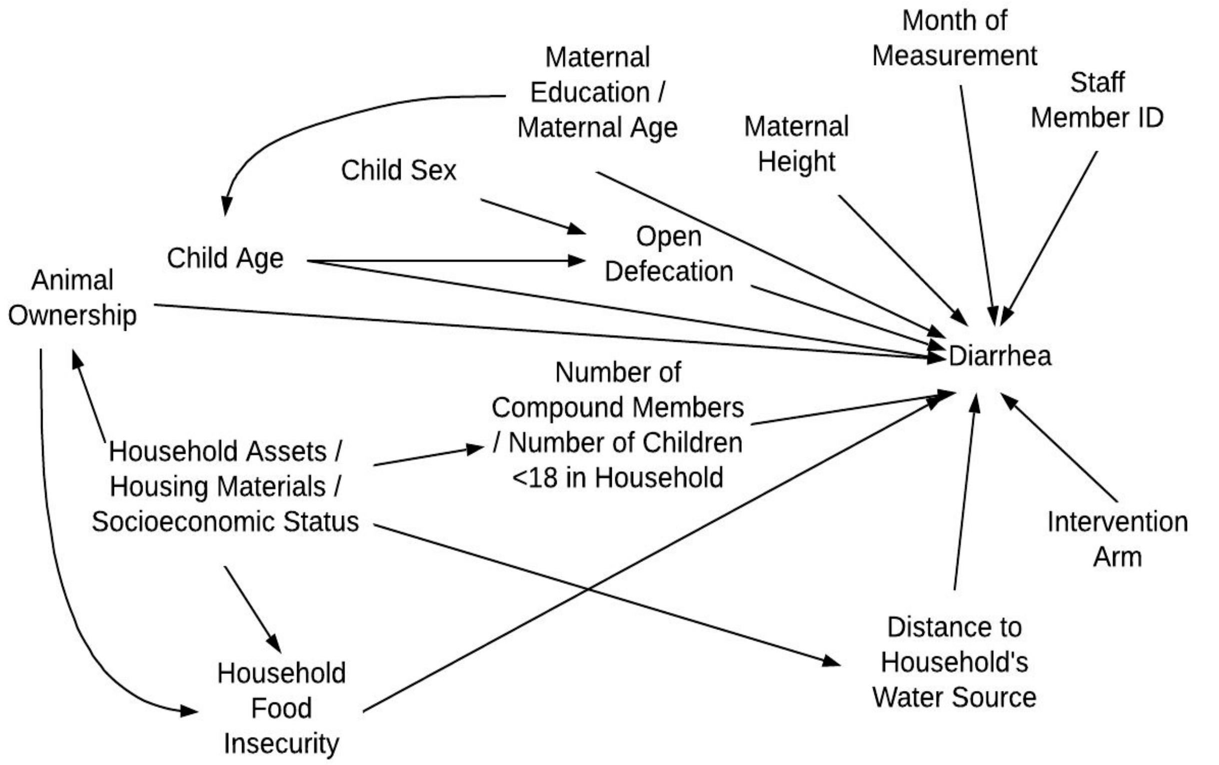

Figure S6: Directed acyclic graph for diarrhea

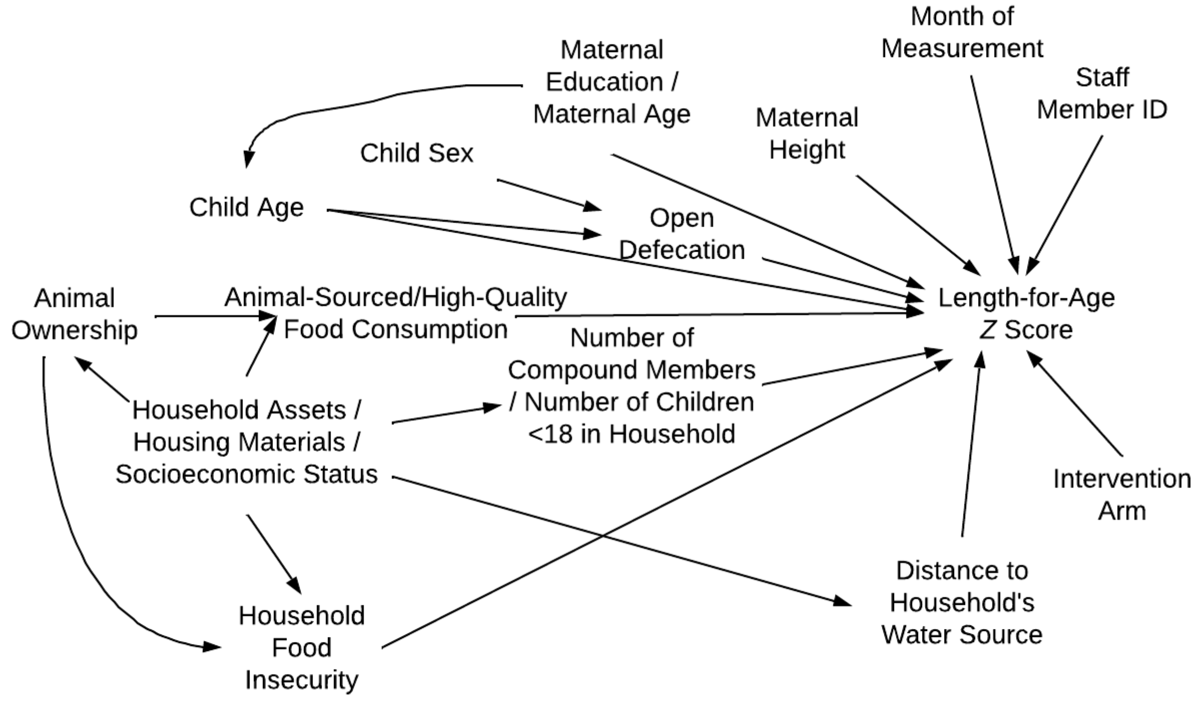

Figure S7: Directed acyclic graph for length-for-age Z-scores

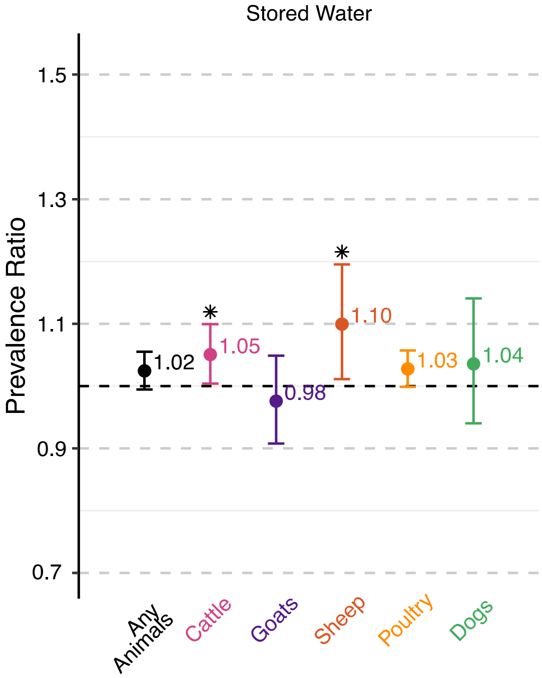

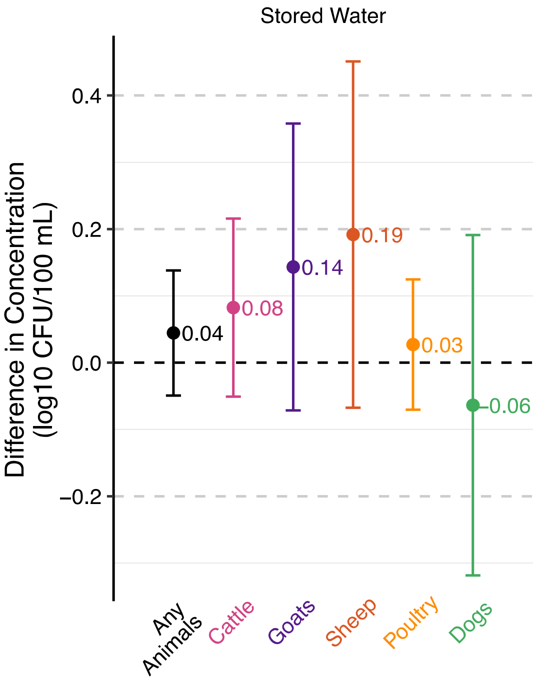

Figure S8: Associations between $\log_{10}$ number of animals owned by households and stored water contamination among households with and without detectable free chlorine. Prevalence ratios (top, N: 2014, Prevalence: 91.8) and differences in $\log_{10}$ concentrations (bottom, N: 2012, Log10 Mean: 1.47) for E. coli in stored water for each additional $\log_{10}$ animal owned, adjusting for the presence of detectable free chlorine in stored water in addition to other covariates listed in Supporting Information 1. Asterisks indicate significance at a significance level of 0.05.

Table S1: E. coli measured at one- and two-year assessments, combined and separately, interventions vs. control.

One- and Two-Year Assessments Combined

				Prevalence Ratio						Log10 Difference					
				Unadjusted			Adjusted			Unadjusted			Adjusted		
Arms	N	Prev.	Log10 Mean (CFU/ 100 mL)	Prev. Ratio	95% Confidence Intervals		Prev. Ratio	95% Confidence Intervals		Log10 Diff.	95% Confidence Intervals		Log10 Diff.	95% Confidence Intervals	
**Stored Water**															
Control/Nutrition	1376	0.94	1.48	-	-	-	-	-	-	-	-	-	-	-	-
WSH/WSH+Nutrition	1231	0.67	0.96	0.70	0.67	0.74	0.70	0.66	0.74	-0.53	-0.63	-0.43	-0.53	-0.63	-0.43
Water	635	0.68	0.97	0.73	0.68	0.78	0.72	0.68	0.78	-0.51	-0.62	-0.40	-0.49	-0.61	-0.38
Handwashing	667	0.93	1.44	0.98	0.95	1.00	0.98	0.95	1.00	-0.06	-0.16	0.03	-0.10	-0.20	-0.01
**Child Hands**															
Control/Nutrition	1378	0.90	1.74	-	-	-	-	-	-	-	-	-	-	-	-
WSH/WSH+Nutrition	1313	0.91	1.80	1.00	0.98	1.03	1.00	0.98	1.03	0.04	-0.04	0.13	0.08	-0.01	0.17
**Sentinel Toys**															
Control/Nutrition	1276	0.73	0.58	-	-	-	-	-	-	-	-	-	-	-	-
WSH/WSH+Nutrition	1146	0.72	0.62	0.97	0.93	1.02	0.97	0.91	1.03	0.02	-0.07	0.10	-0.02	-0.11	0.07

One-Year Assessment

				Prevalence Ratio						Log10 Difference					
				Unadjusted			Adjusted			Unadjusted			Adjusted		
Arms	N	Prev.	Log10 Mean (CFU/ 100 mL)	Prev. Ratio	95% Confidence Intervals		Prev. Ratio	95% Confidence Intervals		Log10 Diff.	95% Confidence Intervals		Log10 Diff.	95% Confidence Intervals	
**Stored Water**															
Control/Nutrition	550	0.94	1.49	-	-	-	-	-	-	-	-	-	-	-	-
WSH/WSH+Nutrition	504	0.52	0.63	0.55	0.48	0.61	0.54	0.48	0.61	-0.90	-1.07	-0.74	-0.87	-1.03	-0.70
Water	251	0.62	0.83	0.66	0.58	0.76	0.65	0.57	0.75	-0.64	-0.86	-0.43	-0.66	-0.89	-0.42
Handwashing	275	0.95	1.45	1.01	0.97	1.05	1.02	0.98	1.05	-0.05	-0.22	0.12	-0.08	-0.26	0.09
**Child Hands**															
Control/Nutrition	518	0.91	1.78	-	-	-	-	-	-	-	-	-	-	-	-
WSH/WSH+Nutrition	520	0.91	1.79	1.00	0.95	1.04	1.00	0.95	1.04	0.00	-0.16	0.17	0.07	-0.10	0.23
**Sentinel Toys**															
Control/Nutrition	687	0.79	0.66	-	-	-	-	-	-	-	-	-	-	-	-
WSH/WSH+Nutrition	628	0.79	0.74	0.98	0.93	1.04	0.98	0.89	1.07	0.06	-0.08	0.19	0.02	-0.13	0.16

Two-Year Assessment

				Prevalence Ratio						Log10 Difference					
				Unadjusted			Adjusted			Unadjusted			Adjusted		
Arms	N	Prev.	Log10 Mean (CFU/ 100 mL)	Prev. Ratio	95% Confidence Intervals		Prev. Ratio	95% Confidence Intervals		Log10 Diff.	95% Confidence Intervals		Log10 Diff.	95% Confidence Intervals	
**Stored Water**															
Control/Nutrition	826	0.94	1.48	-	-	-	-	-	-	-	-	-	-	-	-
WSH/WSH+Nutrition	727	0.76	1.19	0.81	0.76	0.85	0.81	0.76	0.85	-0.29	-0.40	-0.18	-0.30	-0.41	-0.19
Water	384	0.72	1.07	0.76	0.72	0.81	0.77	0.72	0.82	-0.43	-0.54	-0.32	-0.37	-0.49	-0.25
Handwashing	392	0.91	1.42	0.95	0.92	0.99	0.95	0.92	0.98	-0.07	-0.19	0.05	-0.10	-0.22	0.03
**Child Hands**															
Control/Nutrition	860	0.90	1.71	-	-	-	-	-	-	-	-	-	-	-	-
WSH/WSH+Nutrition	793	0.92	1.80	1.01	0.98	1.04	1.01	0.98	1.04	0.07	-0.05	0.20	0.13	0.00	0.26
**Sentinel Toys**															
Control/Nutrition	589	0.66	0.49	-	-	-	-	-	-	-	-	-	-	-	-
WSH/WSH+Nutrition	518	0.64	0.48	0.96	0.88	1.04	0.97	0.89	1.07	-0.04	-0.15	0.07	-0.04	-0.15	0.06

Table S2: Total coliforms (water and hand samples) or fecal coliforms (toy samples) measured at one- and two-year assessments, combined and separately, interventions vs. control.

One- and Two-Year Assessments Combined

				Prevalence Ratio						Log10 Difference					
				Unadjusted			Adjusted			Unadjusted			Adjusted		
Arms	N	Prev.	Log10 Mean (CFU/ 100 mL)	Prev. Ratio	95% Confidence Intervals		Prev. Ratio	95% Confidence Intervals		Log10 Diff.	95% Confidence Intervals		Log10 Diff.	95% Confidence Intervals	
**Stored Water**															
Control/Nutrition	1376	0.99	2.56	-	-	-	-	-	-	-	-	-	-	-	-
WSH/WSH+Nutrition	1231	0.81	1.80	0.82	0.79	0.85	0.82	0.79	0.85	-0.76	-0.85	-0.67	-0.75	-0.83	-0.66
Water	635	0.84	1.85	0.85	0.82	0.89	0.85	0.82	0.89	-0.69	-0.83	-0.56	-0.66	-0.79	-0.54
Handwashing	664	0.98	2.52	0.99	0.98	1.00	0.99	0.98	1.01	-0.03	-0.09	0.03	-0.03	-0.09	0.02
**Child Hands**															
Control/Nutrition	1376	0.99	2.71	-	-	-	-	-	-	-	-	-	-	-	-
WSH/WSH+Nutrition	1312	0.99	2.72	1.00	0.99	1.01	1.00	0.99	1.01	-0.01	-0.05	0.04	-0.01	-0.06	0.04
**Sentinel Toys**															
Control/Nutrition	1276	0.90	1.23	-	-	-	-	-	-	-	-	-	-	-	-
WSH/WSH+Nutrition	1146	0.90	1.25	0.98	0.96	1.01	0.98	0.94	1.02	-0.01	-0.09	0.07	-0.02	-0.11	0.07

One-Year Assessment

				Prevalence Ratio						Log10 Difference					
				Unadjusted			Adjusted			Unadjusted			Adjusted		
Arms	N	Prev.	Log10 Mean (CFU/ 100 mL)	Prev. Ratio	95% Confidence Intervals		Prev. Ratio	95% Confidence Intervals		Log10 Diff.	95% Confidence Intervals		Log10 Diff.	95% Confidence Intervals	
**Stored Water**															
Control/Nutrition	550	0.99	2.54	-	-	-	-	-	-	-	-	-	-	-	-
WSH/WSH+Nutrition	504	0.71	1.34	0.71	0.67	0.77	0.71	0.67	0.77	-1.22	-1.36	-1.08	-1.17	-1.31	-1.03
Water	251	0.82	1.66	0.82	0.77	0.87	0.82	0.76	0.87	-0.87	-1.09	-0.65	-0.86	-1.10	-0.63
Handwashing	272	0.98	2.50	0.99	0.97	1.01	0.99	0.97	1.01	-0.03	-0.10	0.04	-0.01	-0.09	0.07
**Child Hands**															
Control/Nutrition	516	0.99	2.63	-	-	-	-	-	-	-	-	-	-	-	-
WSH/WSH+Nutrition	520	0.99	2.62	1.00	0.98	1.01	1.00	0.98	1.01	-0.02	-0.10	0.06	-0.01	-0.08	0.07
**Sentinel Toys**															
Control/Nutrition	687	0.93	1.36	-	-	-	-	-	-	-	-	-	-	-	-
WSH/WSH+Nutrition	628	0.93	1.38	0.98	0.94	1.02	0.97	0.92	1.03	-0.01	-0.14	0.12	0.03	-0.12	0.18

Two-Year Assessment

				Prevalence Ratio						Log10 Difference					
				Unadjusted			Adjusted			Unadjusted			Adjusted		
Arms	N	Prev.	Log10 Mean (CFU/ 100 mL)	Prev. Ratio	95% Confidence Intervals		Prev. Ratio	95% Confidence Intervals		Log10 Diff.	95% Confidence Intervals		Log10 Diff.	95% Confidence Intervals	
**Stored Water**															
Control/Nutrition	826	0.99	2.57	-	-	-	-	-	-	-	-	-	-	-	-
WSH/WSH+Nutrition	727	0.88	2.11	0.89	0.86	0.92	0.89	0.86	0.92	-0.46	-0.56	-0.36	-0.46	-0.57	-0.36
Water	384	0.86	1.97	0.87	0.83	0.92	0.87	0.83	0.92	-0.59	-0.74	-0.43	-0.55	-0.71	-0.39
Handwashing	392	0.99	2.54	1.00	0.98	1.01	1.00	0.98	1.01	-0.04	-0.13	0.04	-0.04	-0.12	0.04
**Child Hands**															
Control/Nutrition	860	0.99	2.75	-	-	-	-	-	-	-	-	-	-	-	-
WSH/WSH+Nutrition	792	1.00	2.78	1.00	1.00	1.01	1.00	1.00	1.01	0.00	-0.05	0.05	0.01	-0.05	0.06
**Sentinel Toys**															
Control/Nutrition	589	0.86	1.07	-	-	-	-	-	-	-	-	-	-	-	-
WSH/WSH+Nutrition	518	0.86	1.10	0.99	0.94	1.04	0.98	0.93	1.05	-0.02	-0.14	0.10	-0.06	-0.18	0.06

Table S3: Fly counts and dirt on hands/fingernails measured at one- and two-year assessments, combined and separately, interventions vs. control.

				One- and Two-Year Assessments Combined											
				Prevalence Ratio						Fly Count Difference					
				Unadjusted			Adjusted			Unadjusted			Adjusted		
Arms	N	Prev.	Mean	Prev. Ratio	95% Confidence Intervals		Prev. Ratio	95% Confidence Intervals		Fly Diff.	95% Confidence Intervals		Fly Diff.	95% Confidence Intervals	
**Flies Caught in Food Preparation Area**															
Control/Nutrition	1882	0.62	3.37	-	-	-	-	-	-	-	-	-	-	-	-
WSH/WSH+Nutrition	1386	0.62	3.26	1.01	0.94	1.08	1.00	0.94	1.07	-0.12	-0.60	0.36	-0.22	-0.70	0.26
Sanitation	1001	0.641	3.25	0.99	0.93	1.06	0.98	0.92	1.05	-0.30	-0.88	0.27	-0.23	-0.78	0.31
**Flies Caught in Latrine Area**															
Control/Nutrition	1793	0.67	3.72	-	-	-	-	-	-	-	-	-	-	-	-
WSH/WSH+Nutrition	1354	0.64	3.19	0.96	0.91	1.01	0.96	0.91	1.01	-0.49	-0.87	-0.11	-0.52	-0.89	-0.15
Sanitation	971	0.70	3.52	1.02	0.96	1.08	1.01	0.96	1.07	-0.49	-1.00	0.01	-0.42	-0.95	0.10
**Visible Dirt on Caregiver Hands**															
Control/Nutrition	3605	0.26	-	-	-	-	-	-	-	-	-	-	-	-	-
WSH/WSH+Nutrition	2442	0.23	-	0.86	0.78	0.95	0.86	0.75	0.98	-	-	-	-	-	-
**Visible Dirt under Caregiver Fingernails**															
Control/Nutrition	3605	0.54	-	-	-	-	-	-	-	-	-	-	-	-	-
WSH/WSH+Nutrition	2442	0.48	-	0.90	0.85	0.96	0.91	0.84	0.98	-	-	-	-	-	-
**Visible Dirt on Child Hands**															
Control/Nutrition	3390	0.36	-	-	-	-	-	-	-	-	-	-	-	-	-
WSH/WSH+Nutrition	2288	0.34	-	0.92	0.85	1.01	0.87	0.78	0.97	-	-	-	-	-	-
**Visible Dirt under Child Fingernails**															
Control/Nutrition	3394	0.67	-	-	-	-	-	-	-	-	-	-	-	-	-
WSH/WSH+Nutrition	2290	0.66	-	0.99	0.94	1.03	0.95	0.89	1.02	-	-	-	-	-	-

				One-Year Assessment											
				Prevalence Ratio						Fly Count Difference					
				Unadjusted			Adjusted			Unadjusted			Adjusted		
Arms	N	Prev.	Mean	Prev. Ratio	95% Confidence Intervals		Prev. Ratio	95% Confidence Intervals		Fly Diff.	95% Confidence Intervals		Fly Diff.	95% Confidence Intervals	
**Flies Caught in Food Preparation Area**															
Control/Nutrition	562	0.65	3.80	-	-	-	-	-	-	-	-	-	-	-	-
WSH/WSH+Nutrition	590	0.65	3.68	0.97	0.89	1.06	0.98	0.89	1.07	-0.28	-1.18	0.63	-0.24	-1.17	0.69
Sanitation	395	0.64	3.30	0.94	0.81	1.10	0.93	0.79	1.09	-0.42	-1.50	0.66	-0.69	-1.87	0.49
**Flies Caught in Latrine Area**															
Control/Nutrition	535	0.66	3.53	-	-	-	-	-	-	-	-	-	-	-	-
WSH/WSH+Nutrition	578	0.59	2.84	0.88	0.79	0.97	0.86	0.77	0.95	-0.59	-1.16	-0.02	-0.66	-1.24	-0.08
Sanitation	384	0.69	3.25	0.97	0.84	1.12	0.94	0.81	1.10	-0.18	-0.92	0.57	-0.55	-1.43	0.33
**Visible Dirt on Caregiver Hands**															
Control/Nutrition	1494	0.26	-	-	-	-	-	-	-	-	-	-	-	-	-
WSH/WSH+Nutrition	1027	0.21	-	0.81	0.70	0.93	0.91	0.75	1.11	-	-	-	-	-	-
**Visible Dirt under Caregiver Fingernails**															
Control/Nutrition	1494	0.47	-	-	-	-	-	-	-	-	-	-	-	-	-
WSH/WSH+Nutrition	1027	0.40	-	0.87	0.79	0.96	0.91	0.80	1.05	-	-	-	-	-	-
**Visible Dirt on Child Hands**															
Control/Nutrition	1285	0.31	-	-	-	-	-	-	-	-	-	-	-	-	-
WSH/WSH+Nutrition	882	0.28	-	0.87	0.74	1.03	0.79	0.61	1.02	-	-	-	-	-	-
**Visible Dirt under Child Fingernails**															
Control/Nutrition	1289	0.57	-	-	-	-	-	-	-	-	-	-	-	-	-
WSH/WSH+Nutrition	884	0.56	-	1.00	0.91	1.09	1.01	0.89	1.15	-	-	-	-	-	-

				Two-Year Assessment											
				Prevalence Ratio						Fly Count Difference					
				Unadjusted			Adjusted			Unadjusted			Adjusted		
**Arms**	**N**	**Prev.**	**Mean**	**Prev. Ratio**	**95% Confidence Intervals**		**Prev. Ratio**	**95% Confidence Intervals**		**Fly Diff.**	**95% Confidence Intervals**		**Fly Diff.**	**95% Confidence Intervals**	
**Flies Caught in Food Preparation Area**															
Control/Nutrition	1320	0.60	3.18	-	-	-	-	-	-	-	-	-	-	-	-
WSH/WSH+Nutrition	796	0.60	2.95	1.01	0.93	1.10	1.01	0.93	1.10	-0.20	-0.72	0.33	-0.16	-0.67	0.35
Sanitation	606	0.64	3.21	1.01	0.93	1.09	1.01	0.93	1.09	-0.24	-0.95	0.47	-0.02	-0.64	0.60
**Flies Caught in Latrine Area**															
Control/Nutrition	1258	0.67	3.80	-	-	-	-	-	-	-	-	-	-	-	-
WSH/WSH+Nutrition	776	0.67	3.45	1.02	0.96	1.08	1.01	0.95	1.08	-0.34	-0.78	0.10	-0.48	-0.91	-0.06
Sanitation	587	0.71	3.69	1.04	0.96	1.13	1.03	0.95	1.11	-0.46	-1.09	0.16	-0.56	-1.17	0.05
**Visible Dirt on Caregiver Hands**															
Control/Nutrition	2111	0.26	-	-	-	-	-	-	-	-	-	-	-	-	-
WSH/WSH+Nutrition	1415	0.24	-	0.90	0.79	1.02	0.84	0.69	1.01	-	-	-	-	-	-
**Visible Dirt under Caregiver Fingernails**															
Control/Nutrition	2111	0.58	-	-	-	-	-	-	-	-	-	-	-	-	-
WSH/WSH+Nutrition	1415	0.54	-	0.92	0.87	0.99	0.91	0.83	1.00	-	-	-	-	-	-
**Visible Dirt on Child Hands**															
Control/Nutrition	2105	0.39	-	-	-	-	-	-	-	-	-	-	-	-	-
WSH/WSH+Nutrition	1406	0.37	-	0.95	0.86	1.04	0.91	0.79	1.05	-	-	-	-	-	-
**Visible Dirt under Child Fingernails**															
Control/Nutrition	2105	0.74	-	-	-	-	-	-	-	-	-	-	-	-	-
WSH/WSH+Nutrition	1406	0.72	-	0.98	0.93	1.03	0.94	0.88	1.01	-	-	-	-	-	-

Table S4: Fly counts and dirt on hands/fingernails measured at one- and two-year assessments, combined and separately, interventions vs. control.

Arms	N	Prev.	Prev. Ratio	95% Confidence Intervals	
**Flies Caught in Food Preparation Area**					
House Flies					
Control/Nutrition	1881	0.60	-	-	-
WSH/WSH+Nutrition	1384	0.61	1.01	0.94	1.07
Sanitation	998	0.63	0.98	0.92	1.06
Bottle Flies					
Control/Nutrition	1881	0.03	-	-	-
WSH/WSH+Nutrition	1384	0.03	1.00	0.64	1.56
Sanitation	998	0.04	1.44	0.94	2.21
Flesh Flies					
Control/Nutrition	1881	0.00	-	-	-
WSH/WSH+Nutrition	1384	0.01	1.61	0.62	4.19
Sanitation	998	0.01	1.75	0.77	4.00
**Flies Caught in Latrine Area**					
House Flies					
Control/Nutrition	1793	0.30	-	-	-
WSH/WSH+Nutrition	1352	0.26	0.87	0.75	1.01
Sanitation	971	0.27	0.90	0.77	1.06
Bottle Flies					
Control/Nutrition	1793	0.55	-	-	-
WSH/WSH+Nutrition	1352	0.54	0.96	0.89	1.02
Sanitation	971	0.60	1.04	0.97	1.12
Flesh Flies					
Control/Nutrition	1793	0.03	-	-	-
WSH/WSH+Nutrition	1352	0.02	0.81	0.50	1.33
Sanitation	971	0.03	1.01	0.57	1.80

Table S5: E. coli and fly prevalence and concentration measured at one- and two-year assessments, combined and separately, interventions vs. control, subgroup analysis by season.

One- and Two-Year Assessments Combined																						
	Prevalence											Concentration										
	Wet Season					Dry Season						Wet Season					Dry Season					
Arms	N	Prev.	Prev. Ratio	95% Confidence Intervals		N	Prev.	Prev. Ratio	95% Confidence Intervals		Inter-action P-Value	N	Log10 Mean (CFU/ 100 mL)/ Mean (Flies)	Log Diff./ Fly Count	95% Confidence Intervals		N	Log10 Mean (CFU/ 100 mL)/ Mean (Flies)	Log Diff./ Fly Count	95% Confidence Intervals		Inter-action P-Value
**Stored Water**																						
Control/Nutrition	737	0.94	-	-	-	637	0.94	-	-	-	-	731	1.51	-	-	-	635	1.44	-	-	-	-
WSH/ WSH+Nutrition	665	0.65	0.69	0.64	0.75	565	0.68	0.72	0.67	0.77	0.544	664	0.95	-0.57	-0.70	-0.44	564	0.966	-0.47	-0.61	-0.34	0.282
Water	361	0.65	0.69	0.62	0.76	274	0.72	0.77	0.71	0.84	0.091	361	0.95	-0.57	-0.75	-0.39	274	1.003	-0.44	-0.56	-0.32	0.262
Handwashing	363	0.93	0.98	0.94	1.02	304	0.92	0.97	0.93	1.01	0.834	362	1.47	-0.05	-0.18	0.08	304	1.395	-0.07	-0.21	0.07	0.812
**Child Hands**																						
Control/Nutrition	743	0.89	-	-	-	632	0.92	-	-	-	-	724	1.68	-	-	-	623	1.81	-	-	-	-
WSH/ WSH+Nutrition	714	0.89	0.99	0.96	1.03	597	0.93	1.02	0.99	1.04	0.370	705	1.71	-0.02	-0.13	0.10	593	1.905	0.13	-0.01	0.26	0.127
**Sentinel Toys**																						
Control/Nutrition	627	0.72	-	-	-	649	0.74	-	-	-	-	616	0.59	-	-	-	644	0.57	-	-	-	-
WSH/ WSH+Nutrition	591	0.73	0.99	0.93	1.04	555	0.71	0.96	0.89	1.03	0.534	587	0.67	0.02	-0.07	0.11	552	0.57	0.01	-0.13	0.15	0.873
**Flies Caught in Food Preparation Area**																						
Control/Nutrition	946	0.65	-	-	-	936	0.59	-	-	-	-	946	3.78	-	-	-	936	2.95	-	-	-	-
WSH/ WSH+Nutrition	745	0.62	0.98	0.91	1.05	641	0.62	1.03	0.93	1.14	0.396	745	3.35	-0.54	-1.26	0.18	641	3.15	0.24	-0.28	0.76	0.075
Sanitation	527	0.68	1.00	0.92	1.09	474	0.60	0.97	0.87	1.09	0.724	527	3.63	-0.48	-1.35	0.38	474	2.82	-0.11	-0.72	0.49	0.462
**Flies Caught in Latrine Area**																						
Control/Nutrition	912	0.70	-	-	-	881	0.63	-	-	-	-	912	4.17	-	-	-	881	3.25	-	-	-	-
WSH/ WSH+Nutrition	731	0.64	0.93	0.87	1.00	623	0.63	0.97	0.89	1.07	0.488	731	3.20	-0.82	-1.29	-0.34	623	3.17	-0.22	-0.78	0.34	0.111
Sanitation	515	0.70	0.96	0.89	1.04	456	0.69	1.09	1.00	1.18	0.041	515	3.74	-0.84	-1.58	-0.09	456	3.27	-0.12	-0.70	0.46	0.110

One-Year Assessment																						
	Prevalence											Concentration										
	Wet Season					Dry Season						Wet Season					Dry Season					
Arms	N	Prev.	Prev. Ratio	95% Confidence Intervals		N	Prev.	Prev. Ratio	95% Confidence Intervals		Inter-action P-Value	N	Log10 Mean (CFU/ 100 mL)/ Mean (Flies)	Log Diff./ Fly Count	95% Confidence Intervals		N	Log10 Mean (CFU/ 100 mL)/ Mean (Flies)	Log Diff./ Fly Count	95% Confidence Intervals		Inter-action P-Value
**Stored Water**																						
Control/Nutrition	313	0.95	-	-	-	235	0.93	-	-	-	-	311	1.59	-	-	-	233	1.35	-	-	-	-
WSH/ WSH+Nutrition	301	0.56	0.59	0.51	0.69	86	0.64	0.49	0.41	0.58	0.086	300	0.73	-0.86	-1.09	-0.63	202	0.478	-0.93	-1.16	-0.71	0.658
Water	165	0.61	0.64	0.54	0.77	120	0.93	0.70	0.57	0.87	0.519	165	0.85	-0.72	-1.02	-0.43	86	0.795	-0.51	-0.78	-0.24	0.301
Handwashing	155	0.96	1.01	0.96	1.07	202	0.47	1.01	0.94	1.09	0.998	154	1.56	-0.06	-0.26	0.15	120	1.316	-0.03	-0.30	0.24	0.859
**Child Hands**																						
Control/Nutrition	295	0.93	-	-	-	220	0.89	-	-	-	-	285	1.88	-	-	-	218	1.64	-	-	-	-
WSH/ WSH+Nutrition	321	0.93	1.00	0.95	1.05	197	0.87	0.99	0.91	1.08	0.872	317	1.93	0.05	-0.13	0.22	196	1.562	-0.04	-0.34	0.27	0.639
**Sentinel Toys**																						
Control/Nutrition	327	0.77	-	-	-	360	0.81	-	-	-	-	321	0.72	-	-	-	357	0.60	-	-	-	-
WSH/ WSH+Nutrition	322	0.79	1.01	0.94	1.08	306	0.78	0.95	0.88	1.04	0.347	319	0.79	0.05	-0.10	0.19	303	0.69	0.07	-0.15	0.29	0.868
**Flies Caught in Food Preparation Area**																						
Control/Nutrition	304	0.69	-	-	-	231	0.63	-	-	-	-	314	4.43	-	-	-	248	3.00	-	-	-	-
WSH/ WSH+Nutrition	345	0.59	0.95	0.85	1.06	233	0.60	1.03	0.89	1.19	0.364	356	3.86	-0.83	-2.18	0.52	234	3.39	0.46	-0.72	1.64	0.169
Sanitation	203	0.68	0.96	0.79	1.18	181	0.69	0.91	0.71	1.18	0.759	207	3.58	-1.08	-2.51	0.35	188	2.99	0.52	-1.13	2.18	0.162
**Flies Caught in Latrine Area**																						
Control/Nutrition	304	0.69	-	-	-	231	0.63	-	-	-	-	304	3.90	-	-	-	231	3.04	-	-	-	-
WSH/ WSH+Nutrition	345	0.59	0.84	0.74	0.95	233	0.60	0.90	0.75	1.07	0.538	345	2.92	-0.96	-1.78	-0.15	233	2.74	-0.16	-1.10	0.78	0.250
Sanitation	203	0.68	0.94	0.78	1.12	181	0.69	1.02	0.82	1.28	0.542	203	3.62	-0.55	-1.59	0.49	181	2.83	0.36	-0.90	1.63	0.304

	Two-Year Assessment																					
	Prevalence											Concentration										
	Wet Season					Dry Season						Wet Season					Dry Season					
Arms	N	Prev.	Prev. Ratio	95% Confidence Intervals		N	Prev.	Prev. Ratio	95% Confidence Intervals		Inter-action P-Value	N	Log10 Mean (CFU/ 100 mL)/ Mean (Flies)	Log Diff./ Fly Count	95% Confidence Intervals		N	Log10 Mean (CFU/ 100 mL)/ Mean (Flies)	Log Diff./ Fly Count	95% Confidence Intervals		Inter-action P-Value
**Stored Water**																						
Control/Nutrition	424	0.94	-	-	-	402	0.95	-	-	-	-	420	1.46	-	-	-	402	1.50	-	-	-	-
WSH/ WSH+Nutrition	364	0.73	0.78	0.71	0.85	188	0.76	0.84	0.79	0.89	0.199	364	1.14	-0.32	-0.47	-0.17	362	1.238	-0.26	-0.39	-0.12	0.515
Water	196	0.68	0.72	0.65	0.80	184	0.91	0.81	0.74	0.88	0.099	196	1.04	-0.46	-0.62	-0.31	188	1.10	-0.39	-0.55	-0.23	0.530
Handwashing	208	0.90	0.95	0.91	1.00	363	0.80	0.95	0.91	0.99	0.913	208	1.40	-0.07	-0.25	0.112	184	1.446	-0.08	-0.23	0.08	0.968
**Child Hands**																						
Control/Nutrition	448	0.86	-	-	-	412	0.94	-	-	-	-	439	1.54	-	-	-	405	1.90	-	-	-	-
WSH/ WSH+Nutrition	393	0.87	0.99	0.94	1.05	400	0.97	1.02	0.99	1.05	0.356	388	1.53	-0.05	-0.21	0.122	397	2.075	0.19	0.02	0.37	0.059
**Sentinel Toys**																						
Control/Nutrition	300	0.66	-	-	-	289	0.66	-	-	-	-	295	0.46	-	-	-	287	0.53	-	-	-	-
WSH/ WSH+Nutrition	269	0.66	0.97	0.87	1.08	249	0.62	0.95	0.84	1.08	0.874	268	0.52	0.006	-0.14	0.149	249	0.43	-0.09	-0.25	0.07	0.381
**Flies Caught in Food Preparation Area**																						
Control/Nutrition	632	0.63	-	-	-	688	0.57	-	-	-	-	632	3.45	-	-	-	688	2.93	-	-	-	-
WSH/ WSH+Nutrition	389	0.60	0.98	0.88	1.09	407	0.61	1.04	0.93	1.17	0.405	389	2.89	-0.60	-1.49	0.30	407	3.01	0.17	-0.41	0.74	0.155
Sanitation	320	0.68	1.04	0.94	1.14	286	0.59	0.97	0.85	1.11	0.462	320	3.66	-0.26	-1.39	0.88	286	2.71	-0.22	-0.82	0.39	0.946
**Flies Caught in Latrine Area**																						
Control/Nutrition	608	0.71	-	-	-	650	0.63	-	-	-	-	608	4.30	-	-	-	650	3.33	-	-	-	-
WSH/ WSH+Nutrition	386	0.69	1.00	0.93	1.09	390	0.65	1.03	0.93	1.14	0.696	386	3.46	-0.61	-1.23	0.00	390	3.44	-0.10	-0.73	0.53	0.256
Sanitation	312	0.72	1.01	0.91	1.13	275	0.69	1.08	0.95	1.23	0.435	312	3.82	-0.83	-1.84	0.17	275	3.55	-0.08	-0.72	0.57	0.201

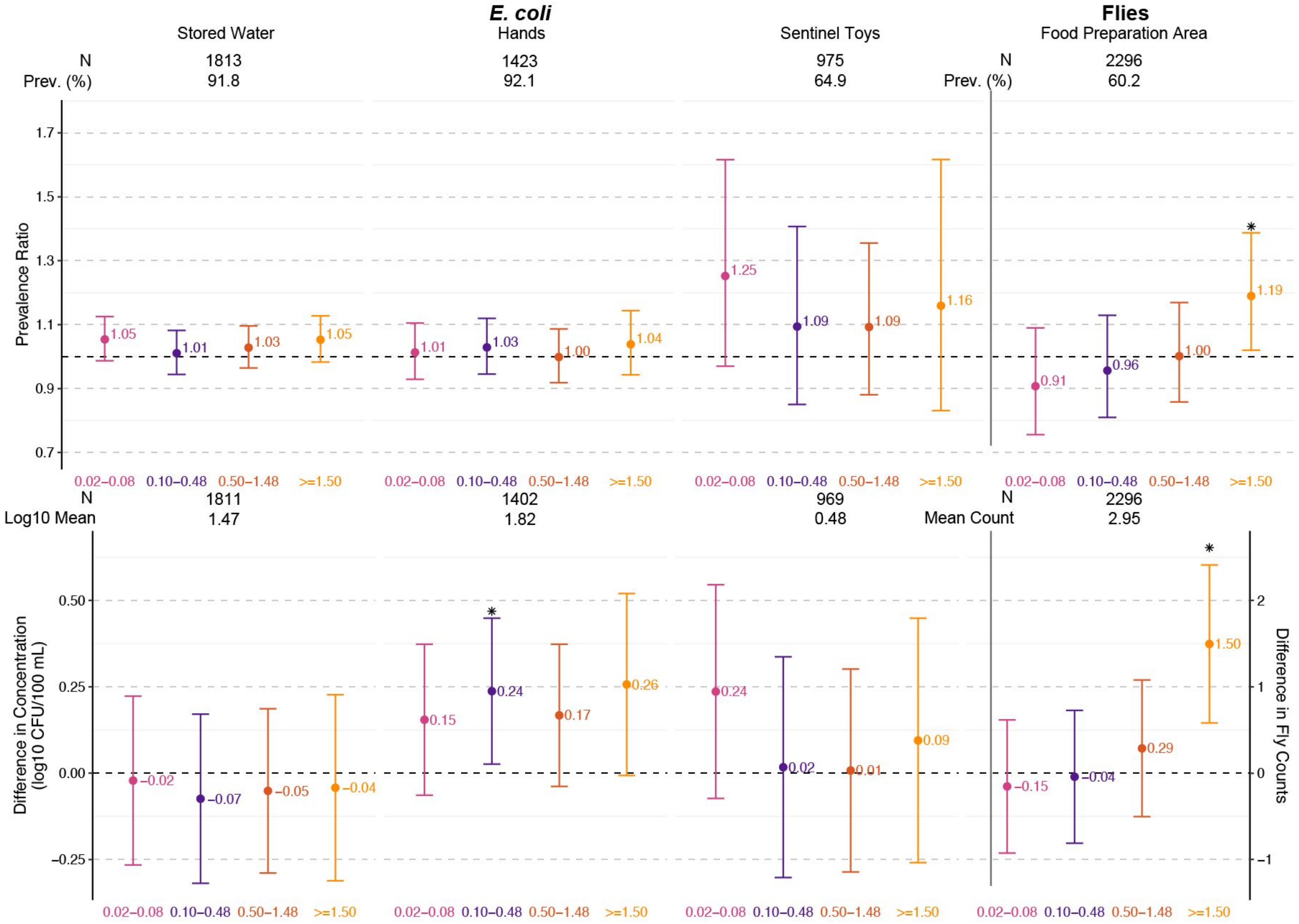

Figure S9: Associations between household TLU scores and environmental contamination. Prevalence ratios (top) and differences in log_{10} concentrations (bottom) for E. coli in stored water, on child hands, and on toys for each TLU category (left); prevalence ratios (top) and differences in fly counts (bottom) for flies at food preparation areas for each TLU category (right). The reference group is all non-animal-owning households (0 TLU). Asterisks indicate significance at a significance level of 0.05.

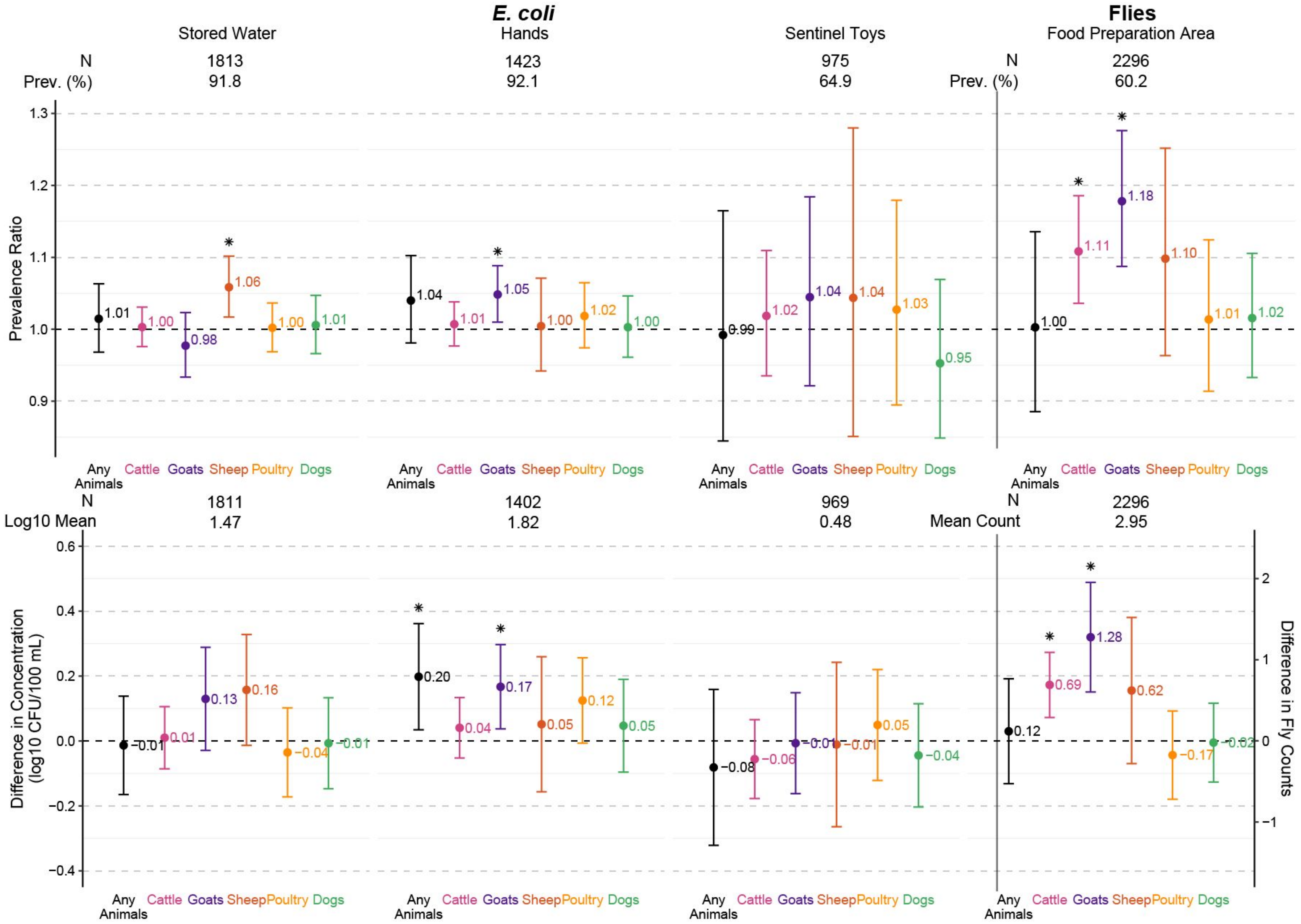

Figure S10: Associations between binary household animal ownership and environmental contamination. Prevalence ratios (top) and differences in $\log_{10}$ concentrations (bottom) for E. coli in stored water, on child hands, and on toys in animal-owning compared to non-animal-owning households (left); prevalence ratios (top) and differences in fly counts for flies at food preparation in animal-owning compared to non-animal-owning households (right). Asterisks indicate significance at a significance level of 0.05.

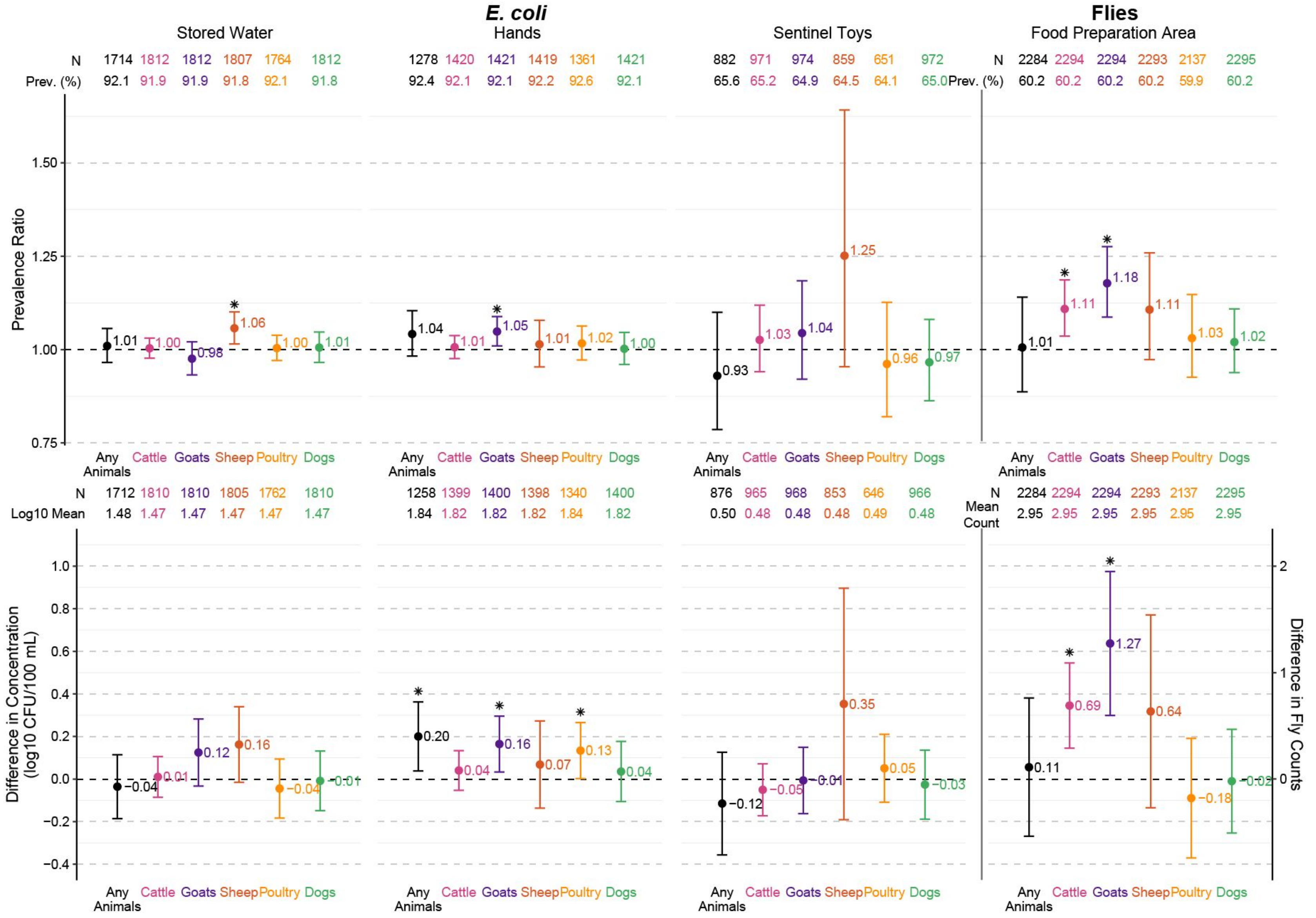

Figure S11: Associations between binary household animal ownership (households with extreme predicted probabilities excluded) and environmental contamination. Prevalence ratios (top) and differences in $\log_{10}$ concentrations (bottom) for E. coli in stored water, on child hands, and on toys in animal-owning compared to non-animal-owning households (left); prevalence ratios (top) and differences in fly counts for flies at food preparation in animal-owning compared to non-animal-owning households (right). Asterisks indicate significance at a significance level of 0.05.

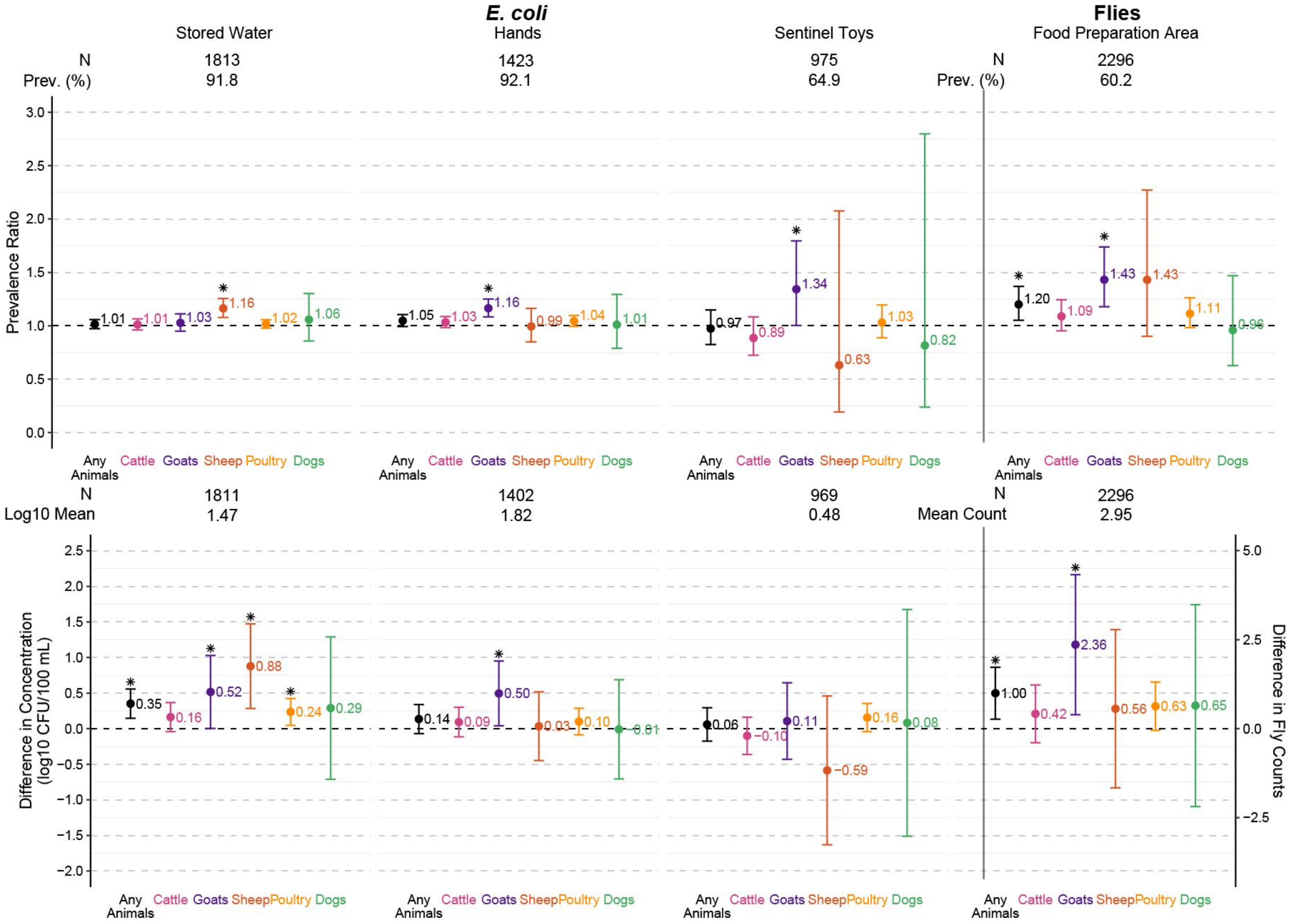

Figure S12: Associations between $\log_{10}$ median number of animals owned by households in the same village and environmental contamination. Prevalence ratios (top) and differences in $\log_{10}$ concentrations (bottom) for E. coli in stored water, on child hands, and on toys for each additional $\log_{10}$ animal owned (left); prevalence ratios (top) and differences in fly counts for flies at food preparation areas for each additional $\log_{10}$ animal owned (right). Asterisks indicate significance at a significance level of 0.05.

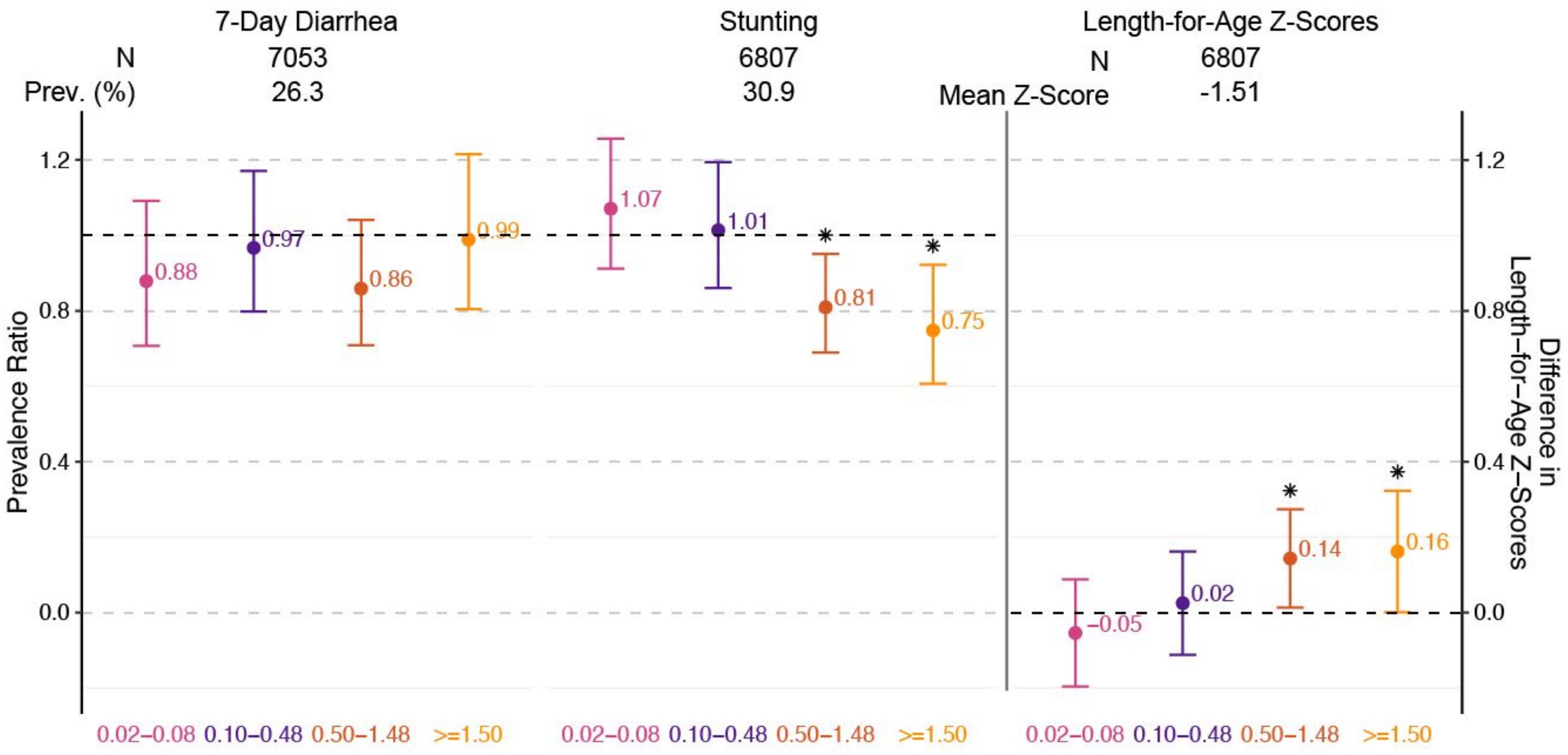

Figure S13: Associations between household TLU scores and child health. Prevalence ratios for diarrhea and stunting for each TLU category (left); differences in length-for-age Z-scores for each TLU category (right). Asterisks indicate significance at a significance level of 0.05.

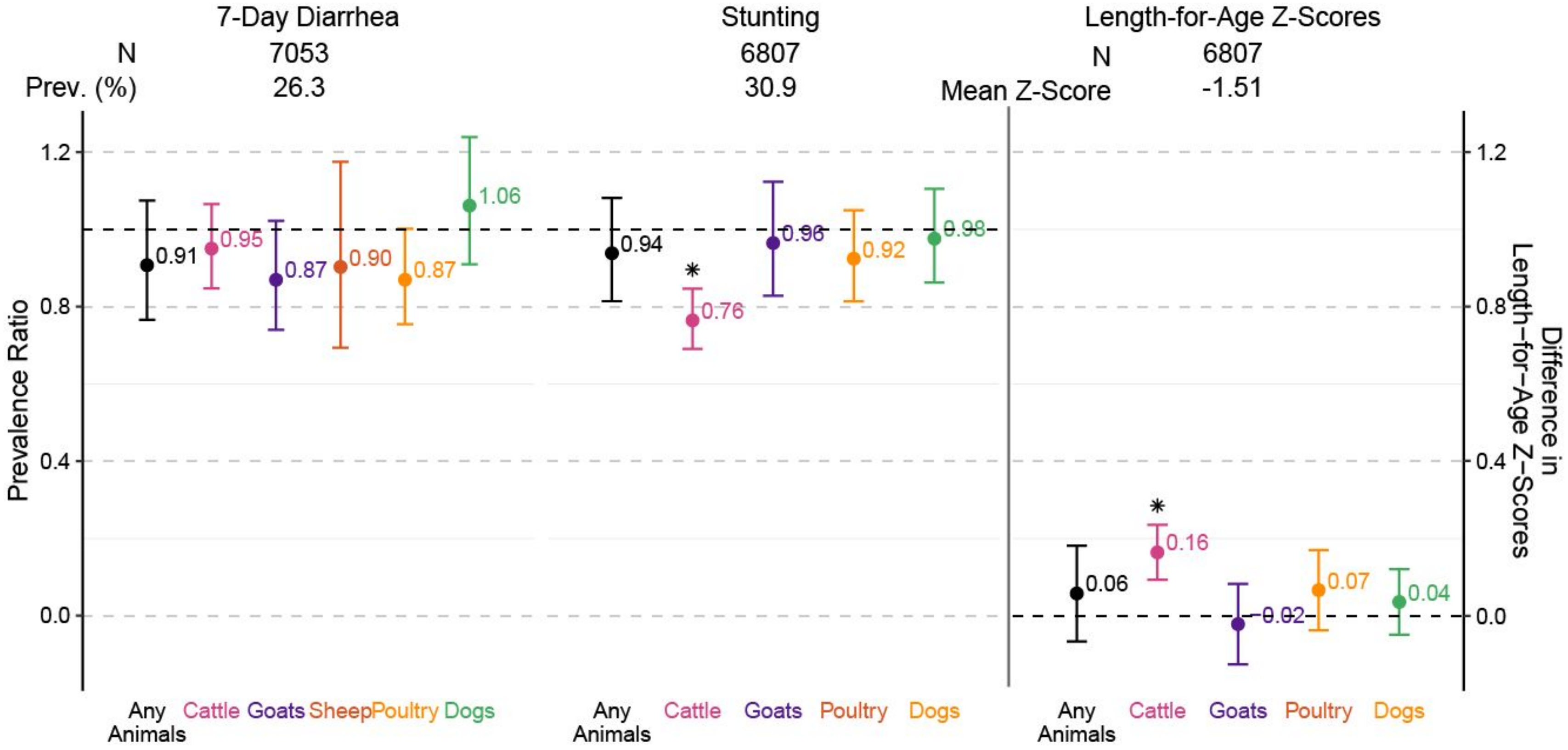

Figure S14: Associations between binary household animal ownership and child health. Prevalence ratios for diarrhea and stunting in animal-owning compared to non-animal-owning households (left); differences in length-for-age Z-scores in animal-owning compared to non-animal-owning households (right). Asterisks indicate significance at a significance level of 0.05.

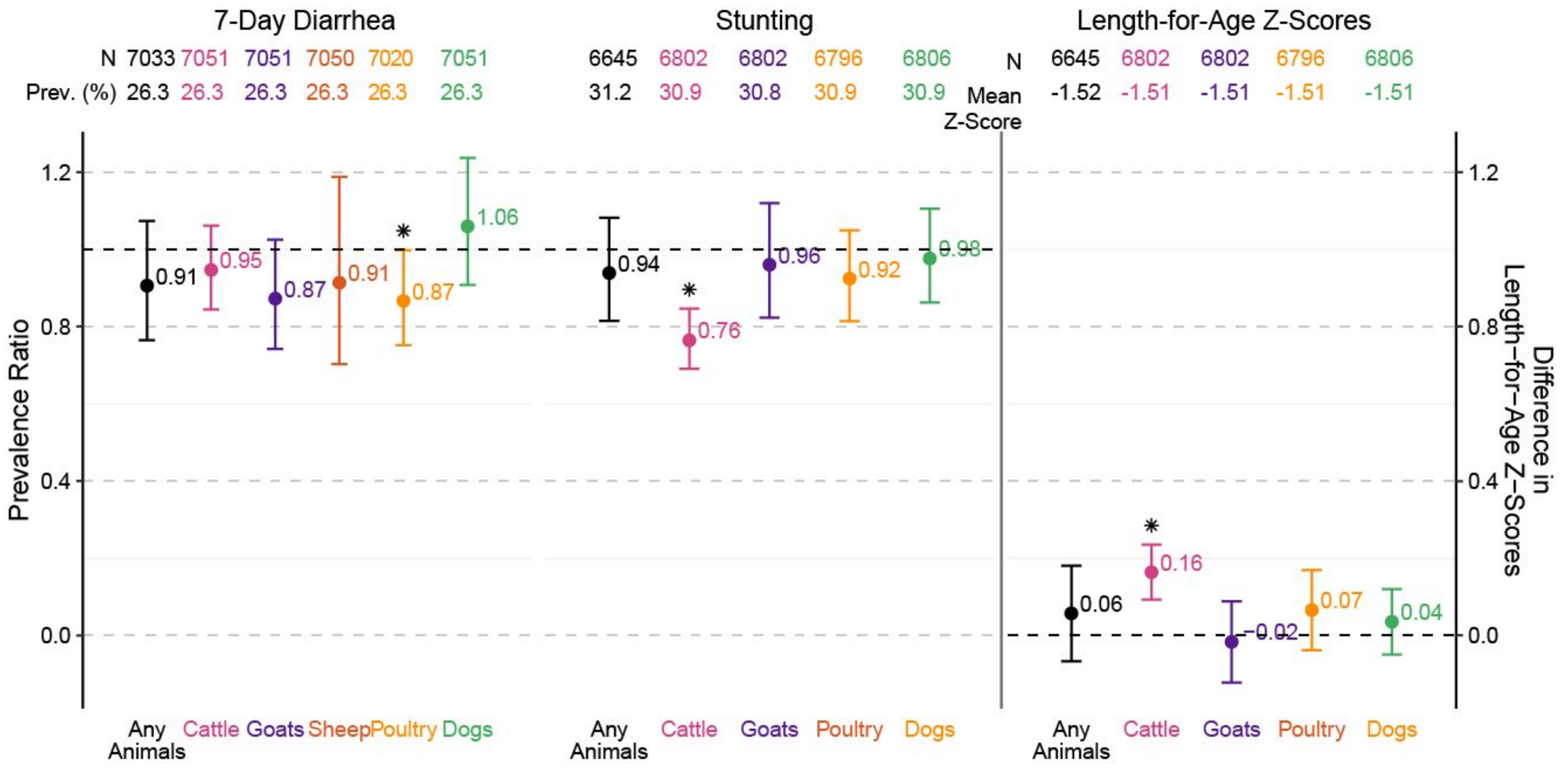

Figure S15: Associations between binary household animal ownership (households with extreme predicted probabilities excluded) and child health. Prevalence ratios for diarrhea and stunting in animal-owning compared to non-animal-owning households (left); differences in length-for-age Z-scores in animal-owning compared to non-animal-owning households (right). Asterisks indicate significance at a significance level of 0.05.

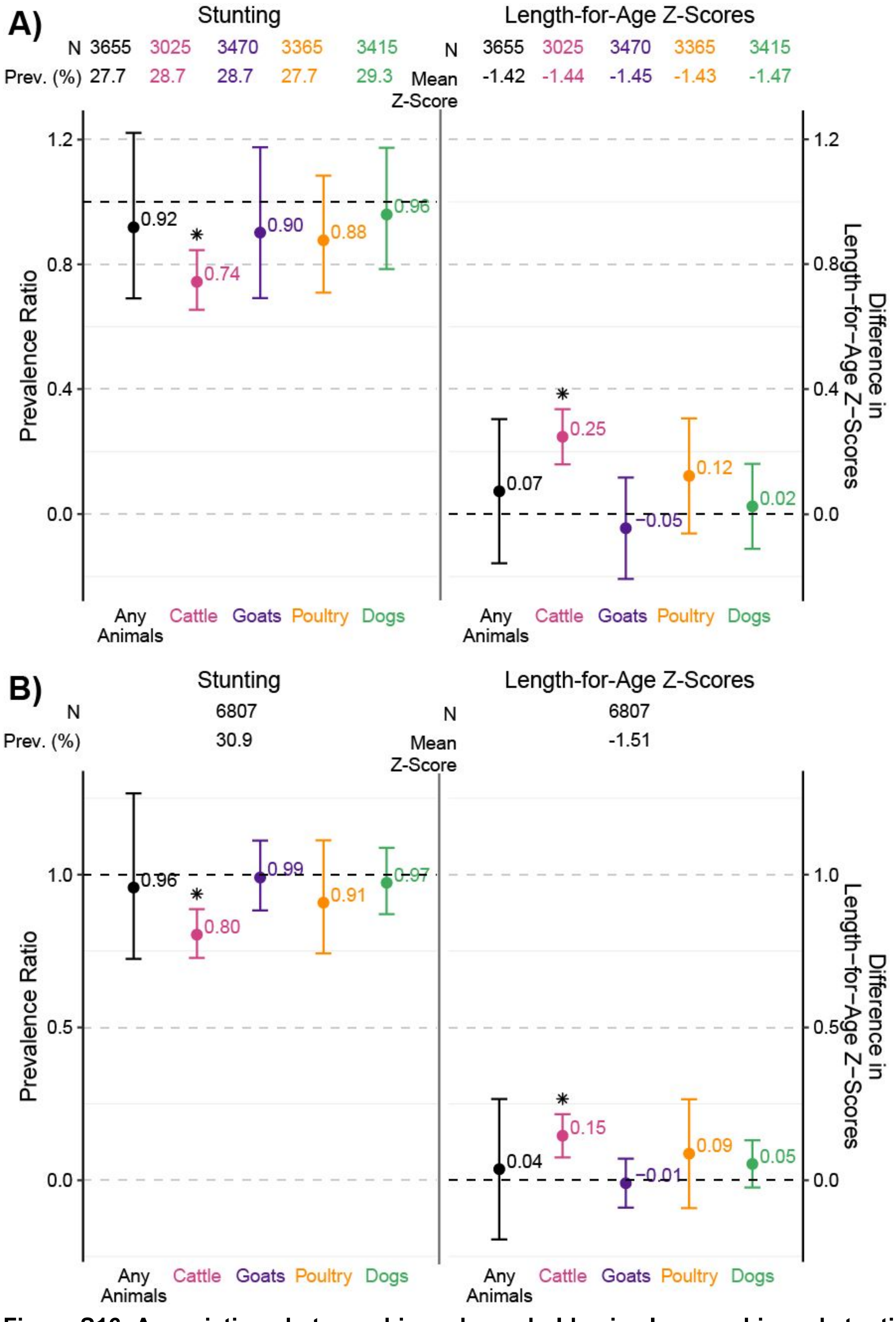

Figure S16: Associations between binary household animal ownership and stunting outcomes, accounting for households that changed animal ownership status between enrollment and year two. Prevalence ratios for stunting (left) and differences in length-for-age Z-scores (right) in animal-owning compared to non-animal-owning households, where (A) excludes households that changed ownership status and (B) reclassifies households that obtained animals during the study as animal-owning households at enrollment. Asterisks indicate significance at a significance level of 0.05.

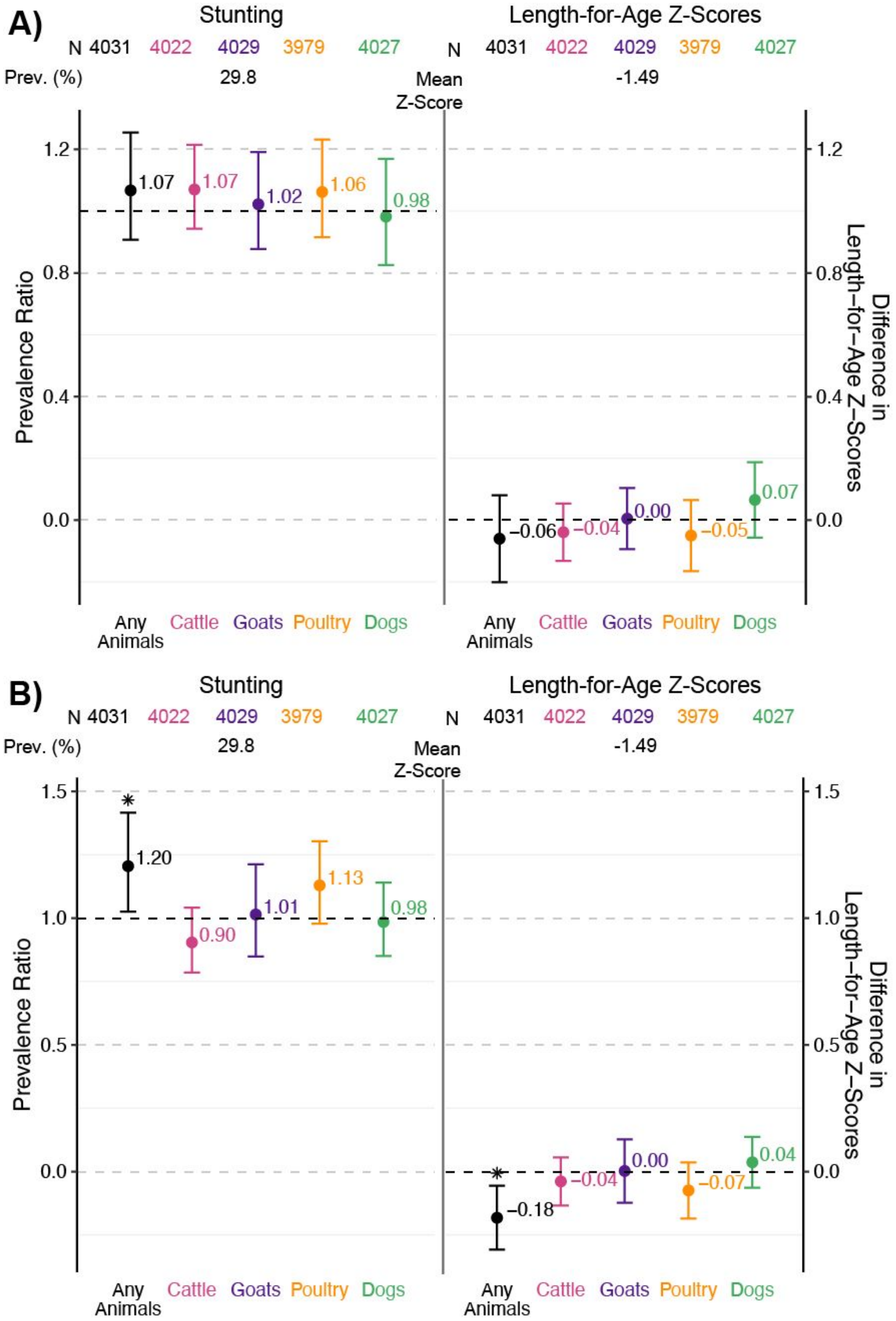

Figure S17: Associations between binary household animal ownership and stunting outcomes, accounting for subgroups of households that changed animal ownership status between enrollment and year two. Prevalence ratios for stunting (left) and differences in length-for-age Z-scores (right) for households that (A) obtained animals (compared to staying the same status or changing to non-animal-owning) and (B) went from animal- to non-animal-owning status (compared to staying the same or obtaining animals) during the study. Asterisks indicate significance at a significance level of 0.05.

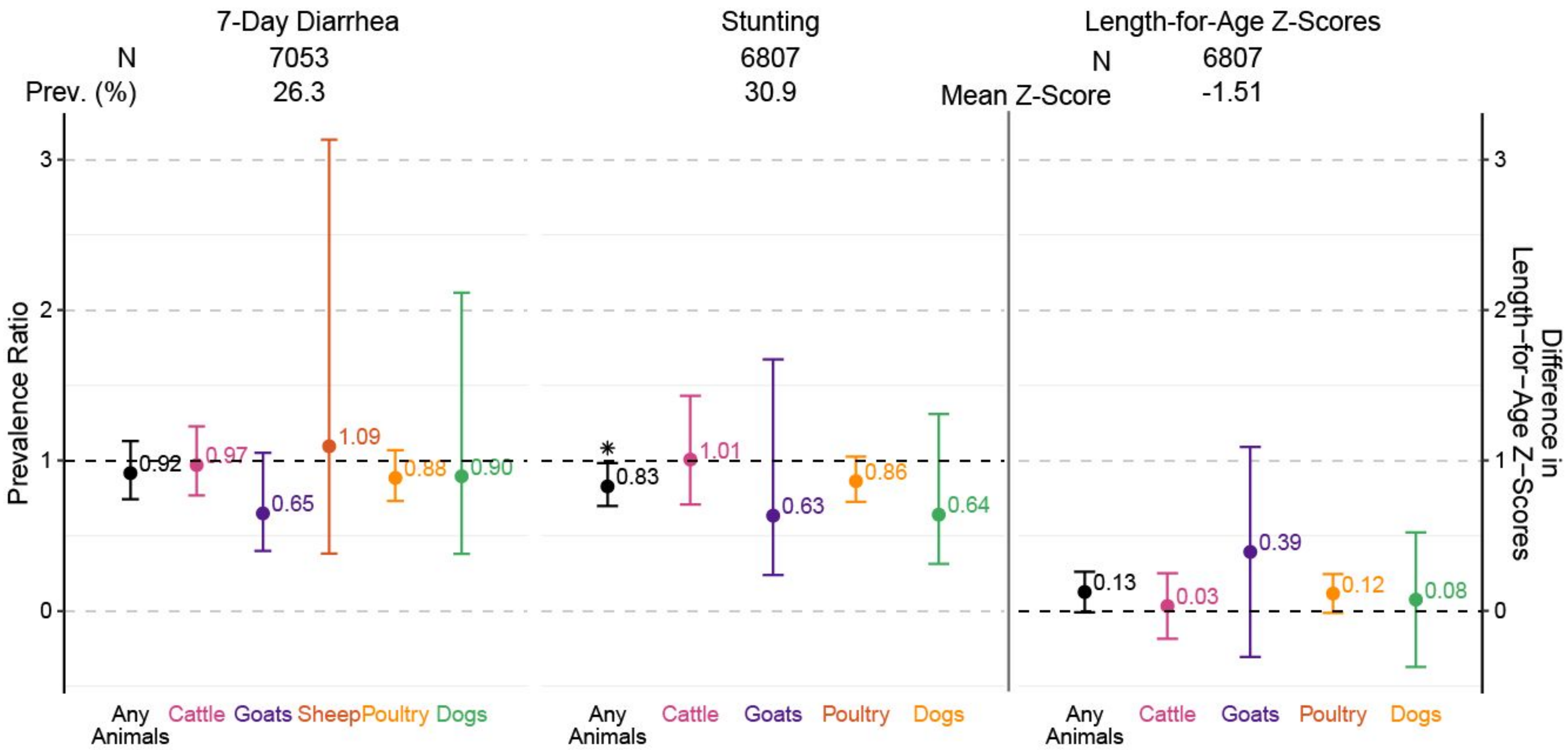

Figure S18: Associations between $\log_{10}$ median number of animals owned by households in the same village and child health. Prevalence ratios for diarrhea and stunting for each additional $\log_{10}$ animal owned (left); differences in length-for-age Z-scores for each additional $\log_{10}$ animal owned (right). Asterisks indicate significance at a significance level of 0.05.
